# Proteomics of protein trafficking by in vivo tissue-specific labeling

**DOI:** 10.1038/s41467-021-22599-x

**Published:** 2021-04-22

**Authors:** Ilia A. Droujinine, Amanda S. Meyer, Dan Wang, Namrata D. Udeshi, Yanhui Hu, David Rocco, Jill A. McMahon, Rui Yang, JinJin Guo, Luye Mu, Dominique K. Carey, Tanya Svinkina, Rebecca Zeng, Tess Branon, Areya Tabatabai, Justin A. Bosch, John M. Asara, Alice Y. Ting, Steven A. Carr, Andrew P. McMahon, Norbert Perrimon

**Affiliations:** 1 Department of Genetics, Blavatnik Institute, Harvard Medical School, Boston, MA USA; 2 Department of Stem Cell Biology and Regenerative Medicine, University of Southern California, Los Angeles, CA USA; 3 Eli and Edythe Broad Center for Regenerative Medicine and Stem Cell Research, University of Southern California, Los Angeles, CA USA; 4 Department of Entomology, China Agricultural University, Beijing, China; 5 Broad Institute of Harvard and MIT, Cambridge, MA USA; 6 Department of Electrical Engineering, Yale University, New Haven, CT USA; 7 Chan Zuckerberg Biohub, San Francisco, CA USA; 8 Department of Medicine, Harvard Medical School, Boston, MA USA; 9 Division of Signal Transduction, Beth Israel Deaconess Medical Center, Boston, MA USA; 10 Departments of Genetics, Biology, and Chemistry, Stanford University, Stanford, CA USA; 11 Howard Hughes Medical Institute, Boston, MA USA; 12 Present Address: Department of Molecular Medicine, Scripps Research, La Jolla, CA USA

**Keywords:** Proteomics, Proteomic analysis, Extracellular signalling molecules, Circulation

## Abstract

Conventional approaches to identify secreted factors that regulate homeostasis are limited in their abilities to identify the tissues/cells of origin and destination. We established a platform to identify secreted protein trafficking between organs using an engineered biotin ligase (BirA*G3) that biotinylates, promiscuously, proteins in a subcellular compartment of one tissue. Subsequently, biotinylated proteins are affinity-enriched and identified from distal organs using quantitative mass spectrometry. Applying this approach in *Drosophila*, we identify 51 muscle-secreted proteins from heads and 269 fat body-secreted proteins from legs/muscles, including CG2145 (human ortholog ENDOU) that binds directly to muscles and promotes activity. In addition, in mice, we identify 291 serum proteins secreted from conditional BirA*G3 embryo stem cell-derived teratomas, including low-abundance proteins with hormonal properties. Our findings indicate that the communication network of secreted proteins is vast. This approach has broad potential across different model systems to identify cell-specific secretomes and mediators of interorgan communication in health or disease.

## Abstract (web summary)

The network of proteins secreted for interorgan communication is poorly understood. Here, the authors develop a method, based on protein labeling, to study cell-specific secretomes and interorgan protein trafficking, and demonstrate their approach in *Drosophila* and mouse models.

## Introduction

Local tissue homeostasis is becoming increasingly well-understood. However, the physiological importance and presence of secreted blood and interorgan communication factors is only beginning to be documented from experiments in *Drosophila* and vertebrates. Secreted factors acting directly or indirectly between organs encode key regulators of systemic homeostasis^1^. These factors traffic, or translocate, intracellularly from their production sites within cells^2^ to distal organs through the blood circulation^1^. For example, adipokines such as leptin and adiponectin encode adipose tissue-derived systemic metabolic regulators that act on the brain and other organs^1^. In addition, myokines such as interleukin-6 are secreted by muscles and control metabolism in adipose tissue^1^. Despite their importance, the identification of interorgan communication factors is technically challenging, and a number of published results have been later determined to be irreproducible or controversial^1,3–5^. Also, origins and/or destinations of known factors including glucagon-like peptide 1 (GLP-1), ghrelin, leptin, cholecystokinin (CCK), and growth differentiation factor 11 (GDF-11) remain to be clarified^1,5^. Moreover, because large-scale screening methods are lacking, the long-standing biological question of how many proteins are transferred between any two given organs has yet to be addressed^1^. While a number of factors have been recognized in mammals, many physiologically-important factors likely remain to be identified^1^. In addition, defining the secretome of a given cell type directly in the in vivo setting is an important unmet need. *Drosophila* is a powerful model for interorgan communication, as organ systems and many physiological processes are conserved between flies and mammals. For instance, *Drosophila* has organs including the fat body (FB; functionally equivalent to the mammalian liver and adipose tissue) and muscles, and systemic factors with mammalian orthologs, including insulin, leptin, glucagon, transforming growth factor/activin-like, and tumor necrosis factor^1^. Thus, defining the secretome of *Drosophila* tissues would advance studies of interorgan communication in general.

Liquid chromatography-tandem mass spectrometry (LC–MS/MS), when used in a targeted manner, is a powerful approach to identify secreted factors in blood^6^. While LC–MS/MS is a sensitive method, unprocessed blood samples are exceedingly complex with large dynamic ranges of protein concentrations—the majority of the protein mass consists of a small number of protein species that dominate the MS signal, making identification of lower-abundance proteins challenging. Furthermore, LC–MS/MS of blood does not identify the origins and destinations of secreted proteins. Also, MS proteomics of cell culture supernatants is not physiological (because it is performed in vitro and, frequently, in serum-free media^7^) and cannot identify destinations of factors.

In this work, to overcome these limitations, we develop a method whereby secreted proteins from a specific tissue are labeled by biotin in vivo using the engineered promiscuous biotin ligase BirA*G3 (a relative of TurboID^8^), then collected by affinity-enrichment from distal organs and identified by quantitative LC–MS/MS. Using this approach, we simplify the proteome under study to the most relevant protein candidates involved in interorgan trafficking and determine their origins and destinations. Using this platform in *Drosophila*, we identify 51 putative muscle-secreted proteins from heads and 269 FB-secreted proteins from legs/muscle organ (here defined as muscle tissue and innervations/neuromuscular junctions (NMJs)), of which 60–70% have human orthologs. Among these are FB signaling proteins that have known receptors and/or were previously demonstrated to bind to muscle organs with specific patterns. We demonstrate, in particular, that a conserved FB-derived interorgan communication factor CG2145 (human ortholog: ENDOU) promotes muscle activity and binds directly and with a specific pattern to the muscle organ (near muscles/neurons), but not to other organs. These results suggest that our approach can identify specific interactions and remote action in muscles by FB-produced proteins. To examine the potential of this approach in mammalian systems, a conditional BirA*G3 allele is generated through targeting of mouse embryo stem cells (ESCs), and biotinylation analyzed in ESC-derived teratomas and host serum samples. Quantitative tandem mass-tag (TMT) mass spectrometry identifies biotin-dependent labeling of secreted proteins shared between the tumor and serum samples. Among 291 streptavidin-enriched blood serum proteins from BirA*G3 tumors are several low-abundance proteins with hormonal properties. Our findings indicate that the communication network of secreted proteins is extensive, and we provide a resource for candidate interorgan communication factors. The BirA*G3 approach has broad potential across different model systems to identify cell secretomes and mediators of interorgan communication in healthy or diseased states.

## Results

### Defining the origins and destinations of positive control and additional interorgan communication proteins

Biotinylation by the *E. coli* promiscuous biotin protein ligase (BirA*; “*” means promiscuous), BirA*R118G mutant^9,10^, or our engineered high-activity BirA*G3 (third generation evolved mutant, a relative of TurboID^8^), is continuous and mild, only requiring the addition of biotin to culture media^10^, potentially allowing long-term protein fate tracking (Supplementary Discussion). We developed the method in *Drosophila* and generated strains carrying UAS-BirA*R118G-ER and UAS-BirA*G3-ER to express BirA* in the endoplasmic reticulum (ER) for biotinylation of conventionally secreted proteins. In addition, to detect unconventionally secreted proteins^2^, we generated untagged UAS-BirA*R118G and UAS-BirA*G3 lines for biotinylation of proteins in the cytoplasm/nucleus. When expressed using a tissue-specific *Gal4* and biotin feeding, BirA* is expected to biotinylate proteins in the ER or cytoplasm/nucleus of cells within any chosen organ. Expression of UAS-BirA*G3-ER using ubiquitous *TUB-Gal4* or FB-specific *LPP-Gal4* drivers (see “Methods” section) and biotin feeding did not have statistically significant effects on lifespan or climbing-ability (Supplementary Fig. 1a–i), suggesting that biotinylation does not severely affect organismal function, thereby enabling long-term studies. We did not find evidence for the presence of major biotin-binding proteins in the hemolymph (Supplementary Fig. 1j), suggesting that proteins biotinylated by BirA* should be able to traffic normally between organs.

**Fig. 1:**
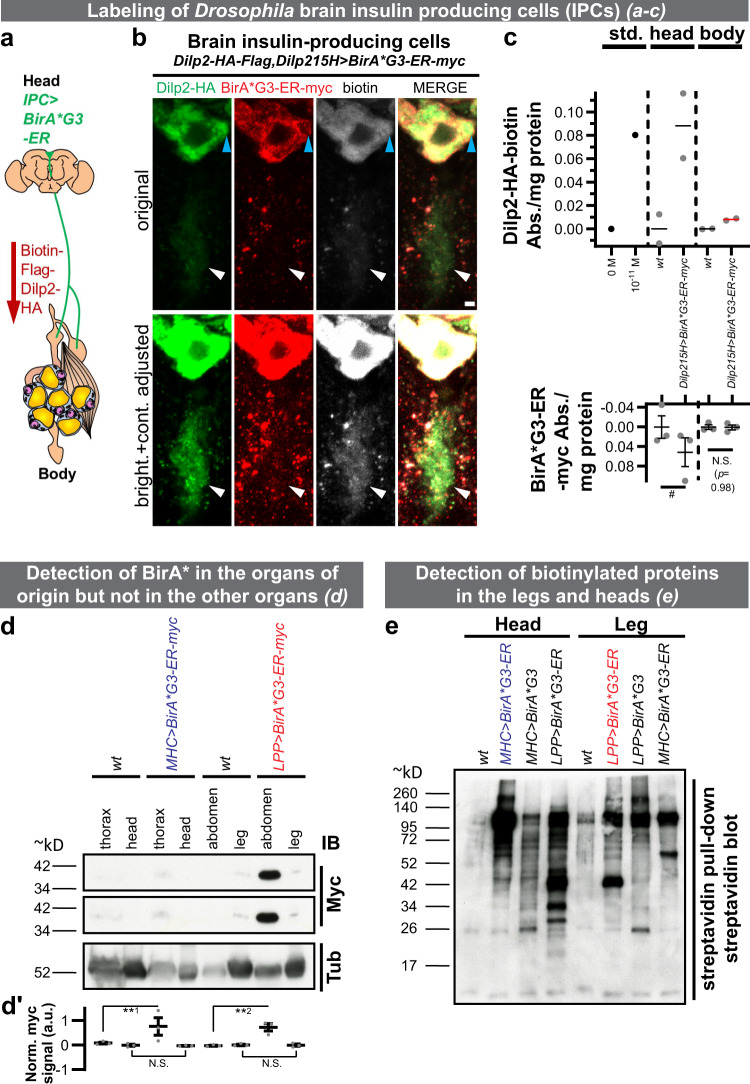
Labeling of secreted proteins and their detection in distal organs. **a** Schematic of the biotinylated *Drosophila* insulin-like peptide 2 (Dilp2) experiment. BirA*G3-ER (endoplasmic reticulum) was expressed in the insulin-producing cells (IPCs). **b** BirA*G3-ER^+^ (red), Biotin^+^ (white), Dilp2-HA^+^ (green)^11^ cell bodies, and BirA*G3-ER^negative^, biotin^+^ and Dilp2-HA^+^ parts of the cell body (blue arrowhead) and ventral projections (which include dendrites and axons, which extend further^13^; white arrowhead) were detected. Scale bar: 2 µm. **c** Spatially informed bead enzyme-linked immunosorbent assay (ELISA) of biotinylated Dilp2-HA trafficking from head. Data are mean ± SEM absorbance (Abs.). Top: *n* = 2 biological replicates. Std. means standard peptide (1.04 × 10^−11^ M standard biotin-Flag-GS-HA peptide (*n* = 1)). The mean of Dilp215H>BirA*G3-ER-myc body is shown with the red line. Bottom: *n* = 3 biological replicates; two-tailed t-test (^#^*p* = 0.08996). N.S. means not significant. Approximate locations of IPC axonal projections in the cartoon diagram are based on ref. ^14^. **d** Fat body (FB)-expressed LPP-Gal4>BirA*G3-ER-myc is detected in abdomens but not legs, and muscle-expressed MHC-Gal4>BirA*G3-ER-myc is detected in thoraxes but not heads. Middle panel: higher exposure of the myc blot. IB: immunoblot. See Supplementary Fig. 2bb for the parts of legs used. For each slice, all lanes are from the same blot (see Source Data for uncropped blots). **d′** Quantification of signals from *n* = 3 independent experiments. Statistics: mean ± SEM integrated density of myc signals, normalized to tubulin (Tub) staining; one-way ANOVA and Benjamini, Krieger, Yekutieli linear two-stage step-up FDR. **^1^*p* = 0.0038; **^2^*p* = 0.0035; N.S. (*p* = 0.51). **e** Streptavidin bead pulldown followed by streptavidin-HRP detection in the leg and head lysates after FB and muscle biotinylation using BirA*G3-ER or BirA*G3 (cytoplasmic/nuclear for unconventionally secreted proteins). All lanes are from the same blot (see Source Data for uncropped blots). Representative result of three western blots. In **d**, **e** MHC>BirA*G3-ER and LPP>BirA*G3-ER are shown in color for clarity. In **b**–**e** flies were maintained with 50 µM biotin in food during adulthood. *wt* means wild-type. See also: Supplementary Figs. 1–3. Source data are provided as a Source Data file.

As a proof-of-concept, we expressed UAS-BirA*G3-ER-myc using the brain insulin-producing cell (IPC)-specific *Dilp215H-Gal4*^11^ driver (Dilp2: *Drosophila* insulin-like peptide 2). IPCs are median neurosecretory cells (mNSCs) within the protocerebrum region of the brain^12^, and they have ventral projections that comprise both dendrites and axons^13^. A major source of Dilps, the axons extend further to the anterior gut, the ring gland, and the crop (schematic in Fig. 1a)^14^. In the IPCs within the brain, we detected overlapping Dilp2^11^, BirA*G3-ER, and biotinylated proteins signals (Supplementary Figure 2a–h). We observed BirA*G3-ER-negative, but Dilp2/biotin double-positive areas in IPC soma (marked with a blue arrowhead) and ventral projections (white arrowhead; Fig. 1b). This suggests that labeled Dilp2 originates from BirA*G3-ER-positive production sites within the IPCs. Further, we developed a single-antibody bead enzyme-linked immunosorbent assay (ELISA; Supplementary Fig. 2q–r). Using this assay, we detected biotinylated Dilp2 in heads and body, and BirA*G3-ER in heads but not body (Fig. 1c). This suggests that the biotinylated protein (Dilp2), but not the biotinylating enzyme (BirA*G3-ER), is detectable in the body and IPC axons within the body^14^. We conclude that our approach can label IPCs and detect low-abundance^11^ Dilp2 in body parts with no detectable BirA*. Thus, another application of our system is to enable spatially informed ELISA for proteins with only one available antibody—desirable for small or difficult antigens.

To further verify that we can controllably express BirA*G3-ER in different tissues without undesirable secretion of the enzyme, we expressed *LPP-Gal4*>BirA*G3-ER in FB and detected BirA*G3-ER in abdominal FB (Supplementary Fig. 2s–z; signals quantified in Supplementary Fig. 2aa), but not legs (Fig. 1d; signals quantified from three independent blots in Fig. 1d′). For western blot and LC–MS/MS analyses, we used the parts of legs that do not have detectable *LPP-Gal4* expression (Supplementary Fig. 2bb; see “Methods” section). In addition, we expressed BirA*G3-ER in muscles (*MHC-Gal4*>BirA*G3-ER) and detected BirA*G3-ER in thoracic muscles (Supplementary Fig. 2aa–hh) but not heads (Fig. 1d; quantified in Fig. 1d′). Also, we expressed MHC-Gal4>BirA*G3 in muscles and detected BirA*G3 and biotinylated proteins in thoracic muscles (Supplementary Fig. 2ii–pp). In legs, biotinylation patterns in *wild-type* (*wt*), LPP-Gal4>BirA*G3-ER (labels proteins secreted from ER), LPP-Gal4>BirA*G3 (cytoplasmic and nuclear unconventionally secreted proteins), and *MHC-Gal4*>BirA*G3-ER (leg muscles ER-resident proteins) differed. Differences in biotinylation were also observed in heads, between *wt*, *MHC-Gal4*>BirA*G3-ER, *MHC-Gal4*>BirA*G3, and LPP-Gal4>BirA*G3-ER (head FB ER-resident proteins) (Fig. 1e; extended set of samples shown in Supplementary Fig. 3a). In summary, FB-secreted proteins found in the legs, muscle-secreted proteins found in heads, and ER- and cytoplasm-derived proteins differ significantly. This is consistent with secretome differences of organs^1^ and organelles (ER versus cytoplasm)^2^. Furthermore, using the original, less active *R118G* biotin ligase BirA*R118G and BirA*R118G-ER, we detected FB-secreted proteins in legs, muscle-secreted proteins in the head, FB- and muscle-secreted proteins in the hemolymph (fly blood), and FB-secreted proteins in the brain, without detecting BirA*R118G or BirA*R118G-ER in distal tissues (Supplementary Fig. 3b–e). Altogether, these results demonstrate that the BirA* approach is able to detect secreted proteins in distal organs, and show its usefulness for identifying candidate proteins that traffic between multiple organs.

**Fig. 2:**
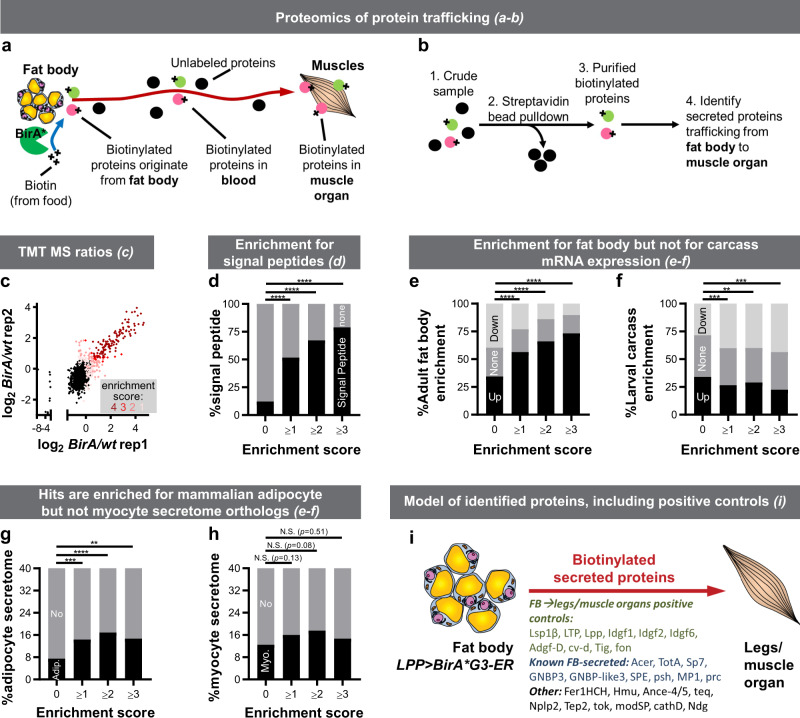
Identification of fat body (FB)-derived proteins in legs/muscle organ. **a** Promiscuous biotin ligase BirA*R118G or our newly engineered high-activity BirA*G3 biotinylates all proteins in a subcellular compartment of one organ and the biotinylated proteins traffic to other organs or body parts. **b** Biotinylated proteins are enriched and identified by mass spectrometry. **c**–**i**, FB was labeled using LPP-Gal4>BirA*G3-ER (endoplasmic reticulum), and biotinylated proteins from legs/muscle organs were analyzed by tandem mass-tag (TMT) mass spectrometry (MS). *Wild-type*(*wt*) legs were used as controls. See Supplementary Fig. 2bb for the parts of legs used. Flies were maintained with 50 µM biotin in food during adulthood. **c**, Leg log_2_(BirA*G3-ER/wt) TMT ratios in two replicates. Each point is *n* = 2 comparisons, mean log_2_TMT ratio. Enrichment score (E-S): number of comparisons (from 4) in which TMT-ratio > threshold (score 4 [dark red] is for most confident hits and 0 [black] is background). **d** As the E-S increases, the fraction of proteins with putative signal peptides (see “Methods” section) increases. E-S = 0 vs E-S ≥ 1: ****p = 7.00 · 10^−57^; E-S = 0 vs E-S ≥ 2: ****p = 1.95 · 10^−75^; E-S = 0 vs E-S ≥ 3: ****p = 2.17 · 10^−82^. **e**, **f** As the E-S increases, fraction of proteins enriched for adult-FB mRNA microarray^15^ expression increases (**e**), and the fraction of proteins enriched for larval carcass (FB-free muscle organ data set) FB mRNA microarray^15^ decreases (**f**). E-S = 0 vs E-S ≥ 1: ****p = 2.78 · 10^−12^; E-S = 0 vs E-S ≥ 2: ****p = 3.67 · 10^−17^; E-S = 0 vs E-S ≥ 3: ****p = 4.53 · 10^−18^. **g**, **h** Hits are enriched for mammalian adipocyte (**g**) but not myocyte (**h**) secretome orthologs (see “Methods” section). ****p = 0.00007, ***p = 0.0004, **p = 0.0090. **i** Model of identified proteins known to traffic from FB to imaginal discs or muscle organ and known and additional FB-secreted proteins (see “Methods” section). In **d**–**h** a chi square test was used. N.S. means not significant. In **d**, **g**, **h** the test was two-sided, while in **e**, **f** the contingency table had three outcomes (up, none, or down), and one- or two-sided nature of the test was not applicable. See also: Supplementary Figs. 4–8, Supplementary Tables 3–7, Supplementary Data 1–3. Source data are provided as a Source Data file.

We used BirA*G3-ER and LC–MS/MS with mass-tag-labeling of peptides using TMT reagents to identify and quantify, in a single experiment, FB- and muscle-derived proteins in legs and heads, respectively (Fig. 2a, b and Supplementary Fig. 4a; Supplementary Data 1). We focused on this model organ system because legs and heads can be collected in large numbers (see “Methods” section), these interorgan communication axes are poorly-characterized, and FB-derived proteins present in legs may be involved in metabolic regulation of muscle activity^1^. MS-identified proteins in legs were first compared to positive control (PC) secreted/receptor and negative control (NC) intracellular lists (Supplementary Fig. 4b; see “Methods” section). For each of the four BirA*G3-ER/wt TMT-ratio comparisons, we determined threshold TMT ratios for hit-calling where $\frac{{\#{\mathrm{PC}}}}{{\#{\mathrm{PC}}}+{\#{\mathrm{NC}}}} \,> \, 0.9$ (Supplementary Fig. 4c). From this, for each protein, we calculated an “enrichment score” (E-S), defined as the number of TMT-ratio comparisons in which the protein TMT-ratio exceeded the threshold (i.e., enriched in the BirA*G3-ER sample; see “Methods” section). Hence, proteins with higher average TMT ratios have higher E-S, highest confidence hits have E-S of 4, background proteins (non-hits) have E-S of 0 (Fig. 2c and Supplementary Fig. 4d), and as expected, hits are enriched for PC proteins (Supplementary Fig. 4e). The biological replicates show good agreement in TMT-ratio signals (Fig. 2c).

Our analysis revealed that higher E-S correlates significantly with more signal peptide-containing proteins, suggesting that our hits are secreted (Supplementary Fig. 4f and Fig. 2d; see “Methods” section). Specifically, of the top 107 ranked predictions with an E-S of 4, 83% have a signal peptide. Consistently, hits are enriched for proteins found in whole fly hemolymph MS (Supplementary Fig. 4g–i and Supplementary Data 2, Supplementary Table 3). As negative controls, known ER-resident and predicted unconventionally secreted proteins were not enriched (Supplementary Fig. 5a, b; see “Methods” section). We estimated protein abundances using the PAX database (see “Methods” section), and determined that increased E-S showed enrichment for lower whole-body abundance proteins (Supplementary Fig. 5c–f), likely reflecting the isolation of the most relevant proteins away from abundant contaminants using biotinylation.

Interestingly, as the E-S increases, the fraction of hits that were enriched for adult-FB mRNA microarray expression^15^ increases (Fig. 2e). Consistently, overlaps with larval FB microarray and larval/pupal FB RNAseq data sets also increase (see “Methods” section). In addition, increased E-S results in decreased enrichment in larval carcass mRNA (data set of FB-free muscle organ^15^—here defined as muscle tissue and innervations/neuromuscular junctions (NMJs); Fig. 2f). Thus, our data set is enriched for genes transcribed in FB but not in muscle organ, suggesting of specific FB labeling, and that these proteins are candidates for trafficking from FB to legs/muscle organ. Moreover, hits are enriched for mammalian adipocyte secretome orthologs, but not for myocyte secretome orthologs (Fig. 2g, h; see “Methods” section), suggesting that some of our hits may be orthologs of previously-identified adipocyte secreted proteins.

Similar MS analysis identified muscle-secreted proteins harvested from heads (Supplementary Figs. 4a and 6; Supplementary Data 1), and comparison with FB-to-legs data set revealed limited overlap, suggesting organ specificity in labeling and interorgan secretome trafficking (Supplementary Fig. 6j–k). Furthermore, hits from the FB-to-legs LPP-Gal4>BirA*G3-ER data set show statistically significant overlap with hits from similarly-analyzed LPP-Gal4>BirA*R118G-ER MS data set (first generation, less active BirA*; Supplementary Fig. 7a–g; Supplementary Data 3), compared to background (Supplementary Fig. 7h).

Overall, using LPP-Gal4>BirA*G3-ER, we identified 269 FB ER-derived proteins (corresponding to 266 genes) present in legs, including growth factors, binding proteins, enzymes, peptidases (Supplementary Fig. 8), and 98 proteins with poorly-characterized functions (“Computed Genes”/CGs). We were able to detect FB-to-legs positive controls known to traffic from FB to muscle organ and/or imaginal discs, including lipid-binding proteins Lsp1β, LTP, Lpp; growth factors Idgf1/2/6 and Adgf-D; TGF-β binding protein cv-d; extracellular-matrix protein Tig; and the muscle adhesion protein fon (Fig. 2i; details and references in Supplementary Table 4). Moreover, we identified additional secreted proteins previously unknown to be produced by FB or to traffic to legs or the muscle organ (Fig. 2i; Supplementary Table 5 and 6). Our data includes proteins previously shown to interact with the muscle organ with specific patterns, and/or which have known receptors. For example, Tig binds to its PS2-integrin receptor at muscle adhesion foci and on the muscle surface Z-bands^16–18^. Also, FB-secreted fon has been previously shown to bind with a specific pattern to muscle adhesion foci and to regulate their functions^18^. In addition, Lpp and LTP interact with lipophorin receptors^19,20^, and FB-derived prc binds to heart muscle extracellular-matrix through its receptor loh^21^ (Supplementary Tables 4 and 5). Hence, our data show that we are able to identify known and additional FB-specific proteins present in the legs or muscle organs. Notably, 60–70% of all hits and 50–60% of proteins with poorly-characterized functions (CGs) are well-conserved to humans (DIOPT score of at least 3^22^). We identified *Drosophila* orthologs of human secreted proteins involved in systemic pathways, including ACE, APOB, TLL1, BMP1, LNPEP, TRHDE, ENPEP, ECE1, MME/NEP, KLKB1, CD109, and CTSD (details and references in Supplementary Table 7). Characterization of the orthologs will provide future insights into mammalian physiology.

**Fig. 3:**
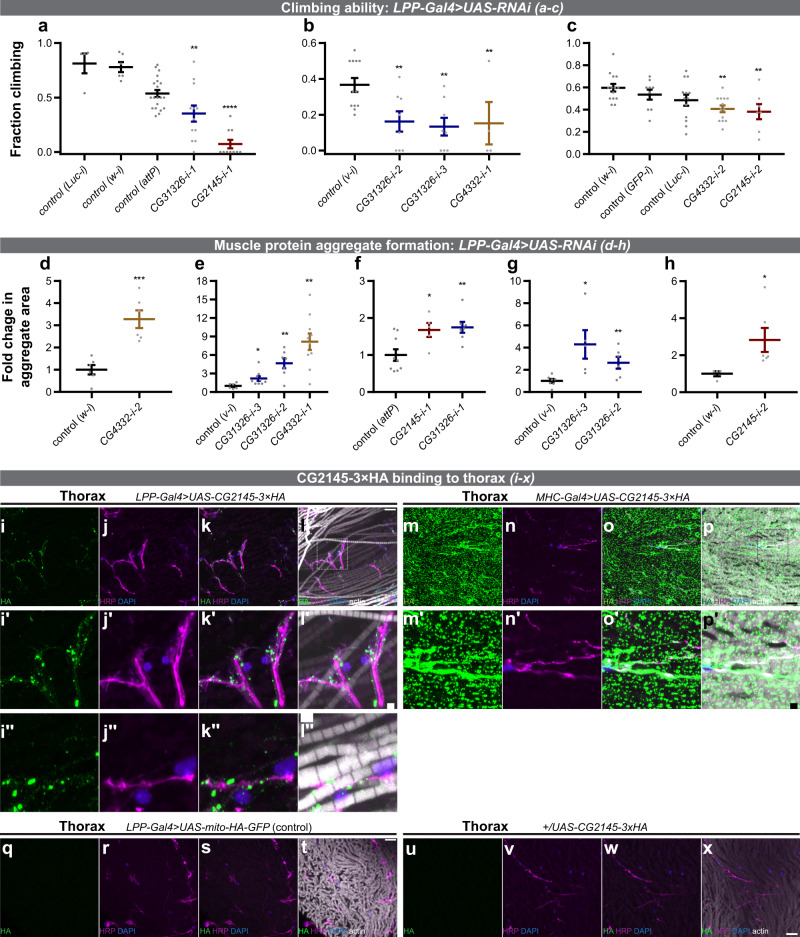
Muscle functions of legs/muscle proteins derived from the fat body (FB). **a**–**c** Adult flies with FB RNAi (using *LPP-Gal4*) against *CG4332*, *CG2145*, and *CG31326* have reduced climbing-ability. **a** *LPP-Gal4>RNAi* from VDRC (Vienna *Drosophila* Resource Center) at 3 weeks old and 29 °C. Biological replicates: *n* = 4 (*Luc-i*), *n* = 6 (*w-i*), *n* = 20 (*attP*), *n* = 12 (*CG31326-i-1*; blue), *n* = 10 (*CG2145-i-1*; red). Relative to control (*attP*): **p = 0.0024, ****p = 1.10 · 10^−11^. **b** *LPP-Gal4>RNAi* from NIG (Japan National Institute of Genetics) at 5 weeks old and 27 °C. Biological replicates: *n* = 12 (*v-i*), *n* = 8 (*CG31326-i-2*; blue), *n* = 8 (*CG31326-i-3*; blue), *n* = 4 (*CG4332-i-1*; yellow). Relative to control (*v-i*): **p = 0.0037 (*CG31326-i-2*), **p = 0.0011 (*CG31326-i-3*), **p = 0.0090 (*CG4332-i-1*). **c** *LPP-Gal4>RNAi* from TRiP (Harvard Transgenic RNAi Project) at 5 weeks old and 27 °C. Biological replicates: *n* = 14 (*w-i*), *n* = 9 (*GFP-i*), *n* = 13 (*Luc-i*), *n* = 13 (*CG4332-i-2*; yellow), *n* = 7 (*CG2145-i-2*; red). Relative to control (*w-i*): **p = 0.0021, **p = 0.0031. Statistics: mean ± SEM; one-way ANOVA and Benjamini, Krieger, Yekutieli linear two-stage step-up FDR. **d**–**h** Adult flies with FB RNAi (using *LPP-Gal4*) against *CG4332*, *CG2145*, and *CG31326* have increased muscle protein aggregate formation. Area of p62/ref(2)p-positive protein aggregates was quantified, and normalized and compared to controls. **d** *LPP-Gal4>RNAi* (TRiP), 3 weeks old at 27 °C. *n* = 6 (*w-i*), *n* = 6 (*CG4332-i-2*; yellow). ***p = 0.00052. **e** *LPP-Gal4>RNAi* (NIG), 3 weeks old at 27 °C. *n* = 6 (*v-i*), *n* = 9 (*CG31326-i-3*; blue), *n* = 7 (*CG31326-i-2*; blue), *n* = 10 (*CG4332-i-1*; yellow). *p = 0.029, **p = 0.0017 (*CG31326-i-2*), **p = 0.0011 (*CG4332-i-1*). **f** *LPP-Gal4>RNAi* (VDRC), 3 weeks old at 29 °C. *n* = 9 (*attP*), *n* = 5 (*CG2145-i-1*; red), *n* = 7 (*CG31326-i-1*; blue). *p = 0.021, **p = 0.0046. **g** *LPP-Gal4>RNAi* (NIG), 5 weeks old at 27 °C. *n* = 7 (*v-i*), *n* = 5 (*CG31326-i-3*; blue), *n* = 6 (*CG31326-i-2*; blue). *p = 0.012, **p = 0.0094. **h** *LPP-Gal4>RNAi* (TRiP), 5 weeks old at 27 °C. *n* = 4 (*w-i*), *n* = 6 (*CG2145-i-2*; red). *p = 0.041. Statistics: mean ± SEM; two-tailed t-test. **i**–**x** FB-derived CG2145 binds to a specific pattern to the thoracic muscle organ (near muscles/neurons within muscle organ). Thoraxes from *LPP-Gal4>UAS-CG2145-3xHA* (**i**–**l**), *MHC-Gal4>UAS-CG2145-3xHA* (**m**–**p**; muscle-driven CG2145-3xHA expression, positive control), *LPP-Gal4>UAS-mito-HA-GFP* (**q**–**t**; negative control), and *+/UAS-CG2145-3xHA* (**u**–**x**; UAS-only non-expressing control) flies were stained for HA (green), neurons (HRP; magenta), nuclei (DAPI; blue), and actin (phalloidin; white; see “Methods” section). Representative maximum intensity projections are shown from 12 images across 4 thoracic samples (**i**–**l**), 3 images across 2 thoracic samples (**m**–**p**), 2 images across 2 thoracic samples (**q**–**t**), and 3 images across 2 thoracic samples (**u**–**x**). For images in **l,** **l′, l′′**, brightness and contrast for the actin (phalloidin) channel were adjusted equally within the image to visualize actin structures; all other channels were not adjusted. See Supplementary Fig. 10 for the absence of CG2145 binding to other organs, respectively. (**i′**–**l′**) are zoomed-in images from the white rectangle in (**i**–**l**), and (**i′′**–**l′′**) are zoomed-in images from the blue rectangle in (**i**–**l**). (**m′**–**p′**) are zoomed-in images from the blue rectangle in **m**–**p**. In *MHC*–*Gal4>UAS-CG2145-3xHA* samples, the concentration of CG2145-3xHA signal can be observed around neurons, as well as outside of neurons, which are likely CG2145-3xHA production sites. Scale bar: 10 µm (**i**–**l**, **m**–**p**, **and q**–**x**); 2 µm (**i′**–**l′**, **I′′**–**l′′**, **and m′**–**p′**). See also: Supplementary Fig. 9-10, Supplementary Table 8, Supplementary Movies 1–3. See Supplementary Fig. 9x for the cartoon schematic of the locations of imaging within the fly. Source data are provided as a Source Data file.

The FB directly affects muscle organ metabolism and physiology^23–25^, yet the secreted proteins involved are largely unknown^1^. Climbing-ability assays and immunohistochemical readouts of autophagy through the extent of ubiquitin- and p62/ref(2)p-positive protein-aggregation are established indicators of *Drosophila* adult muscle function^26^. Thus, we used FB-specific *LPP-Gal4>RNAi* to determine whether candidates from our FB-to-legs MS list (Supplementary Data 1) have effects on muscle function. In our FB-to-legs data set, we detected a signal peptide-containing protein (see “Methods” section)—CG2145—whose knockdown in FB using two independent RNAi lines resulted in muscle phenotypes of poor climbing-ability and increased areas of protein aggregates (Fig. 3a, c, f, h and Supplementary Fig. 9g–h, m, n; Supplementary Table 8; Supplementary Movie 1; see Supplementary Fig. 9x for the cartoon schematic of the locations of imaging within the fly). As controls, we tested *CG2145* FB-RNAi phenotype in FB, and did not find significant effects on FB lipid-droplets (Supplementary Fig. 9q, s). In addition, we used *Dmef2-Gal4* to perform RNAi of CG2145 in muscles and did not observe statistically significant climbing-ability defects in either of two RNAi lines (Supplementary Fig. 9t, v), suggesting that, at least for climbing activity, muscles are not a significant source of CG2145. Interestingly, *CG2145-mRNA* is enriched in adult-FB over muscles (larval carcass)^15^. As further evidence suggesting that our BirA* approach may identify specific and remotely acting factors on the muscle organ, we expressed CG2145-HA-tag specifically in FB (see “Methods” section), and using confocal microscopy demonstrated that CG2145 binds strongly and with a specific pattern to thoracic muscle organ (near muscles/neurons within muscle organ; Fig. 3i–x; Supplementary Fig. 9x). However, FB-derived CG2145 does not bind to abdominal body wall muscles, central brain, or optic lobe, indicating of specificity of CG2145 binding to the thoracic muscle organ (Supplementary Fig. 10). This is a previously-undescribed binding pattern for FB-derived secreted factors. A putative human ortholog of CG2145 is ENDOU (poly-U-specific placental endonuclease), which is a signal peptide-containing RNA-binding protein of uncharacterized function in muscles or adipose tissue (Supplementary Table 8)^27–29^.

### Secretion of biotinylated proteins from BirA*G3-ER-expressing teratomas into serum in mice

**Fig. 4:**
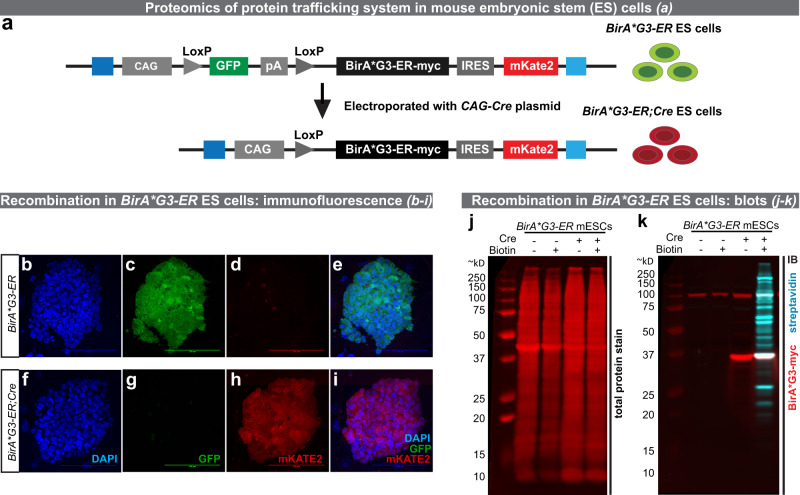
*Rosa26* (*R26*) BirA*G3–*ER* and R26 BirA*G3–*ER;Cre* 3A mouse embryonic stem cell (mESCs) characterization. **a** Schematic showing Cre-induced recombination of the R26 BirA*G3-ER allele to generate the BirA*G3-ER;Cre expressing cell line. GFP is in blue, BirA*G3-ER-myc is in black, mKate2 is in red, homologous regions to the *R26* locus are in blue, and other elements are colored in gray. **b**–**i** Native immunofluorescence in BirA*G3-ER parental (GFP^+^ (green)/mKate2^−^) or Cre-recombined (GFP^−^/mKate2^+^(red)) ESC colonies. DAPI (nuclei) is in blue. Representative images of four repeats. Scale bar: 100 µm. **j**, **k** Western blot analysis of mESC whole cell lysate with or without Cre recombination. **j** Total protein stain (red). **k** Western blot probed with streptavidin (cyan) or an anti-myc (red) antibody to detect myc-tagged BirA*G3. For each image, all lanes are from the same blot (see Source Data for uncropped blots). Representative results of three western blots. See also: Supplementary Fig. 11. Source data are provided as a Source Data file.

**Fig. 5:**
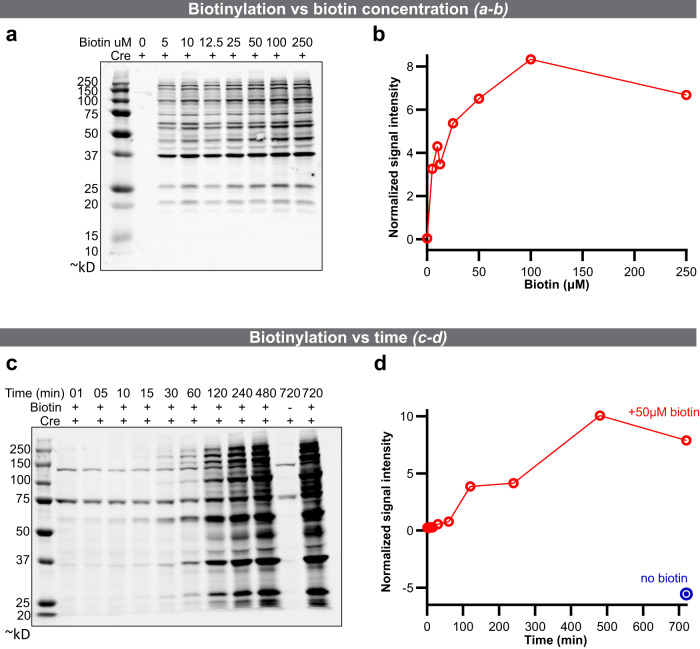
Characterization of in vitro biotinylation in the BirA*G3–*ER* mouse embryonic stem cell (mESC) line. **a** Western analysis of streptavidin binding to biotinylated proteins following 24 h of labeling at the indicated concentrations of biotin. All lanes are from the same blot (see Source Data for uncropped blots). **b** Quantification of biotin labeling relative to whole protein in **a**. **c** Western analysis of streptavidin binding to biotinylated proteins labeled in 50 μM biotin for the indicated times. All lanes are from the same blot (see Source Data for uncropped blots). **d** Quantification of biotin labeling relative to total protein in **c**. In this figure, representative results are shown from two western blots each. Source data are provided as a Source Data file.

To transition the BirA* approach to mammals, we generated mouse embryonic stem cells (mESCs) with a Cre-inducible mouse codon-optimized BirA*G3-ER inserted into the Rosa26 “safe-harbor” locus (CAGGS-GFP/BirA*G3-ER-mKate2(R26); Fig. 4a). These cells show efficient Cre-dependent biotinylated protein secretion in vitro, including a positive control, Alpl, as well as Cre-dependent BirA*G3-ER expression (Fig. 4b–k and Supplementary Fig. 11). To further characterize BirA*G3-ER;Cre cells, mESCs or mESC-derived fibroblast-like cells were cultured in varying concentrations of biotin (0–250 μM biotin; Fig. 5a, b) or in 50 μM biotin for different lengths of time (1 min up to 12 h; Fig. 5c, d). Optimal labeling was observed incubating cells in 50–100 μM biotin for 4–8 h, but significant levels of biotinylation were observed within 5 min of biotin addition and 1 h of labeling. We further used a fluorescent western blot to show that secretion of *Alp*l from mESCs is not significantly affected by BirA*G3-ER expression and activity (Supplementary Fig. 11ee).

**Fig. 6:**
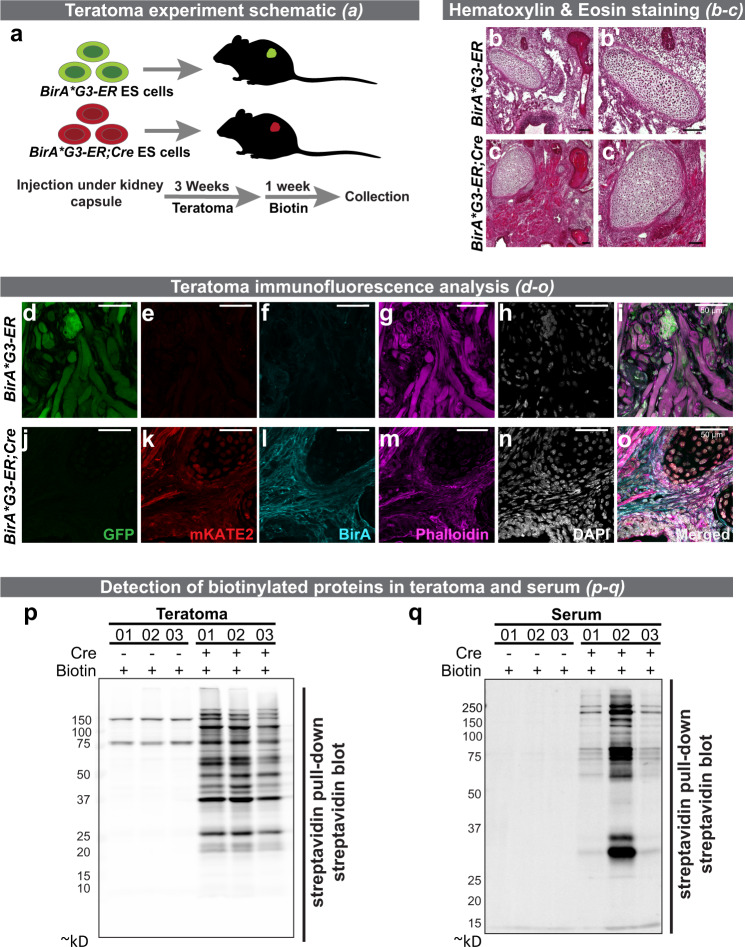
Analysis of biotin labeling of proteins in vivo in a mouse embryonic stem cell (mESC)–derived teratoma model. **a** Schematic representation of the teratoma study. GFP+ BirA*G3-ER mESC are shown in green, and mKate2+ BirA*G3-ER;Cre mESC are in red. **b**–**c** Hematoxylin and eosin staining of cryo-sectioned teratoma tissue. **b′**, **c′** High magnification images. Representative results of three replicate images. Scale: 100 µm. **d**–**o** Analysis of reporter gene activity and BirA*G3 in cryo-sections of teratoma tissue. Scale bar: 50 µm. Green is GFP, red is mKate2, cyan is BirA, magenta is phalloidin, and white is DAPI. Representative images from 2 biological replicates with 2 sections per replicate. Another set of images was also acquired in testing the binding of antibodies. **p**–**q** Streptavidin labeling of biotinylated proteins from streptavidin pulldown of tumor (**p**) and serum samples (**q**). Note: the stronger signal in sample 2 in **q** reflects the significantly larger tumor mass in this individual. Representative results from six (**p**) and four (**q**) western blots. For each image, all lanes are from the same blot (see Source Data for uncropped western blots). See also: Supplementary Figs. 12 and 13. Source data are provided as a Source Data file.

To show the applicability of this approach in vivo, we injected 1–2 × 10^6^ BirA*G3-ER (negative control without *Cre*) or BirA*G3-ER;Cre mESCs under the kidney capsule to generate teratomas^30,31^. Four weeks post transplantation, biotin was administered at 2000 ppm in the chow and 5 mM in the water for the final 7 days before tissue collection (Fig. 6a). Teratomas were evaluated histologically (hematoxylin and eosin staining) for an expected multi-germ layer complex tissue morphology characteristic to this class of pluripotent cell-derived tumor (Fig. 6b, c and Supplementary Fig. 12a–j). Immunostaining of teratomas showed the expected outcomes: GFP was restricted to BirA*G3-ER mESC-derived tumors, and mKate2 and BirA exclusively to BirA*G3-ER;Cre mESC-derived tumors (Fig. 6d–o and Supplementary Fig. 12k–z). BirA*G3-ER-mKate2 expression co-localized with streptavidin in BirA*G3-ER;Cre teratomas suggesting Cre-dependent biotinylation (Supplementary Fig. 12s–z). Western blotting of tumor tissues demonstrated Cre-dependent biotinylation at the total protein level (Supplementary Fig. 13a) and after streptavidin pulldown (Fig. 6p). Consistent with the secretion of biotinylated proteins from the tumors, the streptavidin pulldown of serum detected a strong biotinylated protein signature specifically in animals with BirA*G3-ER;Cre teratomas (Fig. 6q and Supplementary Fig. 13b). As expected, BirA*G3 with ER retention signal was detected in teratomas but not in serum (Supplementary Fig. 13c, d).

**Fig. 7:**
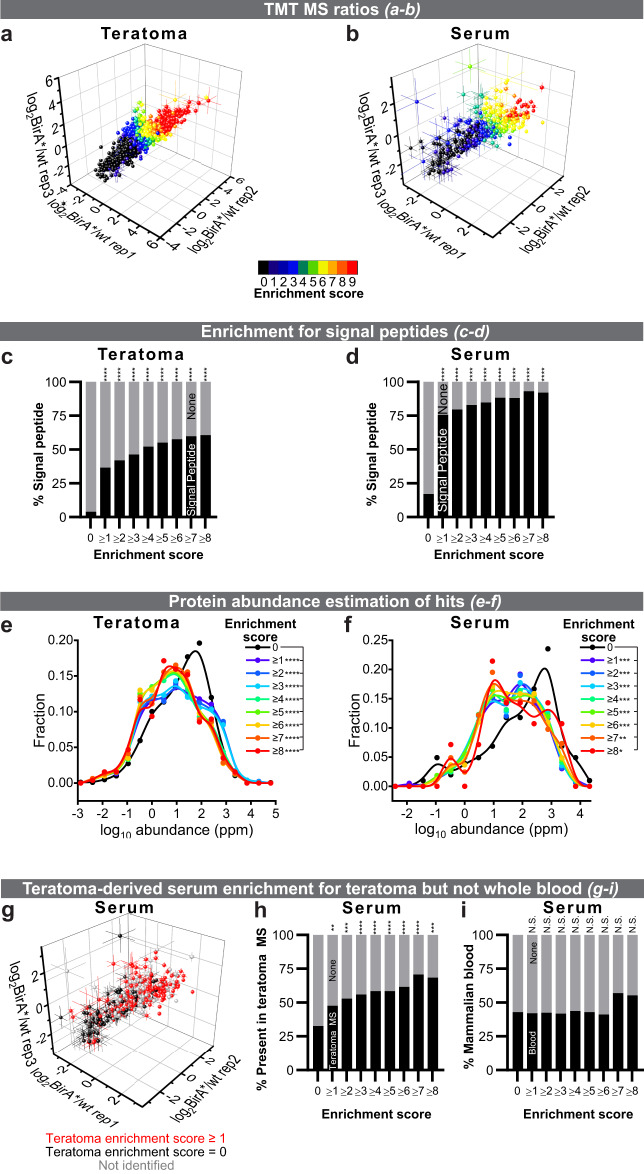
Identification of mouse teratoma-derived proteins in serum. **a**, **b** Teratoma and serum log_2_(BirA*G3-ER/wt) TMT ratios in three replicates. Each point is *n* = 3 comparisons, mean ± SEM log_2_TMT ratio. The points are colored by the enrichment score (E-S): the number of comparisons (from 9) in which TMT-ratio > threshold (score 9 is for most confident hits [red] and 0 is background [black]). **c**, **d** As the E-S increases, the fraction of proteins with putative signal peptides increases. Two-sided Fisher’s exact test. Teratoma *p*-values (****) from left to right (**c**): 5.11 · 10^−198^, 7.86 · 10^−221^, 1.03 · 10^−232^, 5.11 · 10^−241^, 2.05 · 10^−230^, 1.66 · 10^−216^, 4.73 · 10^−194^, 3.10 · 10^−167^. Serum *p*-values (****) from left to right (**d**): 1.00 · 10^−30^, 3.61 · 10^−33^, 1.82 · 10^−35^, 2.48 · 10^−35^, 1.23 · 10^−36^, 1.37 · 10^−31^, 1.63 · 10^−24^, 8.42 · 10^−18^. **e**, **f** BirA*G3-ER;Cre teratoma and serum hits were enriched for lower-abundance proteins. Protein abundance information was from an integrated entire organism PAX database for mouse^67^. Frequency vs log_10_ protein abundance plots. For representation, serum and teratoma data are shown as histograms with equal bin sizes with B-spline smooth fits (calculated using OriginPro 2020). Statistics were performed on original data. Kruskal–Wallis test and Benjamini, Krieger, Yekutieli Linear Two-Stage Step-Up FDR (two-tailed *p*-value). Teratoma *p*-values (****) from top to bottom (**e**): 0.000071, 0.000025, 0.000009, 6.72 · 10^−12^, 4.26 · 10^−11^, 4.26 · 10^−11^, 1.02 · 10^−8^, 6.79 · 10^−7^. Serum *p*-values (****) from top to bottom (**f**): 0.0004, 0.0005, 0.0005, 0.0005, 0.0005, 0.0009, 0.0041, 0.0204. **g**–**i** As the E-S increases, the fraction of serum hits that were identified in the teratoma increases (**g**–**h** colored as red in **g**). Proteins with increased scores are not enriched for proteins found in previous whole blood (cells removed) proteomics (**i**) (see “Methods” section). Two-sided Fisher’s exact test. *p*-values from left to right (**h**): 0.0044, 0.0002, 0.000025, 0.000005, 0.000012, 0.000005, 0.000001, 0.0001. *p*-values from left to right (**i**; N.S. means non-significant): 0.9161, 0.9136, 0.8243, 0.9093, >0.9999, 0.7987, 0.0850, 0.2001. See also: Supplementary Figs. 14 and 15a–n, Supplementary Table 9, Supplementary Data 4–6. Source data are provided as a Source Data file.

To evaluate and quantify biotinylated proteins in teratomas and serum samples further, we used BirA*G3-ER and LC–MS/MS with TMT (Supplementary Fig. S14a, b; Supplementary Data 4 and 5). Using the analysis pipeline similar to the one developed in the *Drosophila* experiments, we calculated the E-S for each teratoma or serum protein (details in Supplementary Fig. 14c–f; see “Methods” section). Using an E-S of at least 1, we detected 1641 labeled proteins present in the tumor and 291 in serum samples, including known hormones, growth factors, cytokines, and proteins with poorly-characterized functions (Supplementary Data 6). Proteins with higher average TMT ratios have higher E-S, highest confidence hits have E-S of 9, background proteins (non-hits) have E-S of 0 (Fig. 7a, b; Supplementary Fig. 14g, h), and as expected, hits are enriched for PC proteins (Supplementary Fig. 14i, j). The teratoma or serum biological replicates show good agreement in TMT-ratio signals (Fig. 7a, b). As expected and in agreement with the western blot data (Supplementary Fig. 13c, d), we identified 53 unique peptides for the BirA*G3 enzyme itself in the BirA*G3-ER;Cre teratomas (total intensity = 5.56 × 10^12^, average log_2_TMT ratio = 2.17 ± 0.09, and score = 9), but none in the serum (Supplementary Data 4 and 5).

Our analysis of the teratoma proteome revealed that a higher E-S correlated significantly with more signal peptide-containing, ER-resident, and transmembrane proteins, suggesting that many of our hits are ER proteins (Supplementary Fig. 14k, m–o; Fig. 7c; see “Methods” section). Specifically, of the top 381 ranked predictions with an E-S of at least 8, 61% have a signal peptide, 36% are ER-resident, and 38% have transmembrane domains. In the teratoma-secreted proteome of the serum, a higher E-S correlated significantly with more signal peptide-containing proteins, suggesting that our hits are secreted (Supplementary Fig. 14l; Fig. 7d; see “Methods” section). Of the top 38 ranked predictions with an E-S of at least 8, 92% have a signal peptide. As expected, known ER-resident proteins were not enriched in labeled serum proteins (Supplementary Fig. 14n; see “Methods” section). We estimated protein abundances of the teratoma and serum candidates using the PAX database (see “Methods” section), and determined that increased E-S showed enrichment for lower whole-body abundance proteins (Fig. 7e, f). As with the *Drosophila* data (Supplementary Fig. 5c–f), these results are consistent with the enrichment of the most relevant proteins away from abundant contaminants using biotinylation by BirA*G3.

We next compared the teratoma and serum proteomics data sets. We first showed that the top expressed (average reading cutoff = 0.5) control unlabeled background proteins from teratoma and serum show no significant enrichment for UniProt secretion annotation (Supplementary Fig. 15a-b). The overlap between control serum and teratoma does not include annotated secreted proteins, and may instead include the most abundant background proteins in both samples that non-specifically bind to streptavidin beads in the enrichment protocol (Supplementary Fig. 15c). By contrast, BirA*G3-ER;Cre-biotinylated serum, and teratoma hits are enriched for secreted proteins and show a highly statistically significant overlap with each other (*p* = 3.78 · 10^−21^; Supplementary Fig. 15d–f). In addition, as the E-S increases, the fraction of teratoma hits that were identified in the serum increases; however, not all teratoma hits were identified in the serum, suggesting that only a fraction of proteins are secreted, or the steady-state levels of some secreted proteins in the serum are below current levels of detection (Supplementary Fig. 15g, h). Moreover, as the E-S increases, the fraction of serum hits that were identified in the teratoma increases (Fig. 7g, h). Comparing the teratoma serum proteome with that of previously-published whole serum plasma (see “Methods” section) showed a distinct profile (Fig. 7i). Thus, the serum data set is enriched for proteins present in the teratoma but not those generally present in the blood.

Further analysis of teratoma streptavidin-enriched ER proteins and non-enriched proteins revealed lineage markers for mesoderm, endoderm, and ectoderm (Supplementary Table 9; see “Methods” section). Tumors show strong muscle phenotypes histologically, and consistently, analysis of the highest confidence hits in serum showed enrichment for myocyte, as well as adipocyte, secretomes (Supplementary Fig. 15i, j; see “Methods” section). Finally, using a complementary *p*-value-based approach for identifying hits (Supplementary Data 4 and 5; Supplementary Fig. 15k, l) showed a strong correlation with our E-S/thresholding-based method (Supplementary Fig. 15m, n).

**Fig. 8:**
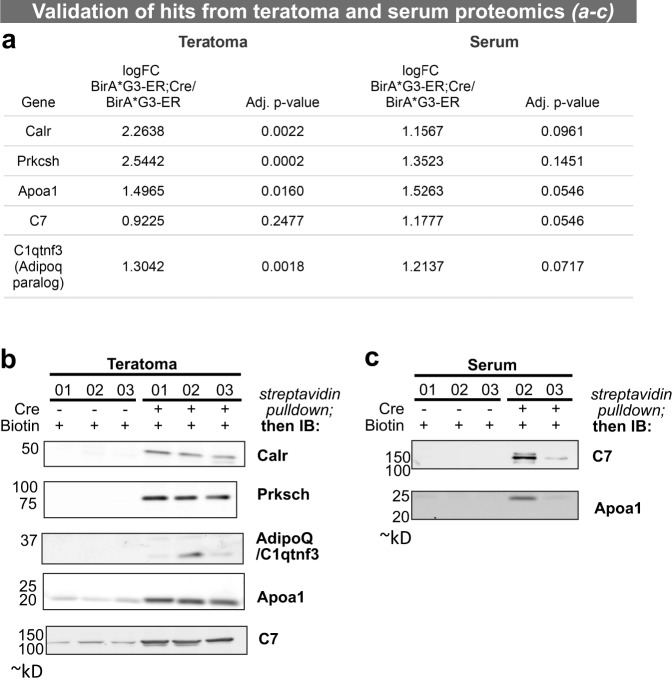
Validation of mass spectrometry proteomics-based predictions in tumor and serum samples. **a** LC–MS/MS log fold change (FC) of multimedian normalized data of BirA*G3-ER;Cre samples over BirA*G3-ER samples with adjusted (Adj.) *p*-value from two-sided two-sample t-tests for selected targets. Note: Adipoq has only 1 unique peptide from the MS data. **b**, **c** Western blot analysis of target hits in streptavidin pulldown of tumor lysates (**b**) and serum samples (**c**). No serum was available from sample 01 for the BirA*G3-ER;Cre analysis in **c**. Each slice is from a different blot, all lanes on a slice are from the same western blot (see Source Data for uncropped blots). Serum Apoa1 western blot was done twice (Supplementary Fig. 15o), while other western blots were done once, but using three (**b**) or two (**c**) biological replicates. IB means immunoblot. See also: Supplementary Fig. 15o. Source data are provided as a Source Data file.

Using streptavidin pulldowns and western blotting, proteins observed to be highly enriched MS hits in BirA*G3-ER;Cre teratoma and serum were validated based on high expression and antibody availability. Multimedian mouse log_2_BirA*/*wt* ratios were used to find the most enriched proteins in serum of BirA*G3-ER;Cre over BirA*G3-ER (control) samples. Apolipoprotein A1 (Apoa1) and complement component 7 (C7) showed log_2_BirA*/*wt* ratios greater than 1.0 and adjusted *p*-value < 0.06. Western blots of streptavidin pulldowns of serum samples demonstrated specific enrichment in serum from BirA*G3-ER;Cre tumor harboring mice validating MS predictions (Fig. 8 and Supplementary Fig. 15o). For predicted teratoma hits, the ER proteins glucosidase sub-unit beta and calreticulin were present only in BirA*G3-ER;Cre streptavidin pulldowns, and Apoa1, C7, and complement C1q tumor necrosis factor-related protein 3 (C1qtnf3; an adiponectin paralog) were all significantly enriched in BirA*G3-ER;Cre streptavidin pulldowns (Fig. 8b). Lower Apoa1 and C7 in the BirA*G3-ER streptavidin pulldowns represents the non-specific binding of these abundant serum proteins to streptavidin beads as western blot analysis shows streptavidin (biotinylation) and Apoa1 or C7 co-localization only in BirA*G3-ER;Cre streptavidin pulldowns (Source Data—Fig. 8b).

## Discussion

We have developed and applied a platform, using promiscuous BirA*, to investigate secreted protein trafficking from subcellular-compartments of specific cells to distal organs in vivo (Fig. 2a, b). In *Drosophila*, through biotinylation of ER proteins in IPCs (Dilp2), FB, and muscle we demonstrated labeling specificity and sensitivity, long-term monitoring, and wide-applicability of the method. Our platform provides biochemical evidence suggestive of proteins trafficking to other organs or distal body parts and allows differentiation between ER- and non-conventionally-secreted proteins through the use of ER- or cytosolically localized BirA*, respectively. Interestingly, our results indicate that the interorgan communication network of secreted proteins is more extensive than previously thought. We provide a resource for conserved secreted proteins that are candidate adipokines targeting legs and myokines targeting heads. We used our resource to demonstrate that the FB-secreted factor CG2145 (ENDOU) remotely binds with a specific pattern to the muscle organ (near muscles/neurons), but not to other organs. FB-specific, but not a muscle-specific reduction of levels of CG2145 adversely affects muscle function without affecting FB lipids. We also identified additional candidate FB factors—CG4332 (human ortholog: CLPTM1L (cleft-lip and palate-associated transmembrane protein 1-like)) and CG31326 (PAMR1 (peptidase domain-containing associated with muscle regeneration-1), FVII)—for which reduction in levels in FB but not in muscles decreases climbing-ability and increases protein aggregate formation in muscles (Fig. 3a–g; Supplementary Fig. 9a–g, i–l, o–r, t–x (adult-specific RNAi shown in Supplementary Fig. 9w); Supplementary Table 8; Supplementary Movies 2 and 3). Further characterization of our identified putative adipokines in *Drosophila* and mammalian models may yield important insights into systemic physiology.

In interpreting these results, one must exercise caution as the repertoire of identified proteins may include those present in the hemolymph, as opposed to those present specifically within the muscle organ or heads. Supportive of binding to target organs, we observed proteins known to be involved in interorgan communication pathways, including a number that have known receptors and/or previously shown to interact with muscle organ in specific patterns, as well as FB-secreted proteins in hemolymph-free buffer-washed dissected brains. In addition, while this may be due to detection limit differences, 60% of our FB-derived proteins identified in legs have not been observed in our hemolymph MS (Supplementary Fig. 4i), and not all FB-derived hemolymph or signal peptide-containing proteins were identified in the FB-to-legs proteomics data set, suggesting that only some secreted FB proteins traffic to muscles (Supplementary Fig. 16a–c). Further tests are required to determine whether the FB-derived factor(s) of interest affect muscle function through a direct mechanism. Using CG2145, we demonstrated direct and specific binding to the muscle organ (near muscle/neurons within muscle fields), suggesting specific receptor-like interactions and remote action in thoracic muscle organs by FB-produced proteins.

To transition the BirA* approach to mammals, we generated BirA*G3-ER-expressing kidney teratomas and identified several known hormones or signaling proteins in the teratoma-derived serum proteomics (Supplementary Data 6). Although the amount of their production from the teratoma is unclear, these factors have been reported to circulate in *wt *serum on the order of ng/mL (Prl2c, resistin, insulin growth factor 2, angiopoietin, C1qtnf3, Postn, IGFBPs, PPBP), and pg/mL (colony-stimulating factor 1; Supplementary Data 6). In our mouse serum, we identified proteins previously found in whole mammalian blood (see “Methods” section), however, only some serum proteins were biotinylated, suggesting of specificity to teratoma-labeling (Supplementary Fig. 16d). For example, albumin, the most abundant protein in whole blood, was not significantly enriched in teratoma-derived biotinylated serum proteome (average log_2_TMT ratio = −0.51 ± 0.17 for serum and −0.27 ± 0.13 for teratoma).

It is possible that biotinylation of proteins in the ER may affect the modification and sorting properties of labeled proteins. Future studies may also systematically examine whether the secretome of a cell type under study is affected by biotinylation. Although we do not observe obvious phenotypes in the BirA* flies and leakage of BirA* to the circulation, it is possible that biotinylation could lead to ER stress or modify the localization or activity of signaling components, though in the fly data several controls show an expected distribution (e.g., Tig and fon). Characterizing potential ER stress due to the BirA* system in mice is potentially confounded by teratoma-to-teratoma variability and diseased states (e.g., hypoxia) inherent to teratomas. Future studies of BirA* activity in normal mouse tissues will provide additional insight and facilitate the development of labeling strategies to minimize unwanted outcomes (e.g., by shortening the labeling time window).

Our platform is widely applicable to research in interorgan, local (within a tissue), or intracellular (e.g., within neuronal projections) protein trafficking, unconventional secretion, protein trafficking in co-culture systems, and to mouse or other organisms in vivo. For example, with the proof-of-principle tumor studies here, we expect mouse strains enabling Cre-dependent biotinylation in the secretory pathway in the organism to enable insights into cell/tissue/organ function and interaction under healthy or diseased conditions. In addition, transiently-secreted proteins or those released due to perturbations (such as diet, stress, tumors)^1^ or circadian rhythms may be studied by inducing BirA* biotinylation using biotin for a limited time. This may be achieved using time-restricted feeding, systemic injections, or local delivery of biotin. Finally, we note that during the final editorial revisions of our manuscript, two additional reports appeared in press reporting the use of protein biotinylation to study protein secretion^32,33^, underscoring the interest of the approach.

## Methods

### *Drosophila* stocks

The fly stocks and genotypes used in this study are summarized in Supplementary Table 1. BirA*R118G was generated by mutating R118 to G in the pDisplay-BirA-ER (endoplasmic reticulum) (IgK signal peptide-HA-BirA-KDEL; Addgene 20856)^34^ using QuikChange mutagenesis kit (Agilent). For the improved, highly-active BirA*G3-ER, a BiP signal peptide (for targeting to ER^35^) and KDEL were added using PCR primers (Supplementary Table 2). Constructs were cloned using Gateway methods^36^ (ThermoFisher Scientific) into *pENTR/D-TOPO* (Thermo Scientific), and *pWalium10roe* vector was used as a final destination vector^37^. The constructs were injected into *nanos-ΦC31-integrase* expressing embryos into *attP40* or *attP2* sites, as previously described^37^. In Supplementary Fig. 1j, hemolymph from *wt* flies of *white* background was used (specifically *tubGal80ts,Esg-Gal4,UAS-GFP/+(w[1118])*^38^*)*.

### *Drosophila* culture

Flies were cultured using standard methods using Perrimon lab food^39^. Preliminary experiments using BirA*R118G-ER showed that maximum BirA*R118G-ER expression and activity occurs at 30–31 °C and with 2 copies of BirA*R118G-ER together with 2 copies of *MHC-Gal4* (BirA*R118G-ER(attP40)/UAS-BirA*R118G-ER(attP40);MHC-Gal4/MHC-Gal4), or 3 copies of BirA*R118G-ER and 1 copy of *LPP-Gal4* (UAS-BirA*R118G-ER(attP40)/UAS-BirA*R118G-ER(attP40);LPP-Gal4/UAS-BirA*R118G-ER(attP2)). For BirA*R118G or BirA*R118G-ER, crosses were performed at 22–25 °C. After collection, adults were moved to 30–31 °C for 4 days (with food changes every 2 days). Adults were subsequently transferred to 50 µM biotin food (see below for preparation) at 30–31 °C for 6 days (with food changes every 2 days) until processing/collection. For BirA*G3 or BirA*G3-ER, crosses were performed at 25 °C. After eclosion and collection at 22 °C, adults were moved to 29 °C for 4 days (with food changes every 2 days). Adults were subsequently transferred to 50 µM biotin food (see Biotin-containing fly food preparation) at 29 °C for 6 days (with food changes every 2 days) until processing/collection. For RNAi experiments, crosses were performed at 25 °C. After eclosion and collection, adults were transferred to 27 °C (TRiP and NIG stocks) or 29 °C (VDRC stocks), and were regularly transferred to fresh food until experiments. For temperature-sensitive experiments, crosses were performed at 18 °C, and flies were shifted to 29 °C as adults (Supplementary Fig. 9w).

### Biotin-containing fly food preparation

Biotin stock solution (around 18 mM) was prepared from solid biotin (Sigma B4639) in water, with final pH adjusted to around 7.2, and stored at −20 °C. To prepare fly food containing 50 µM biotin, regular lab fly food was boiled three times until consistently liquid and the temperature was 100 °C. The food was then weighed in an empty beaker, and the volume was calculated using the approximate food density of 1.04 g/mL. Next, the food was cooled down to around 60–65 °C and a final concentration of 50 µM biotin was added. The mixture was then stirred well using a blender, and poured into vials or bottles (enough to cover the surface). Finally, the food was dried for several hours overnight under cheesecloth, bottles or vials were plugged, and stored at 4 °C until use.

This concentration (50 µM) of biotin was chosen based on published in vitro data with BirA*R118G^10^, on our preliminary in vitro experiments with BirA*R118G and BirA*R118G-ER, and on our preliminary experiments in vivo, where higher concentrations of biotin (500 µM) did not cause an increase in biotin labeling over 50 µM based on experiments with LPP>BirA*R118G, Dmef2>BirA*R118G, *MHC*>BirA*R118G, ACT(actin)-Gal4>BirA*R118G, or CG(FB driver)-Gal4>BirA*R118G.

### Lifespan or survival analysis

Lifespan analysis was performed as part of this study^26^. Flies were raised on normal food without biotin, and after eclosion and collection, adults (males and females separately) were transferred at 29 °C to normal food (no biotin) or to food containing 50 µM biotin for the remainder of their lives. Flies were transferred to fresh food regularly, and lethality was counted.

### Preparation of *Drosophila* body parts, tissues, and dissections

#### Preparation of body parts for MS

Established methods^40–42^ were used to prepare *Drosophila* body parts and tissues, with modifications. Fly whole bodies were flash-frozen in liquid nitrogen by transferring flies to a 15 mL centrifuge tube using a funnel (no CO_2_), and stored at −80 °C until use. During head and leg preparation, flies were kept on dry ice as much as possible, and body parts were prepared in small batches. A sieve assembly was constructed, consisting of a #25 710 µm metal sieve on top, #45 355 µm metal sieve in the middle, and 3 mm chromatography paper on the bottom (Whatman 3030-917). Note that for legs, our preliminary experiments using *LPP-Gal4>UAS-GFP* suggested that leg femur, tibia, and tarsus (that is, below the coxa)^43,44^ do not have detectable FB or GFP expression (Supplementary Fig. 2bb). Flies were vortexed 4 times for 15 s each, with a rest on dry ice in between. Vortexed flies were then decanted onto the top of the sieve assembly (710 µm), shaken, and mixed with a brush. Bodies without heads were found on the top of the 710 µm sieve. Where appropriate, bodies were examined for the absence of other body parts, transferred to a microcentrifuge tube, processed (see below), or flash-frozen and stored at −80 °C. Further, the 355 µm sieve was shaken and mixed with a brush. Heads were found on top of the 355 µm sieve. Where appropriate, heads were examined for the absence of other body parts, transferred to a microcentrifuge tube, processed, or flash-frozen, and stored at –80 °C. Finally, on the bottom chromatography paper, legs (consisting of those parts below the coxa) were separated from other debris, transferred to a microcentrifuge tube, processed, or flash-frozen, and stored at −80 °C.

#### Organ dissections

Brains, abdomens, and thoraxes were dissected in cold phosphate buffered saline (PBS) (Invitrogen)^26,39,45^, and processed further (see below). After every group/genotype, the forceps were cleaned and the razor blade was changed and cleaned. For brains in Supplementary Fig. 3e, any FB that was loosely attached to the brain was carefully and completely removed. For western blots, thoraxes and abdomens were separated using razor blades. For immunofluorescence imaging of thoracic muscles, the head, abdomen, and wings were carefully removed using clean forceps in cold PBS. The thorax was then oriented dorsal side up (towards the viewer), and each thorax was cut down along the midline (median or midsagittal plane) to generate the left and right fragments to expose the muscles. For immunofluorescence imaging of abdominal FBs, abdomens were separated from thoraxes, cut on the ventral side to expose the FB and other internal organs, and internal organs were removed^46^.

#### Hemolymph collection

Hemolymph was collected as part of this study^47^. Less than 20 flies were processed at one time. On the CO_2_ pad, flies were punctured using a tungsten needle (Fine Science Tools). Based on the pattern of *LPP-Gal4>UAS-GFP* expression, for FB-derived proteins, we selected GFP/FB-negative regions of the dorsal thorax (middle to anterior scutum), just lateral to the midline (between the midline and the dorsocentral line defined by large dorsocentral bristles and medial end of the transverse suture)^48^. Also, based on the pattern of *MHC-Gal4>UAS-GFP* expression, for muscle-derived proteins, we selected GFP-negative regions of the dorsal abdomen (A4 yellow abdominal segment^49^), just lateral to the midline. Punctured flies were transferred to a 0.5 mL microcentrifuge tube that contained a hole (pierced with a 25 gauge needle). The tube with flies was then transferred to a 1.5 mL low-protein binding microcentrifuge tube (Eppendorf), containing 15 µL of phosphate-buffered saline (PBS). The two-tube assembly was then centrifuged for 5 min at 2348 × *g* at 4 °C. Next, the 0.5 mL microcentrifuge tube was taken out, and the hemolymph in PBS in a 1.5 mL tube was centrifuged for 5 min at 2348 × *g* at 4 °C. Further, the supernatant was collected, transferred to another 1.5 mL tube, and centrifuged at 14,000 × *g* for 15 min at 4 °C. Finally, the supernatant was collected, transferred to another 1.5 mL tube, flash-frozen, and stored at −80 °C. For hemolymph MS proteomics, a slightly modified hemolymph extraction protocol was employed: thorax-punctured flies were transferred in bulk (>20 flies per tube) to pierced 0.5 mL microcentrifuge tube and centrifuged twice for 4 min at 1503 × *g* at 4 °C. The next steps are as described above.

### Targeted ES cells

#### Generation of R26 BirA*G3-ER Mouse embryonic stem cells (ESCs)

The ES cell line B6(Cg)-Tyr<c-2J>/J (https://www.jax.org/strain/000058) was used to target the *Rosa26* locus with a Cre-inducible mouse codon-optimized BirA*G3-ER vector including the elements: CAGGS-GFP/BirA*G3-ER-myc-IRES mKate2 (R26 BirA*G3): Clones A11, A2, B1, C2, D8. The sequence of the inserted elements is available upon request from the corresponding authors.

#### Generation of Cre activated (R26 BirA*G3-ER;Cre) ES cells

R26 BirA*G3-ER Clone B1 ES cells were expanded on mitotically inactivated (Mitomycin C Sigma M0503) DR4 derived (Jackson lab Dnmt1^tm3Jae^ Hprt^b-m3^ Tg(pPWL512hyg)1Ems/J) mouse embryo fibroblasts (MEF) in 4500 mg/L Dulbecco’s Modified Eagle’s Medium (DMEM; ThermoFisher 11965-118) supplemented with 15% ES cell qualified Fetal Bovine Serum, 0.1 mM β-Mercaptoethanol, 2 mM l-Glutamine (ThermoFisher 25030-081), 0.1 mM MEM Non Essential Amino Acids (ThermoFisher 11140-050), 1 mM Sodium Pyruvate (Sigma S8636), 50 units of Penicillin, 50 µg Streptomycin (ThermoFisher 15070-063) and 2 × 10^4^ units of Leukemia Inhibitory Factor (Sigma ESG1107). Upon confluence a 6 cm dish was dispersed with trypsin and 4.7 × 10^6^ ESC were resuspended in cold PBS (Mg/Ca free) mixed with 40 µg *pCaggs-Cre* plasmid and electroporated (Biorad Gene Pulser: 240 V, 500 uFD); 2.9 × 10^6^ ES cells were plated to a 10 cm dish of MEF feeder cells. Growing colonies were examined for the loss of GFP fluorescence and the activation of mKate2. Colonies that were RFP+/GFP- were picked, dispersed and serially diluted to generate single-cell plating in 96 well MEF feeder plates. Candidate clones were expanded and monitored for RFP+/GFP- fluorescence, resulting in four R26 BirA*G3-ER;Cre clonal lines (3A, 4A, 6A, 7A). There was no evidence of integration of the *pCaggs-Cre* plasmid into the DNA of the mKate2+ clones as tested by PCR. (Note: *pCaggs-Cre* was a gift from Connie Cepko (Addgene plasmid 13775; http://n2t.net/addgene_13775).

### Teratoma generation

The procedure was adapted from Solter^30,31^. All surgeries and animal work were carried out according to federal and institutional guidelines. Animal protocols covering the work in APM’s laboratory were approved by the University of Southern California’s IACUC committee. The mouse vivaria are on a 14–10 h light-dark cycle. The lights turn on at 5 am and turn off at 7 pm. The ambient temperature is kept between 70–75 °F and the ambient humidity is 30–70%. R26 BirA*G3-ER and R26 BirA*G3-ER;Cre 3 A ES cells were grown to confluence on mitotically inactivated MEFs in 6 cm tissue culture dishes. ESCs were trypsinized, split to 1–2 × 10^6^ cells/tube, pelleted at 300 × *g*, and left on ice while the recipient mice were prepared for surgery. Prior to injection, the cell pellets were placed in 40 µL growth media or 50% Matrigel (Corning BD354277) maintaining clumps. No difference in tumor formation was observed between Cre+ and Cre- R26 BirA*G3-ER clones. Week 8–12 C57Bl/6 N male mice (Charles River) were anesthetized with Ketamine/Xylazine. The surgical site, on the dorsal flank, was shaved and wiped with Proviodine and alcohol. An 8–10 mm incision was made, the fascia was incised and the left kidney was externalized. The kidney capsule was kept moistened with sterile saline during the procedure. A small incision was made in the outer membrane of the renal capsule at the caudal end, using a sharp 24 gauge needle and the subcapsular space was flushed with 0.5–1 mL of sterile saline using a sterile, blunted 30 gauge needle (B30-50, Strategic Applications, Inc.) attached to a 1 mL syringe. A 20 or 24 gauge indwelling needle (SURFLO® PTFE I.V. Catheter needle, TESR-OX2025CA, VWR) attached to a 1 mL syringe was flushed with ES cell growth media (-LIF), and 1–2 × 10^6^ cells of the pelleted ES cells were drawn into the tip of the needle and injected into the subcapsular space. The capsule incision was briefly cauterized, and the kidney was replaced into the retroperitoneum. Subsequently, the muscle layer was sutured (Ethicon J494G Coated Vicryl Suture, e.sutures.com), and the skin was closed with wound clips.

### Teratoma treatment and collection

Mice receiving ES cell injections were maintained on regular chow and water for 3 weeks followed by a 7 day period of biotin supplementation: 2000 ppm in the chow (LabDiet, 5WLP) and 5 mM in the water (Sigma, B4639). Mice were euthanized 4 weeks post-ESC injection, urine and blood were collected, and the mice were perfused with cold PBS, prior to collection of tissue samples. The blood was allowed to clot at room temperature for 30 min, then samples were centrifuged at 2000 × *g* for 10 min at 4 °C, the serum collected and re-spun for an additional 10 min, then samples were aliquoted, flash-frozen in liquid nitrogen, and stored prior to use at −80 °C.

Tissue samples for histology or immunohistochemistry were fixed for 2 h in 4% paraformaldehyde in PBS at 4 °C, washed three times in PBS for 15 min. and then left in 30% sucrose overnight at 4 °C. The samples were transferred into OCT (Tissue-Tek* O.C.T. Compound, VWR, 25608-930), frozen in a bath of ethanol and dry ice then stored at −80 °C. Sections were cut by cryostat at a thickness of 10–12 µm onto superfrost microscope slides (VWR, 48311-703) and stored at −80 °C prior to use.

### In vitro assays

R26 BirA*G3-ER and R26 BirA*G3-ER;Cre 3 A ES cells were grown in 6 cm tissue culture plates on mitotically inactivated MEF to confluence. The cells were trypsinized, split to 0.5 × 10^6^ cells/well, and allowed to grow for 2 days before experimental treatment. Three days post-split, cells were treated with 50 μM biotin for 24 h for general characterization. For the biotin dose study, R26 BirA*G3-ER;Cre 3A ES cells were placed on feeder-free 10 cm tissue culture dishes to spontaneously differentiate to fibroblast-like cells for multiple passages. Cells were then plated onto 6 cm tissue culture at 0.5 × 10^6^ cells/well. Two days post-plating, cells were treated with biotin in varying concentrations (diluted in DMEM from a 50 mM stock solution in dH_2_O, pH 7.4): 0, 5, 10, 12.5, 25, 50, 100, or 250 μM biotin. For the biotin time exposure study, cells were plated as above and treated with 50 μM biotin for 1, 5, 10, 15, 30, 60, 120, 240, 480, or 720 min.

### Biotin-conjugated beads pulldowns

Biotin-conjugated bead pulldowns (Supplementary Fig. 1j) were performed according to the manufacturer’s instructions (RayBiotech), with modifications. Carboxyl magnetic beads (control beads; RayBiotech 801-114-2) were washed (using a magnetic stand for separation) four times in 1 mL each of tris-buffered saline (TBS; Sigma-Aldrich) and resuspended in storage buffer (0.1% bovine serum albumin (BSA) in TBS; filtered through a 0.2 µm-membrane). Next, biotin-conjugated magnetic beads (RayBiotech 801-107-1; stored in a similar buffer to the carboxyl bead storage buffer) and carboxyl beads were washed twice in 1 mL each of wash buffer (TBS with 0.05% Tween-20) and then resuspended in 485 µL of wash buffer. Then, samples were added (15 μL of hemolymph per pulldown) and mix using end-over-end mixing for 30 min at room temperature. Flow-through and hemolymph input were saved. Beads were then washed twice in 1 mL each of wash buffer, resuspended in 35 µL of ProSieve ProTrack Dual Color Protein Loading Buffer with 1 × DTT (Lonza; diluted in TBS) and boiled for 5 min and vortexed to elute bound proteins. Flow-through and hemolymph input samples were mixed with loading buffer and boiled for 5 min. Samples were run on a protein gel as described below in the Western blotting section. Gels were then stained using sensitive Flamingo staining^50^ (Biorad), according to the manufacturer’s instructions. Gels were placed in pre-cleaned (in ethanol, water, and then fixative solution [50% ddH_2_O/40% ethanol/10% acetic acid]) new empty pipette box containers and submerged in 100 mL of fixative solution for 2 h at room temperature in the dark with gentle shaking. Next, the fixative was removed, 50 mL of 1× Flamingo staining solution (diluted in ddH_2_O; Biorad 161-0490) was added, and gels were stained overnight at room temperature in the dark with gentle shaking. Destaining was not performed. Gels were imaged on the Typhoon Trio Imager (GE Healthcare Life Sciences) using the settings: blue laser (488 nm), 520BP40 emission filter, 215 or 250 V PMT, and high sensitivity.

### Protein lysate preparation

Protein lysates were prepared using established protocols, with modifications^45,51^. The lysis buffer was RIPA buffer (made in the lab, 50 mM Tris, pH = 8.0, 150 mM NaCl, 0.1% SDS, 0.5% sodium deoxycholate, 1% Triton X-100; or commercial (Pierce)) supplemented with 1 mM benzamidine hydrochloride (VWR), 4 µM pepstatin (VWR), 100 µM PMSF (Sigma-Aldrich), and one cOmplete ULTRA Mini EDTA-free protease inhibitor tablet (Roche). Tissues in lysis buffer were combined with zirconium oxide beads (NextAdvance) and homogenized several times for 4 min on setting 9 using the Bullet Blender (NextAdvance), with additions of extra lysis buffer where necessary, and brief centrifugations in between. Additional lysis buffer was added where necessary, and samples were left on ice for 30 min. Next, samples were centrifuged for 15 min at 16,000 × *g* at 4 °C, and supernatants were transferred to low-protein binding tubes (Eppendorf). Total protein concentrations were measured using the BCA protein assay kit (Pierce), according to the manufacturer’s instructions. Protein samples were diluted to equal concentrations in each experiment using lysis buffer. Protein lysates were flash-frozen and stored at −80 °C.

Mouse embryonic stem cells (mESC) or mESC-derived fibroblasts were collected in 1 mL PBS via scraping and then pelleted at 300 × *g* for 5 min. Pellets were resuspended in 40 μL lysis buffer (M-PER (ThermoFisher, 78501) with 1× protease inhibitor cocktail (CellSignaling, 5871S)). Cells were lysed at room temperature for 10 min and tapped to shake at the 5 min mark. Teratomas were homogenized in 500 μL RIPA complete lysis buffer (RIPA buffer (ThermoFisher, 89901) with 1× cOmplete mini EDTA-free protease inhibitor cocktail (Sigma, 11836170001), 1 mM benzamidine hydrochloride (VWR, TCB0013-100G), 4 μM pepstatin (Sigma, EI10), 100 μM PMSF (Sigma, 11359061001)) and bead homogenized using stainless steel beads (NextAdvance, SSB14B-RNA) for 5 min at setting 10, Bullet Blender Storm (NextAdvance, BT24M). Both cell and tissue lysed samples were centrifuged at 14,000 × *g* for 15 min at 4 °C to remove cellular debris and the supernatant was collected. Protein lysates were quantified by Pierce BCA (ThermoFisher, 23227) microplate assay or QuBit 2.0 Fluorometer Protein assay (ThermoFisher, 33211). Protein lysates were flash-frozen and stored at −80 °C. For the analysis of Alpl secretion, media (serum-free) from cell cultures were collected and flash-frozen in liquid nitrogen until needed. Centrifugal filter units (MilliporeSigma UFC503096, 30 kDa cutoff) were used to concentrate protein from media per manufacturer’s instructions (500 μL media input, 30 min spin, 14,000 × *g*). Concentrated media (1–2 μL/lane) was then used for western blot analysis.

### Streptavidin beads pulldowns

Streptavidin pulldowns were performed as part of this study^51^. Streptavidin magnetic beads (Pierce 88817) were washed (using a magnetic stand for separation) twice in lysis buffer (see above) and resuspended in lysis buffer. For pulldown-western blot experiments in Fig. 1e and Supplementary Fig. 3a, 64 µg of protein was combined with 32 µL of beads. In Supplementary Fig. 3b, 140 µg of protein (1.42 µg/µL in lysis buffer) was combined with 40 µL of beads. In Supplementary Fig. 3e, 36 µg of protein (0.25 µg/µL) was combined with 18 µL of beads. For *Drosophila* MS experiments, 4.8 mg (BirA*R118G-ER, at 3.98 mg/mL) or 8.5 mg (BirA*G3-ER, at 12.2 mg/mL) of total protein per sample was combined with 0.6 mL of beads. For mouse teratomas, 9 mg of total protein per sample was combined with 0.9 mL of beads. For mouse serum, 2.5 mg of total protein per sample was combined with 0.25 mL of beads. Samples were incubated overnight at 4 °C with end-over-end mixing. On the next morning, beads were washed twice in lysis buffer, once in 2 M urea in 10 mM Tris (pH = 8.0), and twice in lysis buffer. For mouse sample western blots, streptavidin magnetic beads (Pierce, 88817) were washed once and resuspended in RIPA complete lysis buffer (above). Pulldown reactions were set up as follows: 400 μL RIPA complete lysis buffer, 10 μL beads/100 μg protein. Pulldown reactions either used 100 or 200 μg of input protein. Reactions were incubated at 4 °C overnight, rotating. The following day, beads were washed on a magnetic rack twice with 500 μL of RIPA complete lysis buffer, followed by 500 μL 2 M Urea in 10 mM Tris, and twice again with 500 μL RIPA complete lysis buffer. Beads were resuspended in 12 μL Li-Cor loading buffer (Li-Cor, 928-40004) with 1.43 M β-mercaptoethanol. See the “Western blotting” and “Quantitative mass spectrometry” sections for further bead processing.

### Western blotting

Western blots were performed according to established protocols, with modifications^51,52^. Equal amounts of total protein were mixed with 1× loading buffer (62.5 mM Tris pH = 6.8, 2% sodium dodecyl sulfate, 10% glycerol, 0.025% bromophenol blue, 100 mM DTT; or ProSieve ProTrack Dual Color Protein Loading Buffer, Lonza) and boiled for 5 min. To elute bound biotinylated proteins in streptavidin pulldown experiments (see above), washed beads were resuspended in equal volumes of 1× loading buffer (diluted in lysis buffer), boiled for 5 min, and vortexed. Equal amounts of sample were loaded to a PAGEr EX gel (Lonza) and ran using ProSieve EX Running Buffer (Lonza) according to the manufacturer’s instructions. Spectra Multicolor Broad Range Protein Ladder (Pierce) was used as a molecular weight marker. The samples on the gel were transferred to a nitrocellulose membrane using ProSieve EX transfer buffer (Lonza) for 20 min, according to the manufacturer’s instructions. The membrane was then rinsed 3 times in deionized water, followed by 3 times for 5 min each in Tris-buffered saline (Sigma-Aldrich) with 0.1% Tween-20 (TBST) with gentle shaking.

For streptavidin western blots, the membrane was incubated in blocking buffer (5% dialyzed BSA, diluted in TBST) overnight at 4 °C with gentle shaking. Next, the membrane was incubated for 1 h at room temperature in 1:40,000 streptavidin-HRP (Invitrogen) in a blocking buffer. Subsequently, the membrane was washed three times quickly in TBST, followed by 6 times, 5 min each in TBST with gentle shaking. Detection was performed using ECL hyperfilm (GE Healthcare), first using the ECL substrate (Pierce), then by thrice washing in deionized water and twice for 5 min each time in TBST, followed by the Supersignal West Pico substrate (Pierce).

For other western blots, the membrane was blocked in 5% milk in TBST for 1 h at room temperature with gentle shaking. Next, the membrane was incubated in a primary antibody dilution in 5% milk in TBST overnight at 4 °C with gentle shaking. Primary antibodies were used at the following concentrations: 1:1000 mouse anti-myc (clone 9E10, Santa Cruz Biotechnology), 1:2000 rat anti-HA (clone 3F10, Roche), 1:1000 goat anti-Alpl (AF2910, R&D systems), 1:10,000 mouse anti-tubulin (clone B-5-1-2, Sigma-Aldrich; incubation can also be done for 1 h at room temperature). Further, the membrane was washed three times quickly in TBST, followed by 6 times, 5 min each in TBST with gentle shaking. The membrane was then blocked in 3% milk in TBST for 1 h at room temperature with gentle shaking and incubated for 1–2 h at room temperature in 3% milk in TBST with 1:5000 to 1:10,000 dilution of HRP-conjugated secondary antibody: sheep anti-mouse (GE Healthcare), donkey anti-mouse (Jackson ImmunoResearch), or goat anti-rat (GE Healthcare). For tubulin western blot, a 1:10,000 secondary antibody dilution was used. Detection was performed using ECL hyperfilm (GE Healthcare), first using the ECL substrate (Pierce), followed by the Supersignal West Pico substrate (Pierce), and by Supersignal West Femto substrate (Pierce). Where necessary, the membrane was stripped using the Restore Plus Western Blot Stripping Buffer (Pierce) for 15 min at room temperature with gentle shaking, followed by thrice washing in deionized water, and 6 times, 5 min each washing in TBST. In Supplementary Fig. 13c, d, a Biorad Chemidoc MP imager was used to acquire the images.

Fig. 1d is a representative result of 3 western blots, and Fig. 1d′ is the quantification of the integrated density of myc signals (background-subtracted), normalized to Tub staining integrated density (background-subtracted), performed using established methods^53^. Specifically, the integrated density of each band was measured using Adobe Photoshop CC2019 on inverted 8-bit grayscale images, using a 64 × 30 pixel box. The integrated density of the background above each band was subtracted from each band’s integrated density value. The background-subtracted myc values were then normalized to Tub background-subtracted values for each sample. Note that the thoracic BirA*G3-ER-myc is consistently detected in 3 western blots and using confocal microscopy in the thoracic muscles (Supplementary Fig. 2cc–jj). Also, myc is not detected in LPP>BirA*G3-ER legs, as is indicated by the quantification (Fig. 1d′). Fig. 1e is a representative result of 3 western blots, including the repeat shown in Supplementary Fig. 3a. In Fig. 1d, e and Supplementary Fig. 3a males and females were used, and *w[1118]* flies were used as *wt* control.

#### Western Blot (fluorescent) analysis

Following pulldowns, beads were resuspended in 12 μL Li-Cor loading buffer (Li-Cor, 928-40004) with 1.43 M β-mercaptoethanol. Beads were boiled at 95 °C for 5 min to elute proteins. Equal amounts of total protein were mixed with 1× Li-Cor loading buffer (above) and boiled at 95 °C for 5 min. Samples were briefly spun down and placed on ice before loading. Total protein or pulldown protein elutes were loaded on 10% SDS acrylamide gels and ran using standard 1× SDS-Running buffer with 4 μL of Li-Cor one-color molecular marker (Li-Cor, 928-40000). Samples on the gel were transferred to methanol-activated PVDF 0.45 μm membranes using Biorad’s wet tank mini-protean system for 1–3 h at 300 constant mA in a sample-dependent context. After transfer, membranes were dried at 37 °C for 5 min and then re-activated with methanol. Blots were stained with Li-Cor’s Revert-700 Total Protein Stain (Li-Cor, 926-11010) for normalization and imaged using a Li-Cor Odyssey Clx. Blots were then de-stained per kit instructions and put in block (Li-Cor Intercept block, 927-60001) for 1 h at room temperature with shaking. Blots were then transferred to primary antibody (block with 0.1% Tween-20) and incubated overnight at 4 °C with shaking. Primary antibodies: Rb Adiponectin (Abcam, ab181699, 1:1000), Rb C7 (Abcam, ab126786, 1:1000), Rb Apoa1 (Abcam, ab20453, 1:1000), Rb Glucosidase sub-unit beta (Abcam, ab134071, 1:1000), Rb Calr (Abcam, ab92516, 1:1000), Rb myc-tag (Abcam, ab9106, 1:1000), Gt Alpl (mouse alkaline phosphatase; R&D systems, AF2910, 1:1000). The following day, blots were washed four times in TBST for 5 min each at room temperature, shaking, and then incubated in secondary antibody (1:10,000) in block with 0.1% Tween-20 and 0.1% SDS, and/or streptavidin conjugate (1:5000; 680 or 800, Li-Cor, 926-68079, 926-32230) if visualizing biotinylated proteins, for 1 h at room temperature with shaking. Blots were then washed twice with TBST for 5 min each at room temperature with shaking, followed by two 5-min TBS washes at room temperature with shaking. Blots were imaged on a Li-Cor Odyssey Clx. After imaging blots were dried at 37 °C for 5 min, then stored.

#### Western blot (fluorescent) quantification

Biotinylation levels were quantified via western blot using Li-Cor’s fluorescent western blot Emperia Studio (Version 1.3.0.83) and ImageStudio (Version 5.2.5) analysis software and protocols. Total protein stain images of each blot were used to normalize biotinylation (streptavidin) signal intensity in R by determining the lane normalization factor (Li-Cor) for each blot, which was then used to normalize protein of interest signals. ggplot2 and GraphPad Prism 8.0 were used to visualize normalized biotinylated protein signal.

### Spatially informed, one-antibody bead enzyme-linked immunosorbent assay (ELISA)

By modifying standard ELISA methods^11,54^, we developed a spatially informed, one-antibody bead ELISA protocol for biotinylated Dilp2-HA-Flag (schematic in Supplementary Fig. 3q). Protein lysates from heads and bodies from males and females were prepared as described above (Preparation of *Drosophila* body parts, tissues, and dissections and Protein lysate preparation sections). Standard Flag-GS-HA peptide (DYKDDDDKGGGGSYPYDVPDYA-NH_2_, molecular weight 2411.45 Da, LifeTein)^11^ was dissolved in water, aliquoted, and stored at −80 °C. Note that the standard peptide concentration calculation took into account synthesized peptide purity. Flag-GS-HA peptide biotinylation was performed with EZ-link sufo-NHS-biotin (Thermo Scientific), according to the manufacturer’s instructions. Flag-GS-HA (final concentration of 2.5 mg/mL) or water background control was combined with 5-, 20-, or 50-fold molar excess of sulfo-NHS-biotin in a total reaction volume of 6 µL for at least 2 h on ice. For the standard peptide in Fig. 1c, we used a 20-fold excess of sulfo-NHS-biotin. Biotinylated Flag-GS-HA was stored at −80 °C or used immediately. Biotinylated Flag-GS-HA or background control dilutions for ELISA were performed in fetal bovine serum (FBS, heat-inactivated, Gibco).

We coupled antibodies to tosylactivated Dynabeads M-280 (Invitrogen), based on the manufacturer’s instructions. Beads were washed twice in 500 µL of 0.1 M borate buffer, pH = 9.5 (using a magnetic stand for separation) in a 0.5 mL low-protein binding tube (Eppendorf). Next, 1 mg of beads, 20 µL of borate buffer, 40 µL of 3 M ammonium sulfate in borate buffer, and 40 µL of 0.5 mg/mL of antibody were combined (these amounts were scaled as needed), and incubated end-over-end at 37 °C overnight. The antibodies used were: rat anti-HA (clone 3F10, Roche) and mouse anti-myc (clone 9E10, Santa Cruz Biotechnology). For the anti-myc antibody, the original concentration was 0.2 mg/mL, and it was concentrated to approximately 0.5 mg/mL using a 30 kD cutoff centrifugation concentration device (Amicon, EMD Millipore). Alternatively, 1 mg of beads, 65.88 µL of 3 M ammonium sulfate in borate buffer, and 100 µL of 0.2 mg/mL antibody solution were combined, and incubated end-over-end at 37 °C overnight. For biotinylated Dilp2-HA-Flag detection, we chose to not use anti-flag as the capture antibody because the flag tag (DYKDDDDK)^55^ has a number of lysine groups that may be modified by biotin, potentially resulting in a loss of immunoreactivity.

Next, the beads were washed once with 1.5 mL of wash buffer (0.05% Tween-20 in PBS) and 3 times with 1 mL of PBS. Then, beads were incubated with 1 mL of The Blocking Solution (Candor) for 1 h at room temperature with end-over-end mixing. Subsequently, beads were washed twice with 1 mL of wash buffer. Next, beads were blocked in 1 mL of 3 M ethanolamine for at least 2 h at room temperature, with end-over-end mixing. Beads were then washed 3 times with 1 mL of wash buffer. Afterward, the beads were split into individual low-protein bind vials (Eppendorf). In each vial, 50 µg (Supplementary Fig. 2r) or 100 µg (Fig. 1c) of blocked beads were combined with 1 mL of biotinylated Flag-GS-HA standard or tissue protein lysates (input total protein concentration: 1–4 mg/mL for head and 2–17 mg/mL for a body in different experiments), and incubated overnight at 4 °C with end-over-end mixing. In Supplementary Fig. 2r, the background water control was diluted to the same extent as the highest concentration dilution of the Flag-GS-HA standard (1.25 × 10^−9^ M). Next, samples were washed 3 times with 1 mL of wash buffer, and incubated with 100 µL of 1.25 µg/mL avidin-HRP (Affymetrix) in 1× ELISA/ELISPOT diluent (5× stock solution, Affymetrix) for around 30 min (Supplementary Fig. 2r) to 1 h (Fig. 1c) at room temperature with shaking. Samples were then washed at least 4 times in 1 mL of wash buffer, followed by once in 200 µL of wash buffer. Finally, samples were incubated with 100 µL 1× TMB substrate solution (Affymetrix) at room temperature with shaking for 12 min (HA ELISA, Fig. 1c), 15 min (myc ELISA, Fig. 1c), or 30 min (Supplementary Fig. 2r). The reaction was terminated by adding 50 µL of stop solution (1 M H_3_PO_4_).

Sample absorbance values at *λ* = 450 nm and *λ* = 570 nm were measured using a spectrophotometer. In Fig. 1c, Nanodrop 8000 (ThermoFisher) was used, and in Supplementary Fig. 2r, Spectramax Paradigm plate reader (Molecular Devices) was used. For biotinylated Flag-GS-HA standard, final absorbance (Abs.) values were calculated as: Abs. = λ_450nm_ − λ_570nm_. For tissue samples, final Abs. values per mg of input protein were calculated as: $\text{Abs}./\text{mg protein}=\frac{{\lambda }_{450\text{nm}}-{\lambda }_{570\text{nm}}}{\text{mg input protein}}$. In Fig. 1c, normalization was performed by subtracting Abs. of 0 M standard peptide from Abs. of 1.04 × 10^−11^ M standard peptide, average *wt* head Abs./mg protein from head Abs./mg protein, and average *wt* body Abs./mg protein from body Abs./mg protein.

Shown in the top panel of Fig. 1c (Dilp2-HA-biotin ELISA) are representative results from two independent experiments. Shown in bottom panel of Fig. 1c (BirA*G3-ER-myc-biotin ELISA) are measurements from another similar head and body preparation of the same genotypes of flies. The three replicates from the same head and body preparation were measured across 2 runs. In Fig. 1c, *w[1118]* flies are used as *wt* controls.

### Quantitative tandem mass-tag (TMT) MS of BirA* *Drosophila* and mouse samples

Female and male adult flies were used in BirA*R118G-ER and BirA*G3-ER MS experiments and *w[1118]* flies were used as *wt* controls. Protein lysates and pulldowns were performed as described above (Preparation of *Drosophila* body parts, tissues, and dissections, Protein lysate preparation, and Streptavidin beads pulldowns sections). The different MS samples and their associated TMT labels are presented in Supplementary Figs. 4a, 7a, and 14a, b.

### On-bead digestion

Samples collected and enriched with streptavidin magnetic beads from *Drosophila melanogaster* legs/heads and mouse serum/teratomas were washed twice with 200 µL of 50 mM Tris-HCl buffer (pH 7.5), transferred into new 1.5 mL Eppendorf tubes, and washed 2 more times with 2 M urea/50 mM Tris (pH 7.5) buffer. Samples were incubated in 0.4 µg trypsin in 80 µL of 2 M urea/50 mM Tris buffer with 1 mM DTT, for 1 h at room temperature while shaking at 1000 × *g*. Following pre-digestion, 80 µL of each supernatant was transferred into new tubes. Beads were washed twice with 60 µL of 2 M urea/50 mM Tris buffer, and these washes were combined with the supernatant. The eluates were spun down at 5000 × *g* for 1 min and the supernatant was transferred to a new tube. Samples were reduced with 4 mM DTT for 30 min at room temperature, with shaking. Following reduction, samples were alkylated with 10 mM iodoacetamide for 45 min in the dark at room temperature. An additional 0.5 µg of trypsin was added and samples were digested overnight at room temperature while shaking at 700 × *g*. Following overnight digestion, samples were acidified (pH < 3) with neat formic acid (FA), to a final concentration of 1% FA. Samples were spun down and desalted on C18 StageTips as previously described^56^. Eluted peptides were dried to completion and stored at −80 °C.

#### TMT labeling of peptides

Desalted peptides were labeled with TMT (10-plex) reagents (ThermoFisher Scientific). Each 0.8 mg vial of TMT reagent was resuspended in 41 µL of MeCN. Peptides were resuspended in 100 µL of 50 mM HEPES and labeled with the TMT reagents as described in Supplementary Figs. 4a, 7a, and 14a, b. Samples were incubated at RT for 1 h with shaking at 1000 × *g*. TMT reaction was quenched with 8 µL of 5% hydroxylamine at room temperature for 15 min with shaking. TMT labeled samples were combined, dried to completion, reconstituted in 100 µL of 0.1% FA, and desalted on StageTips.

#### SCX stage tip fractionation of peptides

Single-shot analyses were performed on 50% of each sample. Desalted peptides were resuspended in 9 µL of 3% MeCN/0.1% FA and 4 µL was injected on a Q Exactive Plus (*Drosophila* samples) or HF-X (mouse samples) mass spectrometer (ThermoFisher Scientific, see methods below). In addition, flow throughs of combined samples were also analyzed via single shot and fractionation. To increase depth-of-coverage, 50% of the TMT labeled peptide sample was fractionated by strong cation exchange (SCX) StageTips packed with 3 disks of SCX (3 M Empore) material below 2 disks of C18 material. StageTips were conditioned with 100 µL of 100% MeOH, followed by 100 µL of 0.5% acetic acid/80% MeCN. Next, StageTips were equilibrated with 100 µL of 0.5% acetic acid, followed by 100 µL of 0.5% Acetic Acid/ 500 mM NH4AcO/ 20% MeCN, and finally with 100 µL of 0.5% acetic acid. Peptide samples were resuspended in 250 µL of 0.5% acetic acid and loaded onto the StageTips, washed 2× with 100 µL of 0.5% acetic acid, and transeluted from C18 material onto the SCX material with 100 µL of 0.5% acetic acid/80% MeCN. Three step-wise elutions from the C18 disks were completed as follows: the first fraction was eluted with 50 µL of 50 mM NH_4_HCO_3_: 20% MeCN (pH 5.15, adjusted with acetic acid), the second with 50 µL of 50 mM NH_4_HCO_3_: 20% MeCN (pH 8.25, adjusted with acetic acid), the third with 50 µL 50 mM NH_4_OH: 20% MeCN (pH 10.3, adjusted with acetic acid). Each eluate was collected separately and 200 µL of 0.5% acetic acid was added to each. Each fraction was desalted on C18 StageTips as described above, and samples were dried to completion.

#### Liquid chromatography and mass spectrometry

Desalted peptides were resuspended in 9 µL of 3% MeCN/0.1% FA and analyzed by online nanoflow liquid chromatography-tandem mass spectrometry (LC–MS/MS) using a Q Exactive HF-X or an Q Exactive Plus mass spectrometer (QE+, ThermoFisher Scientific) coupled online to a Proxeon Easy-nLC 1200 (ThermoFisher Scientific) as previously described^56^. Briefly, 4 µL of each sample was loaded at onto a microcapillary column (360 μm outer diameter × 75 μm inner diameter) containing an integrated electrospray emitter tip (10 μm), packed to approximately 24 cm with ReproSil-Pur C18-AQ 1.9 μm beads (Dr. Maisch GmbH) and heated to 50 °C. SCX fractionated samples were analyzed twice using two 4 µL injections using a 110 min LC–MS method using a Q Exactive HF-X (mouse original samples and first injection of flow-through samples) or a Q Exactive Plus (second mouse flow-through injections, all *Drosophila* samples) mass spectrometer. Mobile phase flow rate was 200 nL/min, comprises 3% acetonitrile/0.1% formic acid (Solvent A) and 90% acetonitrile /0.1% formic acid (Solvent B). The 110-min LC–MS/MS method used the following gradient profile: (min:%B) 0:2; 1:6; 85:30; 94:60; 95:90; 100:90; 101:50; 110:50 (the last two steps at 500 nL/min flow rate). Non-fractionated samples (single shots) were analyzed using a 260 min LC–MS/MS method on a Q Exactive HF-X (mouse samples) or a Q Exactive Plus (*Drosophila* samples) mass spectrometer. This method had the following gradient profile: (min:%B) 0:2; 1:6; 235:30; 244:60; 245:90; 250:90; 251:50; 260:50 (the last two steps at 500 nL/min flow rate). The Q Exactive HF-X or Q Exactive Plus were operated in the data-dependent mode acquiring HCD MS/MS scans (*r* = 15,000 for HF-X, 17,500 for QE+) after each MS1 scan (*r* = 60,000 for HF-X, 70,000 for QE+) on the top 12 (QE+) or top 20 (HF-X) most abundant ions using an MS1 target of 3 × 10^6^ and an MS2 target of 5 × 10^4^. The maximum ion time utilized for MS/MS scans was 120 ms; the HCD-normalized collision energy was set to 31 (HF-X) or 28 (QE+); the dynamic exclusion time was set to 20 s, and the peptide match and isotope exclusion functions were enabled. Charge exclusion was enabled for charge states that were unassigned, 1 and >7.

### BirA* MS data analysis

All protein trafficking MS data were analyzed using Spectrum Mill software package v 7.00 (mouse data) or 6.1 (*Drosophila* data) pre-release (Agilent Technologies). Similar MS/MS spectra acquired on the same precursor *m*/*z* within ±60 s were merged. MS/MS spectra were excluded from searching if they were not within the precursor MH+ range of −600–6000 Da (mouse data) or 750–4000 Da (*Drosophila* data) or if they failed the quality filter by not having a sequence tag length >0. These are standard exclusion criteria, and the precursors outside this mass range are typically not peptides. MS/MS spectra were searched against either all *Drosophila melanogaster* or mouse proteins annotated at UniProt database^57^ containing 46,519 (mouse) or 21,979 (*Drosophila*) proteins, and 264 (mouse) or 259 (*Drosophila)* common contaminants. All spectra were allowed ±20 ppm mass tolerance for precursor and product ions, 30% (*Drosophila*) or 40% (mouse) minimum matched peak intensity, and “trypsin allow P” enzyme specificity with up to 2 missed cleavages. The fixed modifications were carbamidomethylation at cysteine, and TMT6 at N-termini and internal lysine residues. Variable modifications included oxidized methionine and N-terminal protein acetylation and deamination (mouse only). Individual spectra were automatically designated as confidently assigned using the Spectrum Mill autovalidation module. Specifically, a target-decoy-based false-discovery rate (FDR) scoring threshold criteria via a two-step auto threshold strategy at the spectral and protein levels was used. First, peptide mode was set to allow automatic variable range precursor mass filtering with score thresholds optimized to yield a spectral level FDR of <1.2%. A protein polishing autovalidation was applied to further filter the peptide spectrum matches using a target protein level FDR threshold of 0. Following autovalidation, a protein–protein comparison table was generated, which contained experimental over control TMT ratios. For all experiments, non-*Drosophila* or non-mouse contaminants and reverse hits were removed. Furthermore, data were median normalized.

Next, we established threshold TMT ratios for hit-calling using positive control (PC) secreted/receptor and negative control (NC) intracellular lists. For the PC list, we used fly receptor and secretome lists^58^, as well as fly orthologs (using DIOPT^22^) of human receptome^59^, human secreted proteins annotated at UniProt^57^, and blood plasma MS (highly confident cumulative multiple-data set PeptideAtlas list^60^, and a list from humans with trauma^61^). PC proteins were also checked for the presence of a signal peptide^62^ and transmembrane (TM) domains^63^. For the NC list, we used high-confidence mitochondrial proteins^52^, as well as cytoskeletal and high-confidence transcription factors (nuclear)^58^. Proteins identified by our BirA*R118G-ER and BirA*G3-ER MS were compared to the PC and NC lists, and assigned to as being a PC or NC protein (Supplementary Figs. 4b, 6b, 7b, e). For each experiment (head or leg), there were four TMT ratio comparisons (BirA*-rep1/*wt*-rep1, BirA*-rep1/*wt*-rep2, BirA*-rep2/*wt*-rep1, BirA*-rep2/*wt*-rep2; rep means replicate) (Supplementary Figs. 4a and 7a). For each BirA*G3-ER comparison, we plotted the fraction $\frac{{\rm{\#PC}}}{{\rm{\#PC}}+{\rm{\#NC}}}$ above each log_2_BirA*/*wt* TMT ratio, and determined a threshold TMT ratio at which $\frac{{\rm{\#PC}}}{{\rm{\#PC}}+{\rm{\#NC}}}$ was generally greater than 0.9 (representative plot shown in Supplementary Fig. 4c; other plots available upon request). Based on the shapes of the curves, the thresholds were: leg 127N/126 TMT-ratio ≥ 0.43 at $\frac{{\rm{\#PC}}}{{\rm{\#PC}}+{\rm{\#NC}}} > \,0.93$; leg 127N/126 TMT-ratio ≥ 0.5 at $\frac{{\rm{\#PC}}}{{\rm{\#PC}}+{\rm{\#NC}}} > \, 0.92$; leg 129N/126 TMT-ratio ≥ 0.7 at $\frac{{\rm{\#PC}}}{{\rm{\#PC}}+{\rm{\#NC}}} > \, 0.95$; leg 129N/128C TMT-ratio ≥ 0.7 at $\frac{{\rm{\#PC}}}{{\rm{\#PC}}+{\rm{\#NC}}} > \, 0.93$; head 128N/127C TMT-ratio ≥ 0.8 at $\frac{{\rm{\#PC}}}{{\rm{\#PC}}+{\rm{\#NC}}} > \, 0.75\text{to}0.8$ (the $\frac{{\rm{\#PC}}}{{\rm{\#PC}}+{\rm{\#NC}}}$ vs 128N/127C TMT-ratio curve had significant fluctuation around the cutoff, and a manually-drawn best-fit curve had a $\frac{{\rm{\#PC}}}{{\rm{\#PC}}+{\rm{\#NC}}}$ of at least around 0.8 to 0.9); head 128N/129C TMT-ratio ≥ 0.9 at $\frac{{\rm{\#PC}}}{{\rm{\#PC}}+{\rm{\#NC}}} > \, 0.91$; head 130N/127C TMT-ratio ≥ 0 at $\frac{{\rm{\#PC}}}{{\rm{\#PC}}+{\rm{\#NC}}} > \, 1.0$; and head 130N/129C TMT-ratio ≥ 0.1 at $\frac{{\rm{\#PC}}}{{\rm{\#PC}}+{\rm{\#NC}}} > \, 0.83$ (approximate; midpoint between TMT-ratio = 0 at $\frac{{\rm{\#PC}}}{{\rm{\#PC}}+{\rm{\#NC}}}=0.67$ and TMT-ratio = 0.2 at $\frac{{\rm{\#PC}}}{{\rm{\#PC}}+{\rm{\#NC}}}=1.0$). For BirA*R118G-ER, the threshold TMT ratios were set based on the shapes of the curves: leg 129C/127C TMT-ratio ≥ 2.4 at $\frac{{\rm{\#PC}}}{{\rm{\#PC}}+{\rm{\#NC}}}=0.67$ (the next point is $\frac{{\rm{\#PC}}}{{\rm{\#PC}}+{\rm{\#NC}}}=1.0$); leg 129C/129N TMT-ratio ≥ 1.4 at $\frac{{\rm{\#PC}}}{{\rm{\#PC}}+{\rm{\#NC}}} > \, 0.86$; leg 127N/127C TMT-ratio ≥ 3.4 at $\frac{{\rm{\#PC}}}{{\rm{\#PC}}+{\rm{\#NC}}}=1.0$; leg 127N/129N TMT-ratio ≥ 2.8 at $\frac{{\rm{\#PC}}}{{\rm{\#PC}}+{\rm{\#NC}}}=1.0$; head 130C/128N TMT-ratio ≥ 1.2 at $\frac{{\rm{\#PC}}}{{\rm{\#PC}}+{\rm{\#NC}}}=1.0$; head 130C/130N TMT-ratio ≥ 0.8 at $\frac{{\rm{\#PC}}}{{\rm{\#PC}}+{\rm{\#NC}}}=1.0$; head 128C/128N TMT-ratio ≥ 0.4 at $\frac{{\rm{\#PC}}}{{\rm{\#PC}}+{\rm{\#NC}}}=0.7$; and head 128C/130N TMT-ratio ≥ 0.2 at $\frac{{\rm{\#PC}}}{{\rm{\#PC}}+{\rm{\#NC}}}=0.89$. Enrichment score (E-S) was defined as the number of comparisons in which a specific protein’s TMT ratio exceeds the threshold TMT ratio (Fig. 2c and Supplementary Figs. 6a, 7d, g). Thus, proteins with E-S of 0 are background, proteins with a E-S of 1 are lower-confidence hits, and proteins with E-S of 4 are highest confidence hits. A similar analysis was used for the teratoma and serum TMT MS data sets. The PC list was UniProt annotated secreted proteins, while the NC list was the UniProt overlapping list of transcription factors and nuclear proteins, and cytoskeletal genes^57^. Note that the NC list was compared with secreted, receptors, ER proteins, and overlapping genes were removed. The threshold TMT ratio was chosen at which $\frac{ \% {\rm{NC}}}{ \% {\rm{PC}}}\le {\rm{FPR}}({\rm{false}}\; {\rm{positive}}\; {\rm{rate}})$ (FPR = 0.1 for teratomas and FPR = 0 for serum). Proteins with E-S of 0 are background, proteins with E-S of 1 are lower-confidence hits, and proteins with E-S of 9 are highest confidence hits. Note that in Figs. 2, 7a, b, g, and Supplementary Figs. 4b–d, f, 6a, b, d, j, 14c–h, k–l, 15g, k–l, means (and where applicable, SEMs) were calculated from log_2_ values of TMT ratios.

To obtain human ortholog information, we used DIOPT, versions 5 and 6^22^. MS-identified proteins at different scores were examined for the presence of putative signal peptides, using SignalP program^62^ (SignalP4.1: Fig. 2d; Supplementary Figs. 4f and 6d; SignalP5.0^64^: Fig. 7c, d; Supplementary Figs. 14k–l, 17b, c). Moreover, proteins were examined for their presence in our fly total hemolymph protein MS (Supplementary Figs. 4g–i and 17; Supplementary Data 2 and Supplementary Table 3). In addition, based on UniProt annotation^57^, we determined whether the identified proteins could be ER-resident (Supplementary Figs. 5a, 6f, 14m, n). Using the SecretomeP program^65^, we examined whether some of the proteins could be unconventionally/non-classically secreted (Supplementary Figs. 5b and 6g). We also obtained tissue mRNA expression information from FlyAtlas microarray^15^ (data shown in Fig. 2e, f; Supplementary Fig. 6h, i) and, as confirmation for these results, RNAseq^66^ data sets. In addition, protein abundance information was from an integrated entire organism PAX database for *Drosophila melanogaster* and mouse^67^ (Fig. 7e, f and Supplementary Fig. 6c–f). In Supplementary Fig. 8, categorization of hits (E-S ≥ 1) into broad categories was performed manually using data from FlyBase^68^ and NCBI Gene^69^. We compared the MS-identified proteins to fly and mouse orthologs (using DIOPT version 5.3^22^) of mammalian adipocyte^7,70–78^ and myocyte^79–86^ secretomes. For higher confidence, we required a protein to be identified in at least 2 adipocyte or myocyte secretome data sets to be considered an adipocyte or myocyte secreted protein for our analysis in Fig. 2g, h, and Supplementary Fig. 15i, j. Mouse serum hits were also compared to mammalian whole blood (cells removed) proteomics data sets (highly confident cumulative multiple-data set PeptideAtlas list^60^, and a list from humans with trauma^61^).

### Hemolymph MS proteomics

We performed whole hemolymph MS proteomics as part of our study to gain insight into which proteins may be circulating in *Drosophila* (Supplementary Data 2 and Supplementary Table 3; Supplementary Fig. 4g–i). We identified a total of 1561 proteins, including 688 proteins with poorly-characterized functions (“Computed Genes”/CGs; FlyBase^68^ r551). The following hemolymph MS experiments were performed: *Ore*^*R*^ unfractionated hemolymph, *myoglianin* overexpressing^45^ unfractionated hemolymph, methanol-extracted peptides from *Ore*^*R*^ hemolymph, size-fractionated *Ore*^*R*^ hemolymph, 4-week old *Ore*^*R*^ fly unfractionated hemolymph, glycopeptide pulldown *Ore*^*R*^ hemolymph, and non-tryptic (open) *Drosophila* database search of <3 kD fraction and O-glycopeptide pulldown experiment. In Supplementary Data 2, Supplementary Table 3 and Supplementary Fig. 6g–i, the MS data was analyzed by counting the number of times a peptide or different peptides for one protein was observed in each of the above 7 experiments (for the purposes of this analysis, each of the above was counted as an experiment). Each identified protein was assigned to a PC secreted/receptor or NC intracellular lists as above (see “BirA* MS data analysis” section). Note that for this analysis, if a protein was assigned to both NC and PC list, it was assigned to the “other” category. In addition, a targeted analysis was performed on *Ore*^*R*^ unfractionated hemolymph for predicted or known^87^ tryptic peptides; this analysis was not included in the above results.

We used methanol to precipitate proteins and enrich for smaller peptides^88–90^. A total of 38 µL of hemolymph was precipitated in 80% methanol at −80 °C for 7 h. Next, the sample was centrifuged at 14,000 × *g* for 15 min at 4 °C, and the supernatant was dried in a speed-vac for 2 h.

Previously established methods^91^ were used for hemolymph size-fractionation. A total of 100 µL of hemolymph was dissolved in urea buffer (8 M urea, 50 mM Tris (pH = 7.5), 75 mM NaCl) with 0.5% dithiothreitol (DTT), and incubated at 60 °C for 1 h. Next, samples were incubated in 15 mM iodoacetamide for 30 min in the dark at room temperature with shaking, followed by 5 mM DTT for 15 min at room temperature in the dark. Next, we used Amicon Ultra 0.5 mL columns (Millipore) according to the manufacturer’s instructions. Columns (3 K cutoff; Millipore) were pre-spun in urea buffer for 25 min at room temperature at 14,000 × *g*. Next, the hemolymph sample was applied and centrifuged for 40 min at 14,000 × *g* at room temperature. Further, 400 µL of urea buffer was applied and centrifugation was repeated. This was repeated twice more to obtain the <3 kD sample in the flow-through. Next, the column was inverted into a new vial and centrifuged at 1000 × *g* for 2 min at room temperature. This was repeated four times with 100 µL of urea buffer to wash. The latter >3 kD fraction was transferred to pre-spun (with urea buffer at 30 min for 14,000 × *g*) 10 K membranes (Millipore), and centrifuged at 14,000 × *g* for 30 min at room temperature. The columns were washed three times with 420 µL of urea buffer to obtain the 3–10 kD fraction. Further, to obtain the >10 kD fraction, the columns were inverted and eluted to new tubes by centrifuging at 1000 × *g* for 2 min at room temperature, followed by four times washing with 100 µL of urea buffer and centrifuging. The latter >10 kD fraction was transferred to pre-spun (with urea buffer at 18 min for 14,000 × *g*) 30 K membranes (Millipore), and centrifuged at 14,000 × *g* for 15 min at room temperature. The columns were washed three times with 420 µL of urea buffer to obtain the 10–30 kD fraction. Further, to obtain the >30 kD fraction, the columns were inverted and eluted to new tubes by centrifuging at 1000 × *g* for 2 min at room temperature, followed by twice washing with 50 µL of urea buffer and centrifuging. The >30 kD sample was diluted 1:10 in 100 mM ammonium bicarbonate, pH = 8.3 (final urea concentration was 0.8 M), 1 mM calcium chloride, and digested with sequencing grade-modified trypsin (Promega) at 37 °C overnight with shaking, according to the manufacturer’s instructions. Samples were then acidified with trifluoroacetic acid (TFA) to a final pH < 2. The samples were subsequently purified using tC18 sep-pak columns (Waters) according to standard protocols^92^. We used 200 mg cartridges for >10 kD fractions and 100 mg cartridges for <10 kD fractions. Columns were conditioned using 3 mL (200 mg cartridges) or 2 mL (100 mg) acetonitrile, followed by twice with 3 mL (200 mg) or 2 mL (100 mg) 50% acetonitrile in water and 0.5% acetic acid in water. Next, 3 mL (200 mg) or 2 mL (100 mg) of 0.1% TFA in water was passed through the column twice, and samples were slowly loaded. Columns were next washed twice with 3 mL (200 mg) or 2 mL (100 mg) of 0.1% TFA in water, followed by 360 µL of 0.5% acetic acid in water. The samples were twice eluted with 0.9 mL of 50% acetonitrile with 0.5% acetic acid in water. Finally, the samples were dried in a speed-vac and frozen at −80 °C.

Glycosylated peptide pulldowns were performed as previously described^93–96^. Hemolymph (125 µL) was dissolved in a 0.4 M NH_4_HCO_3_ (pH = 8.3), 0.1% sodium dodecyl sulfate (SDS), and 8 M urea. Next, 10 mM TCEP (Tris (2-carboxyethyl) phosphine)) was added and the mixture was incubated at 58 °C for 1 h 40 min. Then, 14 mM iodoacetamide was added and incubated at room temperature for 30 min in the dark with mixing. This was followed by the addition of 5 mM TCEP for 15 min at room temperature in the dark. The sample was then diluted 1:10 in a final concentration of 100 mM NH_4_HCO_3_, pH = 8.3 (final urea concentration was 0.8 M), 1 mM calcium chloride, and digested with sequencing grade-modified trypsin (Promega) at 37 °C overnight with shaking, according to manufacturer’s instructions. The sample was then centrifuged for 12,000 × *g* for 10 min and the supernatant was acidified with TFA to a final pH < 2. The sample was subsequently purified using a tC18 sep-pak column (Waters). The column was conditioned using 3 mL of acetonitrile, followed by twice with 3 mL of 50% acetonitrile with 0.1% TFA in water. Next, 3 mL of 0.1% TFA in water was passed through the column twice, and the sample was slowly loaded. The column was next washed thrice with 3 mL of 0.1% TFA in water. The sample was twice eluted with 0.9 mL of 50% acetonitrile with 0.1% TFA in water. Subsequently, 10 mM sodium periodate was added and the sample was incubated for 1 h at 4 °C in the dark. Next, the 2 mL sample was diluted with 16.2 mL of 0.1% TFA in water and purified with a 200 mg tC18 sep-pak column (Waters) in the same way as described above, with the modification that the sample elution was with 0.1% TFA in 80% acetonitrile in water. Next, 300 µL of Affi-Gel hydrazide slurry (Biorad) was centrifuged at 845 × *g* for 30 s, washed once with water, centrifuged at 845 × *g* for 30 s, and water removed. The sample was then added to the washed hydrazide resin and incubated with end-over-end mixing at room temperature overnight. The resin was centrifuged at 2500 × *g* for 5 min, and the unbound peptides (flow-through/supernatant and the washes) were the non-glycosylated peptides. The resin was then washed three times each with 1.5 M NaCl in water, water, and 100 mM ammonium bicarbonate in water (pH = 7.5). Between washes, the centrifugations were at 2500 × *g* for 5 min up to the second water wash, then 2500 × *g* for 6 min up to the first ammonium bicarbonate wash, and then 2500 × *g* for 10 min. Subsequently, the resin was resuspended in 300 µL of G7 reaction buffer (NEB), 30 U/µL of PNGase F (NEB) were added, and incubated at 37 °C overnight with shaking. Next, the sample was centrifuged at 2500 × *g* for 10 min, and the supernatant (and the subsequent washes) was collected to a triply water-washed 1-dram glass vial. The resin was washed twice with water. Further, the resin was resuspended in 300 µL of 0.2 M citrate-phosphate buffer (pH = 5.0), mixed with 12 µL of PNGase A (Roche), and incubated at 37 °C overnight with shaking. Next, the sample was centrifuged at 2500 × *g* for 10 min, and the supernatant (and the subsequent washes) was collected to the same triply water-washed 1-dram glass vial that contained the PNGase F supernatant. The resin was washed twice with water. The PNGase F and PNGase A-released sample consisted of N-linked glycosylated peptides. To release the O-glycosylated peptides, the resin was incubated with 600 µL of 0.1 M NaOH for 4 h at 45 °C with shaking, 600 µL of 0.3 M acetic acid in water was added on ice, and the supernatant from a 2500 × *g* for 10 min centrifugation was collected. Next, the non-glycan sample was dried to a final volume of around 20% using speed-vac. Further, non-glyco, N-glyco, and O-glyco peptide samples were purified with tC18 sep-pak (Waters) as above, with 0.9 mL of 50% acetonitrile with 0.1% TFA in the water elution step. Finally, the samples were dried using speed-vac.

Other protein samples were digested with sequencing grade-modified trypsin (Promega Corp) at pH = 8.3 (50 mM ammonium bicarbonate) overnight at 37 °C with slight shaking. Peptides were acidified with 0.1% TFA and purified using C18 Zip-tips (Millipore) according to the manufacturer’s instructions and final elutions were dried to around 10 µL. 3–5 µL were loaded onto the LC–MS/MS system.

Peptides were analyzed by positive ion mode LC–MS/MS using a high-resolution hybrid Orbitrap Elite mass spectrometer (ThermoFisher Scientific) via CID with data-dependent analysis (DDA) using a Top 5 approach (1 full FT-MS scan followed by 5 MS/MS CID scans). Maximum injection time was 50 ms for MS and 100 ms for MS/MS with 1 microscan for both modes. Isolation width was 2.3 Da and dynamic exclusion time was set to 90 s. Peptides were delivered and separated using an EASY-nLC I nanoflow HPLC (ThermoFisher Scientific) at 300 nL/min using self-packed 15 cm length × 75 μm inner diameter C18 fritted microcapillary Picofrit columns (New Objective). Solvent gradient conditions were 140 min from 3% B buffer to 38% B (B buffer: 100% acetonitrile; A buffer: 0.1% formic acid/99.9% water). MS/MS spectra were analyzed using the Mascot 2.5 search engine by searching the reversed and concatenated UniProt DROME protein database (version 20130918 containing 21,002 protein sequence entries) and the dmel-all translation database (version r522, 2012, 43571 entries) with a parent ion tolerance of 18 ppm and fragment ion tolerance of 0.80 Da. Carbamidomethylation of Cys (+57.0293 Da) was specified in Sequest as a fixed modification and oxidation of Met (+15.9949 Da) and deamidation of Asn/Gln (+0.9840 Da) as variable modifications. Results were imported into Scaffold 4.0 software (Proteome Software) with a peptide threshold of around 75%, protein threshold of 95%, resulting in a peptide false-discovery rate (FDR) of <1.5%.

### Climbing assays

Climbing assays were performed as part of this study^26,45,97^. Each vial had around 10–20 adults (males and females were separated). On the day of the experiment, flies were transferred to empty vials without food (without the use of CO_2_). For climbing assays, flies were tapped to the bottom of the vial and the number of flies able to climb above 6 cm was counted after 10 s. After the climbing tests, flies were transferred back to regular food-containing vials.

Climbing tests with males and females were performed in separate vials and results in Fig. 3a–c, Supplementary Figs. 1i and 9t–v are presented as averages of across sexes. All collected climbing data are presented and analyzed (cumulative across the number of experiments indicated below), and no data were excluded from the analysis. In Fig. 3a, the biological replicates were *n* = 4 from 1 experiment (*LPP-Gal4>*control (*Luc-i*)), *n* = 6 cumulative across 2 experiments (*LPP-Gal4>*control (*w-i*)), *n* = 20 cumulative across 3 experiments (*LPP-Gal4>*control (*attP*)), *n* = 12 cumulative across 2 experiments (*LPP-Gal4>CG31326-i-1*), and *n* = 10 cumulative across 2 experiments (*LPP-Gal4>CG2145-i-1*). In Fig. 3b, the biological replicates were *n* = 12 cumulative across 2 experiments (*LPP-Gal4>*control (*v-i*)), *n* = 8 cumulative across 2 experiments (*LPP-Gal4>CG31326-i-2*), *n* = 8 cumulative across 2 experiments (*LPP-Gal4>CG31326-i-3*), and *n* = 4 cumulative across 2 experiments (*LPP-Gal4>CG4332-i-1*). In Fig. 3c, the biological replicates were *n* = 14 cumulative across 2 experiments (*LPP-Gal4>*control (*w-i*)), *n* = 9 from 1 experiment (*LPP-Gal4>*control (*GFP-i*)), *n* = 13 cumulative across 2 experiments (*LPP-Gal4>*control (*Luc-i*)), *n* = 13 cumulative across 2 experiments (*LPP-Gal4>CG4332-i-2*), and *n* = 7 cumulative across 2 experiments (*LPP-Gal4>CG2145-i-2*).

### Immunostaining and confocal microscopy

Immunostaining and confocal microscopy for other samples were performed as part of this study^26,39,52^. After dissections (see “Preparation of *Drosophila* body parts, tissues, and dissections” section), thoraxes in Fig. 3i–x, Supplementary Fig. 9a–n were fixed in 4% PFA (methanol-free) in PBS at room temperature with gentle shaking for 1 h. In other figures, organs were fixed in 4% PFA (methanol-free) in PBS at room temperature with gentle shaking for 30 min. Tissues were then washed four times for 10 min each in PBS with 0.1% Triton X-100 (PBST) at room temperature with gentle shaking.

Tissues were blocked with 5% normal goat serum (NGS, Vector Laboratories) in PBST for 30 min at room temperature, with gentle shaking in the dark. In Fig. 3i–x, Supplementary Fig. 10 tissues were blocked with 5% NGS in PBST with 1% BSA (filtered through 0.45 µm membrane; PBST-B) for at least an hour at room temperature. Next, samples were incubated in primary antibody dilutions in PBST or in PBST-B with 5% NGS (the latter used in Fig. 3i–x, Supplementary Fig. 10) overnight at 4 °C with gentle shaking (51 rpm) in the dark. Primary antibodies and reagents were: 1:200 mouse anti-myc (clone 9E10, Santa Cruz Biotechnology; used in Supplementary Fig. 2kk–rr); 1:500 rabbit anti-myc (clone 71D10, Cell Signaling Technology; used in Supplementary Fig. 2cc–jj); neat (Supplementary Fig. 2s–aa) or 1:10 (Supplementary Fig. 2cc–jj) mouse anti-Cnx99A (ER marker; Developmental Studies Hybridoma Bank [DSHB])^98^; 1:2000 rabbit anti-ref(2)P (gift of Andreas Brech)^99^; 1:100 mouse anti-ubiquitin (clone FK2, Enzo Life Sciences); 1:500 streptavidin-Alexa Fluor (AF) 647 (Invitrogen; in some experiments added in secondary antibody solution); 1:200 rat anti-HA (clone 3F10 [100 µg/mL stock], Roche); and 1:100 phalloidin-AF 660 (Invitrogen; in some experiments added in secondary antibody solution). On the next morning, samples were washed 4 times for 10 min per wash in PBST or PBST-B (Fig. 3i–x; Supplementary Fig. 2cc–rr, 10a–r) at room temperature in the dark, with gentle shaking. In Fig. 3i–x and Supplementary Fig. 10, tissues were blocked in PBST-B with 5% NGS for at least 30 min at room temperature.

Tissues were incubated overnight at 4 °C in dilutions of the secondary antibody and additional staining reagents in PBST or PBST-B with 5% NGS (Fig. 3i–x, Supplementary Fig. 10), with gentle shaking (51 rpm) in the dark. The secondary antibodies and reagents were: 1:500 donkey anti-mouse AF488 (Invitrogen), 1:1000 goat anti-mouse AF488, 1:500 goat anti-rat AF488 (Invitrogen), 1:500 donkey anti-rabbit AF488 (Invitrogen), 1:500 goat anti-mouse AF555 (Invitrogen), 1:1000 goat anti-rabbit AF568, 1:500 donkey anti-rabbit AF594 (Invitrogen), 1:500 donkey anti-rabbit AF555 (Invitrogen), 1:500 donkey anti-rat AF594 (Invitrogen), 1:50 goat anti-HRP TRITC (rhodamine; Jackson ImmunoResearch Laboratories 123-025-021), 1:100 phalloidin AF660 (Invitrogen), 1:100 phalloidin AF350 (Invitrogen), 1:100 phalloidin AF546 (Invitrogen), 1:100 streptavidin AF647 (Invitrogen), and 1:2000 DAPI. Next, tissues were washed 4 times, 15 min each in PBST at room temperature with gentle shaking in the dark. Finally, tissues were mounted in Vectashield (Vector Laboratories) with two layers of transparent tape on either side to avoid flattening the tissue with the coverslip.

BODIPY (lipids) staining of FB was done as previously described^100^. After fixation, abdomens were washed 4 times for 10 min each in PBS at room temperature with gentle shaking. Alternatively, after secondary antibodies and washes, abdomens were washed twice quickly in PBS. Abdomens were then incubated with 1:1000 DAPI (Molecular Probes) for 30 min at room temperature with gentle shaking, and washed four times for 15 min each in PBS with gentle shaking. Next, abdomens were incubated with 1:1000 BODIPY493/503 (Invitrogen) in PBS for 30 min at room temperature with gentle shaking, and washed twice in PBS. Abdomens were mounted as described above.

Confocal images were acquired using Zeiss LSM780. Original (non-adjusted) images are shown, unless otherwise indicated. Where indicated, brightness and contrast were adjusted equally in the whole image and equally between experimental groups using ZEN2012 software (Carl Zeiss). In Fig. 1b (lower panel) and Supplementary Fig. 2e–h, m–p, brightness and contrast were adjusted to 35 and 3.75, respectively. Figure 1b is a maximum intensity projection (MIP) from 2 slices cropped from the blue region of the same sample shown in Supplementary Fig. 3a–d. In Supplementary Fig. 2cc–rr, MIPs of 2 slices are shown. For muscles/thoraxes in Supplementary Fig. 9a–n (protein aggregate formation experiments) around 5–6 animals were dissected, and thorax was cut into 2 or 4 fragments. One (occasionally two) confocal image per fragment piece was taken, representing at least 3 animals. Where indicated, maximum intensity projections were generated using ZEN2012 software (Carl Zeiss). Protein aggregate analysis samples (representative images in Supplementary Fig. 9a–n) were dissected, stained, and imaged across multiple experimental days, and hence imaging conditions may have varied slightly. For protein aggregate representative images in Supplementary Fig. 9a–n, cropped images (original: 134.95 µm × 134.95 µm; cropped: 101.21 µm × 101.21 µm) are shown for ease of visualization. In Supplementary Fig. 9o–s, samples were processed across multiple experimental days. In Fig. 3i–x, Supplementary Fig. 10, MIPs of 4 slices are shown. In Fig. 3i–x, cropped images (original: 134.95 µm × 134.95 µm; cropped: 80.5 µm × 80.5 µm) are shown for ease of visualization. In Fig. 3i′–l′ and m′–p′ shown are cropped images (final size: 27.15 µm × 27.15 µm) from square regions of interest in Fig. 3i–l and m–p. In Fig. 3i′′–l′′ shown are cropped images (final size: 15.88 µm × 15.88 µm) from the blue square region of interest in Fig. 3i–l. For images in Fig. 3l, l′, l′′, brightness and contrast for the actin (phalloidin) channel only were adjusted equally within the image to visualize actin structures; all other channels were not adjusted. In Supplementary Fig. 10b, c, e, f, h, i, for actin (phalloidin) channel only, brightness and contrast were adjusted to 45 and 7.95 (equally within and between images), respectively, to visualize FB cell borders.

### Image quantification

The areas of protein aggregates were quantified using previously established methods^26^. Control and experimental groups of images were analyzed in the same way. Representative single confocal slice images (uncropped) of p62/ref(2)P staining were converted to single color tiff files using ZEN 2012 (Carl Zeiss). Next, using FIJI (FIJI is just image J)^101^, images were converted to 8-bit grayscale, and threshold was adjusted in order to use the analyze particles function. The maximum entropy method^102^ was used in Fig. 3d, e, f, h and Supplementary Fig. 9w, and the triangle method^103^ was used in Fig. 3f. Next, the analyze particles function was used, in which the minimum particle size was 10 pixels^2^ in Fig. 3d, e, g, h and Supplementary Fig. 9w, and 20 pixels^2^ in Fig. 3f.

### Mouse Immunofluorescent Analyses

Frozen sectioned tissues were thawed at room temp for 10 min. The slides were rinsed in PBS for 5 min then blocked with 1.5% Seablock (ThermoFisher) in PBS + 0.25% Triton-X block buffer for 1 h at room temperature. The primary antibody mixture (diluted in block buffer) was added and the slides were incubated at 4 °C overnight. Primary antibodies used in the study were as follows: GFP (Aves labs, GFP-1020, 1:500), RFP Tag (Invitrogen, MA5-15257, 1:500), BirA 6C4c7 (Abcam, ab232732, 1:300), Alexa Fluor™ Plus 647 Phalloidin (ThermoFisher, A30107, 1:1000). The secondary antibody used were Goat anti-Mouse IgG1 Cross-Adsorbed Secondary Antibody, Alexa Fluor 555 (ThermoFisher, A-21127, 1:1000) Donkey anti-Mouse IgG, Alexa Fluor 594, Donkey anti-Chicken Alexa Fluor 488), and streptavidin DyLight649 conjugate (Vector labs, SA-5649-1, 1:1000). Slides were incubated with 1 mg/mL Hoechst 33342 (Molecular Probes) in PBS for 5 min to stain the nuclei prior to being mounted in ProLong Gold Antifade Reagent (Life technologies) and imaged at ×10 or ×63 using the Leica SP8 confocal microscope.

mESCs were cultured on glass coverslips for three days and then fixed in 4% PFA for 15 min, room temperature. Coverslips were washed three times with 1 × PBS for 5 min each and then permeabilized in 0.25% Triton X-100 for 5 min. Coverslips were blocked in 1 × PBS + 0.25% Triton X-100 + 2% Seablock (ThermoFisher, 37527) for 1 h at room temperature. Primary antibody was diluted in block (above) and incubated for 16 h at 4 °C. Primary antibody: mouse anti-BirA (Abcam, ab232732, 1:500). The following day, coverslips were washed with 1 × PBS + 0.25% Triton X-100, four times for 5 min each. Secondary antibody and streptavidin conjugate were diluted in block (above) and incubated at room temperature for 1 h. Secondary antibodies: Goat anti-Mouse IgG1 Cross-Adsorbed Secondary Antibody, Alexa Fluor 555 (ThermoFisher, A-21127, 1:1000), and streptavidin DyLight649 conjugated (Vector labs, SA-5649-1, 1:1000). Coverslips were then washed with 1 × PBS + 0.25% Triton X-100, four times for 5 min each. Coverslips were incubated with 1 mg/mL Hoechst 33342 (Molecular Probes) in 1 × PBS for 10 min to stain the nuclei prior to being mounted onto a slide with Fluoromount G (ThermoFisher, 00-4958-02) and imaged at ×40 or ×63 using the Leica SP8 confocal microscope.

### Histology

Frozen sectioned tissues were thawed at room temperature for 10 min and rinsed in PBS for 5 min followed by a brief rinse in water. The sections were stained for 30 × 45 sec in hematoxylin then for 15–30 s in eosin and finally counterstained with fast red for 1 min. Samples were scanned at high resolution (×10) on a Zeiss Axio Scan.Z1 Slide Scanner to generate high-resolution tiled image files of the tissue section.

### Data analysis and statistics

Data was analyzed using ZEN2012 software (Carl Zeiss), FIJI, Adobe Photoshop CC2019 (integrated density calculations in Fig. 1d′), Microsoft Excel, GraphPad Prism 7, OriginPro 2017 or 2020, Emperia Studio analysis software (LiCor; Version 1.3.0.83) and ImageStudio (Version 5.2.5) for western blot analysis, R (version 4.0.0 (2020-04-24), Platform: x86_64-apple-darwin17.0 (64-bit); RStudio Version 1.3.959), Biorad Chemidoc MP acquisition software (ImageLab Touch 2.4), Nanodrop acquisition software (ND8000 version 2.2.0), and Spectramax Paradigm acquisition software (Molecular Devices; Softmax Pro 6.2). Additionally, SignalP4.1, SignalP5, TMHMM (v 2.0), SecretomeP2.0 were used. Data is shown as mean ± standard error of the mean (SEM), unless otherwise indicated in the Fig. legend. Biological replicates are shown, unless otherwise indicated. In Fig. 1c, using GraphPad Prism, in the bottom panel, an unpaired two-tailed t-test was performed (*t* = 1.928, df = 8). A one-way ANOVA in Fig. 1d′ (*p* = 0.0015, *F* = 6.005, df = 23), Fig. 3a–c (a: *p* < 0.0001, *F* = 11.05, df = 216; b: *p* < 0.0001, *F* = 8.279, df = 170; c: *p* < 0.0001, *F* = 11.61, df = 213), Supplementary Fig. 1i (*p* = 0.0008, *F* = 4.367, df = 54) using GraphPad Prism, was performed, together with linear two-stage step-up false-discovery rate (FDR) calculation of Benjamini, Krieger, Yekutieli. Note that for each RNAi stock center collection, to account for multiple comparisons testing, the statistical calculation took into account additional tested *LPP-Gal4>RNAi* groups which did not show a phenotype in both climbing-ability and protein aggregate formation assays. Adjusted *p*-values (*q*-values) are shown. In Fig. 3d–h, an unpaired two-tailed t-test (assuming unequal variances for Fig. 3h) was performed using Microsoft Excel (d; *t* = 2.228, df = 10; e: *t* = 2.160, df = 13 (*CG31326-i-3*), *t* = 2.201, df = 11 (*CG31326-i-2*), *t* = 2.144, df = 14 (*CG4332-i-1*); f: *t* = 2.179, df = 12 (*CG2145-i-1*), *t* = 2.145, df = 14 (*CG31326-i-1*); g: *t* = 2.228, df = 10 (*CG31326-i-3*), *t* = 2.201, df = 11 (*CG31326-i-2*); h: *t* = 2.571, df = 5). In Supplementary Fig. 9w, an unpaired two-tailed t-test was performed using GraphPad Prism (*t* = 2.247, df = 18). In Supplementary Fig. 2aa, one-way ANOVA (*F*_column_ = 29.75, df = 27) and Holm–Sidak’s multiple comparisons test were performed and adjusted *p*-values are shown. In Supplementary Fig. 1a–h, log-rank test using the Mantel–Cox procedure was performed using Graphpad Prism (a: chi square = 3.051, df = 1; b: chi square = 2.651, df = 1; c: chi square = 0.6318, df = 1; d: chi square = 0.6534, df = 1; e: chi square = 18.79, df = 1; f: chi square = 5.892, df = 1; g: chi square = 3.794, df = 1; h: chi square = 4.396, df = 1). In Supplementary Figs. 4d and 14g, h, a linear regression analysis were performed using Graphpad Prism. In Fig. 2d–h, Supplementary Figs. 4e, h, i, 5a, b, 6c, e–g, 7h, using GraphPad Prism, a two-sided chi-squared test was performed. In Supplementary Fig. 5c, using GraphPad Prism, a one-way ANOVA (*F* = 4.586, df = 2285) and Kolmogorov–Smirnov tests were performed. In Supplementary Fig. 5e, using GraphPad Prism, a two-sided chi square test was performed (chi square = 6.198, df = 1). In Supplementary Fig. 5f, OriginPro 2017 was used for the statistical calculation and data was graphed using GraphPad Prism. A Bigaussian fit was performed on the data, and the fitted peak centers (*x*_c_) were compared using the *F*-test and Aikaike’s Information Criterion Test (AIC) (*F* = 5.17883, df = 1). In Supplementary Fig. 6h, i, using GraphPad Prism, a chi square test for trend was performed. In Supplementary Figs. 6k and 16 using GraphPad Prism, a two-sided chi-squared test was performed between observed and expected values. In Supplementary Fig. 9t–v using GraphPad Prism, a one-way ANOVA was performed (t: *p* = 0.0004, *F* = 4.109, df = 50; u: *p* = 0.0004, *F* = 5.700, df = 34; v: *p* < 0.0001, *F* = 8.553, df = 34), together with linear two-stage step-up false-discovery rate (FDR) calculation of Benjamini, Krieger, Yekutieli. Please note that for each RNAi stock center collection, to account for multiple comparisons testing, the statistical calculation took into account additional tested *Dmef2-Gal4>RNAi* groups for other genes which did not show a phenotype in both climbing-ability and protein aggregate formation assays. Adjusted *p*-values (*q*-values) are shown. In Fig. 7c, d, h, i; Supplementary Figs. 14i, j, m–o, 15h, i, j using GraphPad Prism, two-sided Fisher’s exact tests were done. In Fig. 7e, f, using GraphPad Prism, a Kruskal–Wallis test with Benjamini, Krieger, Yekutieli Linear two-stage step-up FDR was done (serum: *p* = 0.0386, Kruskal–Wallis statistic = 16.27; teratoma: *p* < 0.0001, Kruskal–Wallis statistic = 125.1).

### Reporting summary

Further information on research design is available in the Nature Research Reporting Summary linked to this article.

## Supplementary information

Supplementary information

Description of Additional Supplementary Files

Supplementary Data 1

Supplementary Data 2

Supplementary Data 3

Supplementary Data 4

Supplementary Data 5

Supplementary Data 6

Supplementary Movie 1

Supplementary Movie 2

Supplementary Movie 3

Reporting Summary

## Data Availability

The original mass spectra for all experiments, and the protein sequence databases used for searches have been deposited in the public proteomics repository MassIVE (https://massive.ucsd.edu) and are accessible at 10.25345/C5NB8W with the accession number MSV000086664 (mouse and fly BirA* data sets), and at 10.25345/C5XN4Z with the accession number MSV000086291 (fly hemolymph data sets). The following publicly available data sets/databases were used: UniProt database (https://www.uniprot.org/; *Drosophila* (DROME; https://www.uniprot.org/proteomes/UP000000803), mouse (https://www.uniprot.org/proteomes/UP000000589), and human (https://www.uniprot.org/proteomes/UP000005640)), DIOPT (https://www.flyrnai.org/cgi-bin/DRSC_orthologs.pl), GLAD (https://www.flyrnai.org/tools/glad/web/), Signaling Receptome (http://www.receptome.org/), human plasma proteome data sets (http://www.peptideatlas.org/repository/repository_public_Hs_Plasma2.php (PeptideAtlas) and Supplementary Table 1 in ref. ^61^), SignalP database (http://www.cbs.dtu.dk/services/SignalP/ and http://www.cbs.dtu.dk/services/SignalP-4.1/), *Drosophila* mitochondrial proteome (Supplementary Data 1 in ref. ^52^), TMHMM (http://www.cbs.dtu.dk/services/TMHMM/), SecretomeP (http://www.cbs.dtu.dk/services/SecretomeP/), FlyAtlas microarray (http://flyatlas.org/atlas.cgi), *Drosophila* RNAseq (http://www.modencode.org/celniker/), PAXdb (https://pax-db.org/), NCBI Gene (https://www.ncbi.nlm.nih.gov/gene/), FlyBase (https://flybase.org/), mammalian adipocyte secretomes (Tables S1–S5 in ref. ^7^, Supplementary Data Table C in ref. ^70^, Supplementary Data Table in ref. ^71^, Supplementary Table S1 in ref. ^72^, Supplementary Table—Secretome in ref. ^73^, Supplementary Table S1 in ref. ^74^, Data File S1 in ref. ^75^, Table 2 and Supplementary Table 1 in ref. ^77^, Supplementary Table 2A in ref. ^78^), mammalian myocyte secretomes (Table 1 in ref. ^79^, Supplementary Table S1 in ref. ^80^, Supplementary Table 1 in ref. ^81^, Supplementary Table 1 in ref. ^82^, Table 2 in ref. ^83^, Supplementary Table S1 in ref. ^84^, Table S5 in ref. ^85^, Supplementary Table S1 in ref. ^86^). The main text and supplementary information shows all of the data collected as part of this work. The corresponding authors will provide original data on reasonable request. Source data are provided with this paper.

## Source data

Source Data

## Material availability

The authors will make all materials (stocks, cells, plasmids) available upon request, and as indicated in this manuscript, other materials can be received from commercial and non-commercial sources.
