# Supplementary material for: Proteomics of protein trafficking by in vivo tissue-specific labeling
Source: Nat Commun. 2021 Apr 22;12:2382. doi: 10.1038/s41467-021-22599-x (PMC8062696; doi:10.1038/s41467-021-22599-x)
Supplement: Supplementary file 1 — Supplementary information [file 41467_2021_22599_MOESM1_ESM.pdf]

Supplementary Information for

Proteomics of protein trafficking by *in vivo* tissue-specific labeling

Nature Communications (2021)

Ilia A. Droujinine*, Amanda S. Meyer#, Dan Wang#, Namrata D. Udeshi#, Yanhui Hu, David Rocco, Jill A. McMahon, Rui Yang, JinJin Guo, Luye Mu, Kiki Carey, Tanya Svinkina, Rebecca Zeng, Tess Branon, Areya Tabatabai, Justin A. Bosch, John M. Asara, Alice Y. Ting, Steven A. Carr, Andrew P. McMahon, Norbert Perrimon*

* Correspondence to I.A.D. idroujinine@scripps.edu and N.P. perrimon@genetics.med.harvard.edu

This PDF file includes:

- Supplementary Discussion
- Supplementary Figs. 1-16
- Supplementary Tables 1-9
- References

Other Supplementary Files for this manuscript include the following:

- Supplementary Data 1-6
- Supplementary Movies 1-3
- Source Data
- Editorial Policy Checklist
- Reporting Summary

Supplementary Discussion

Justification of the choice of promiscuous BirA* as the labeling enzyme for proteomics of secreted protein trafficking

Before selecting BirA* as the labeling enzyme of choice for detecting proteins involved in interorgan communication, we considered a number of other options. Listed below are the various methods and the issues associated with their application to secretome analysis. In the end of this section, we describe the advantages of the biotin-ligase-based protein labeling approach.

Co-translational protein labeling approaches

Co-translational protein labeling approaches, in which labeled amino acids are incorporated into growing protein chains during translation[1-8], have not been applied to identify proteins trafficking between specific organs. These approaches have a number of drawbacks for protein trafficking experiments. First, proteins including secreted ones such as insulin can be stored intracellularly[9], with residence of a day or more[10,11] and with wide variation in translation rates[12]. Also, co-translational labeling approaches currently only label proteins throughout the cell. Hence, the subcellular region of origin (for example ER or cytoplasm) of each protein, and whether the protein is produced via a known secretory pathway, cannot be determined.

For instance, azidohomoalanine (AHA) can be pulse incorporated into cells *in vitro*, and the media can be collected for secretome analysis[1]. However, this approach has no cell specificity, so it can only be applied to single cell types in culture and not to trafficking between cell types.

Also, in trans-SILAC (stable isotope labeling of amino acids in cell culture), pre-labeled cell populations are mixed *in vitro*, which precludes long-term monitoring and *in vivo* experiments[2]. Isotopes and not affinity tags are used, making low-abundance factor detection challenging.

In a third type of co-translational protein labeling approach, cell type-specific labeling using amino acid precursors (CTAP), cells expressing different L-lysine biosynthesis enzymes are *in vitro* grown without essential amino acid L-lysine but in presence of isotope-labeled L-lysine precursors for the enzymes[3,13]. CTAP has not been performed *in vivo*, and experiments using live organisms are challenging using the method for several reasons. First, multicellular organisms need to be cultured in L-lysine-deficient media, and one L-lysine biosynthesis enzyme needs to be expressed in one organ and another enzyme in the rest of the organs. Also, label exchange has been shown to occur[3,13], during which labelled L-lysine or biosynthesis enzymes leak or exchange between cells. Moreover, CTAP uses isotope-labeled amino acids (not affinity tags)[3,13], which impedes low-abundance labeled protein detection in complex protein samples including serum and unlabeled tissues.

In a related method, NANCAT (nitrilase-activatable non-canonical amino acid tagging), nitrilase enzyme converts a precursor to an amino acid (AHA) for translating chain incorporation[4]. Like for CTAP, label retention or exchange of nitrilase or AHA is a concern which precludes *in vivo* experiments to study communication between organs.

In BONCAT (biorthogonal noncanonical amino acid tagging)[5,6] and SORT (stochastic orthogonal recoding of translation)[7,14], a modified amino acid is inserted using mutant methionyl-tRNA synthetase, phenylalanyl-tRNA synthetase or pyrrolysyl-tRNA synthetase-tRNA pair. However, these tRNAs compete with endogenous tRNAs and labeling efficiency is low at lower non-toxic levels of modified amino acids[5,7,14], which may make it challenging to detect low abundance secreted growth or signaling factors[15].

Finally, Barrett *et al* presented a method in which a puromycin analog is inserted into polypeptide chains cell-selectively due to expression of an enzyme (PGA; penicillin G acylase) that generates the puromycin analog[8]. However, insertion of a puromycin analog into polypeptide chains causes translation termination; hence, stability, secretion, or function of a protein may be affected[16].

Peroxidase (APEX/HRP)-based protein labeling approaches

Peroxidase-based (APEX/HRP) approaches have been previously used in static proximity-dependent biotinylation studies (e.g., protein-protein interactions or mitochondrial proteome)[17]. Biotinylation by APEX/HRP requires rapid harsh labeling with H_2O_2 and biotin-phenol, followed by immediate cell lysis[17]. Moreover, H_2O_2 and biotin-phenol may be difficult to take up by multi-organ organisms and dissected tissues are usually used[18] – prohibiting long-term monitoring.

Biotin-ligase (BirA*)-based protein labeling approaches

Promiscuous biotin-ligase BirA*R118G has been previously used in static proximity-dependent biotinylation studies (e.g., protein-protein interactions or subcellular proteome)[19]

We selected BirA* as the labeling agent for our approach for the following reasons. First, unlike peroxidase-based methods, BirA* labeling is mild and does not require H_2O_2/biotin-phenol, which allows following protein fate and trafficking over long-term periods *in vivo* (**Results**). Moreover, unlike co-

translational labeling approaches, BirA* can be used to label proteins within a specific subcellular compartment. In addition, unlike co-translational approaches, BirA* can be used to label stored/pre-made proteins, independent of translation rates. An additional advantage of using biotin as an affinity tag is the strong interaction between biotin and streptavidin, potentially allowing the identification of rare proteins or proteins with a small number of biotin tags[20].

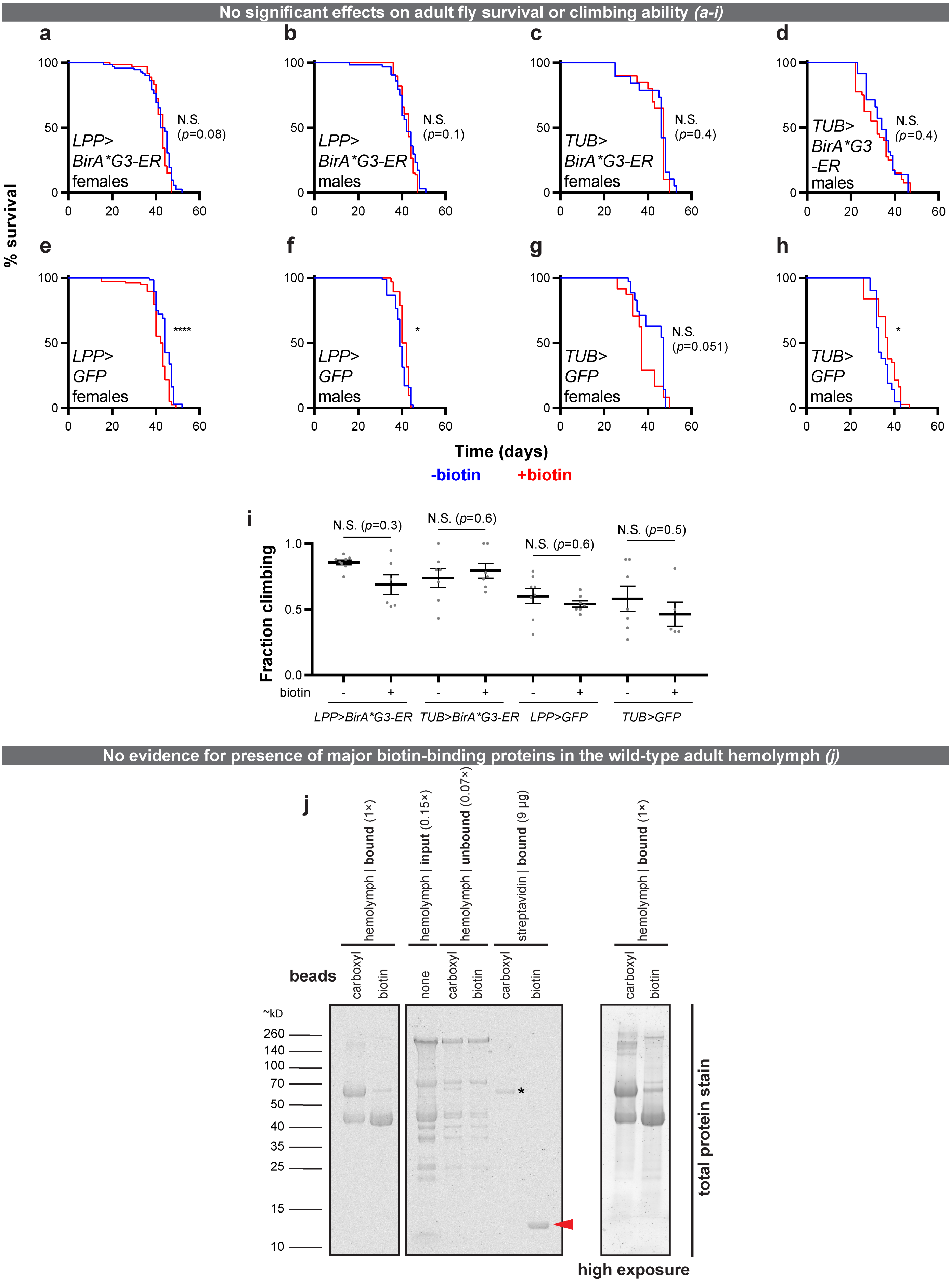

Supplementary Figure 1: Whole body or fat body (FB) biotinylation does not significantly affect adult fly survival or climbing ability, and there is no evidence for presence of major biotin-binding proteins in *wild-type (wt)* adult hemolymph (Fig. 1 supplement).

Supplementary Figure 1 (Continued).

a-h, Adults were grown on regular food and switched to regular food (-biotin, blue) or biotin-containing food (+biotin, red) at 29°C. Statistics: Log-rank test (two-tailed). N.S. means not significant. **(a)** *LPP-Gal4>BirA*G3-ER* females. –biotin: *n*=72 flies, median lifespan=43 days. +biotin: *n*=73 flies, median lifespan=43 days. N.S. (*p*=0.081). **(b)** *LPP-Gal4>BirA*G3-ER* males. –biotin: *n*=64 flies, median lifespan=42 days. +biotin: *n*=79 flies, median lifespan=43 days. N.S. (*p*=0.1035). **(c)** *TUB-Gal4>BirA*G3-ER* females. –biotin: *n*=19 flies, median lifespan=46 days. +biotin: *n*=20 flies, median lifespan=47 days. N.S. (*p*=0.43). **(d)** *TUB-Gal4>BirA*G3-ER* males. –biotin: *n*=35 flies, median lifespan=34 days. +biotin: *n*=40 flies, median lifespan=32 days. N.S. (*p*=0.52). **(e)** *LPP-Gal4>mCD8-GFP* females. –biotin: *n*=68 flies, median lifespan=44 days. +biotin: *n*=78 flies, median lifespan=42.5 days. ****p=0.000015. **(f)** *LPP-Gal4>mCD8-GFP* males. –biotin: *n*=76 flies, median lifespan=39 days. +biotin: *n*=104 flies, median lifespan=41 days. *p=0.015. **(g)** *TUB-Gal4>mCD8-GFP* females. –biotin: *n*=35 flies, median lifespan=47 days. +biotin: *n*=24 flies, median lifespan=37 days. N.S. (*p*=0.051). **(h)** *TUB-Gal4>mCD8-GFP* males. –biotin: *n*=21 flies, median lifespan=33 days. +biotin: *n*=37 flies, median lifespan=37 days. *p=0.036.

i, Climbing ability assays in 3 week old flies at 29°C. Biological replicates: *n*=8 (*LPP-Gal4>BirA*G3-ER* –biotin), *n*=6 (*LPP-Gal4>BirA*G3-ER* +biotin), *n*=7 (*TUB-Gal4>BirA*G3-ER* –biotin), *n*=7 (*TUB-Gal4>BirA*G3-ER* +biotin), *n*=8 (*LPP-Gal4>mCD8-GFP* –biotin), *n*=7 (*LPP-Gal4>mCD8-GFP* +biotin), *n*=7 (*TUB-Gal4>mCD8-GFP* –biotin), *n*=5 (*TUB-Gal4>mCD8-GFP* +biotin). Statistics: mean±SEM; one-way ANOVA and Benjamini, Krieger, Yekutieli Linear Two-Stage Step-Up FDR.

j, Equal volumes of hemolymph from *wt* adult flies were incubated with control (carboxyl) or biotin-conjugated magnetic beads, bound and unbound fractions were separated on a protein gel, and the gel was stained with sensitive flamingo staining[21] (Biorad). We observed no significant differences of proteins bound to control and biotin-conjugated beads, suggesting absence of major biotin-binding proteins in *wt* adult hemolymph. On the gel, 1×, 0.15×, and 0.07× represent how much hemolymph input equivalents by volume were run on the gel for bound, input, or unbound bead fractions. On the right is higher exposure of bound hemolymph fractions. Pull-down of streptavidin (red arrow) was used as a positive control. Asterisk is bovine serum albumin (BSA; used in bead storage buffer) that was eluted from control carboxyl beads. All lanes are from the same gel. The high exposure lanes are from a different image of the same gel (see **Source Data** for uncropped gels). This experiment was performed once.

Source data are provided as a **Source Data** file.

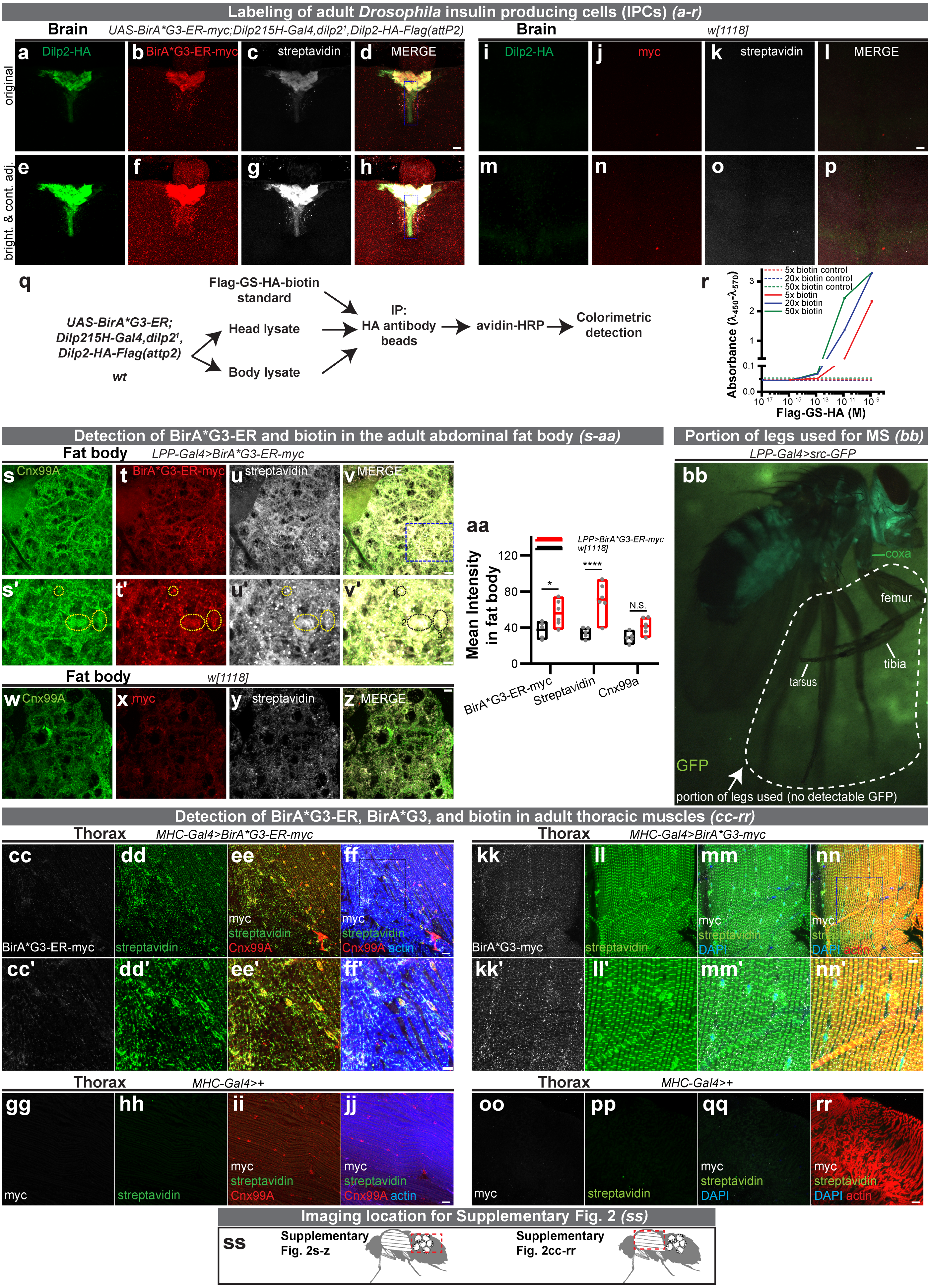

Supplementary Figure 2: Biotinylated Dilp2 (*Drosophila* insulin-like peptide 2), abdominal fat body (FB) proteins, and thoracic muscle proteins detection in adults (Fig. 1 supplement).

Supplementary Figure 2 (Continued).

In **(a-p, s-aa, cc-rr)** Flies were fed with 50 µM biotin during adulthood.

a-p, *UAS-BirA*G3-ER-myc;Dilp215H-Gal4,dilp2^1,Dilp2-HA-Flag(attP2)* **(a-h)** or *w[1118] wt* control **(i-p)** brains were stained for HA (green), myc (red), and streptavidin (white). Representative maximum intensity projections (MIPs) are shown from the examined 6 brains across 2 experimental days **(a-h)**, and 9 brains across 2 experimental brains **(i-p)**. **(a-d)** and **(i-l)** are original images, and **(e-h)** and **(m-p)** are images with equally-adjusted brightness and contrast. In **(d)** and **(h)**, the blue rectangle is the region from which **Fig. 1b** (2 confocal slices) was cropped. Scale bar: 10 µm.

q, Procedure for spatially informed bead enzyme-linked immunosorbent assay (ELISA) of biotinylated Dilp2-HA. IP = immunoprecipitation.

r, Calibration curve of synthesized Flag-GS-HA standard peptide, biotinylated with different molar excess of sulfo-NHS-biotin. Shown are means±SEM of three repeat spectrophotometric measurements of the same sample. Dashed lines are absorbances of negative control samples (PBS treated with sulfo-NHS-biotin).

s-aa, Detection of BirA*G3-ER and biotin in the adult abdominal fat body (FB). *LPP-Gal4>UAS-BirA*G3-ER-myc* **(s-v)** and *w[1118] wt* control **(w-z)** abdomen samples were stained for ER marker Cnx99A[22] (green), myc (red), and streptavidin (white). Representative MIPs are shown. **(s′-v′)** are zoomed in images from blue rectangle in **(s-v)**. Examples of areas of overlap between the Cnx99a, BirA*, and streptavidin channels are highlighted in the numbered ovals and circles in **(s′-v′)**. Scale bar: 10 µm **(s-v, w-z)**; 5 µm **(a′-d′)**. **(aa)** Quantification of mean intensity signals: myc and streptavidin signals are significantly increased in *LPP>BirA*G3-ER-myc* FBs relative to *w[1118]* controls, while Cnx99A marker was not statistically significantly different between the two samples. For each of the confocal channels, the numbers of replicates are: *n*=6 (*LPP>BirA*G3-ER-myc*) and *n*=5 (*w[1118]*) confocal images (MIPs). Statistics: mean with range of data; one-way ANOVA and Holm-Sidak's multiple comparisons test. Shown are box plots (min to max) with the line at the median. *****p*=0.000067, **p*=0.03, N.S. means not significant (*p*=0.09). See **Supplementary Figure 2ss** for the schematic of the location of imaging.

bb, Portion of legs used for mass spectrometry (MS) and western blot analyses. Genotype: *LPP-Gal4>UAS-srcGFP*. These results suggest that suggested that leg femur, tibia, and tarsus (that is, below the coxa)[23,24] do not have detectable FB or GFP expression.

cc-jj, Detection of BirA*G3-ER and biotin in the adult thoracic muscles. *MHC-Gal4>UAS-BirA*G3-ER-myc* **(cc-ff)** and *MHC-Gal4>+ wt* control **(gg-jj)** thorax samples were stained for myc (white), streptavidin (green), ER marker Cnx99A[22] (red), and actin (phalloidin; blue). Representative maximum intensity projections from 4 images across 4 thoracic samples for each genotype. **(cc′-ff′)** are zoomed in images from blue rectangle in **(cc-ff)**. Scale bar: 10 µm **(cc-ff, gg-jj)**; 5 µm **(cc′-ff′)**. See **Supplementary Figure 2ss** for the schematic of the location of imaging.

kk-rr, Detection of BirA*G3 and biotin in the adult thoracic muscles. *MHC-Gal4>UAS-BirA*G3-myc* **(kk-nn)** and *MHC-Gal4>+ wt* control **(oo-rr)** thorax samples were stained for myc (white), streptavidin (green), nuclei (DAPI; blue), and actin (phalloidin; red). Shown are representative maximum intensity projections from 2 images across 2 thoracic samples for each genotype. **(kk′-nn′)** are zoomed in images from black rectangle in **(kk-nn)**. Scale bar: 10 µm **(kk-nn, oo-rr)**; 5 µm **(kk′-nn′)**. See **Supplementary Figure 2ss** for the schematic of the location of imaging.

ss, Fly cartoon schematic of the imaging locations (red boxed areas) for the indicated figures.

Source data are provided as a **Source Data** file.

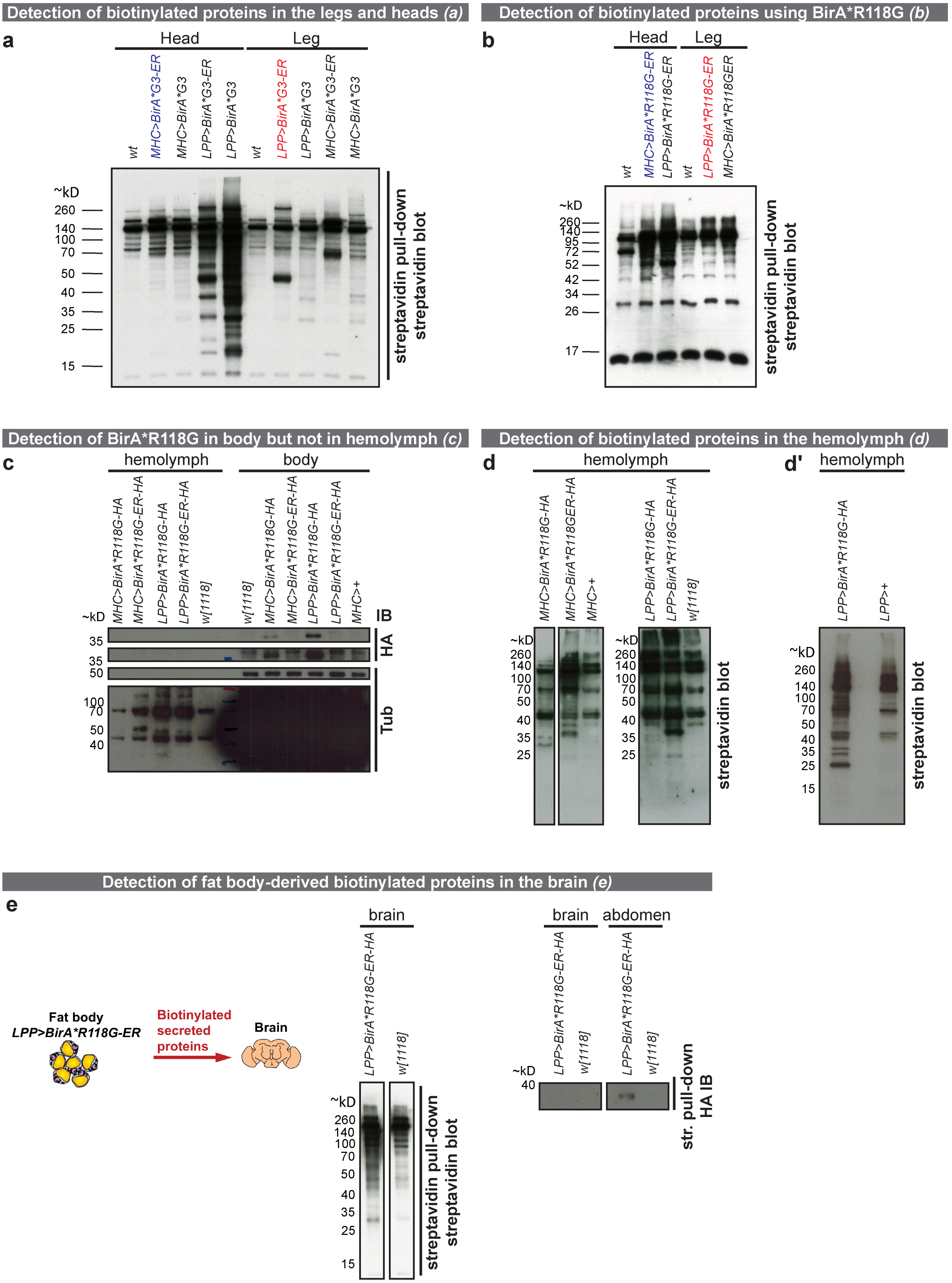

Supplementary Figure 3: Detection of biotinylated proteins in organs of origin and destination (Fig. 1 supplement)

Supplementary Figure 3 (Continued).

In all panels, flies were maintained with 50 μM biotin in food during adulthood. *wt* means *wild-type*.
For **(a-b)** See **Supplementary Figure 2bb** for the parts of legs used.

a, Detection of biotinylated proteins in the legs and heads: an additional repeat of the experiment in **Fig. 1e** with an extended set of samples. Streptavidin bead pull-down was followed by streptavidin-HRP-based detection in head and leg lysates after FB and muscle biotinylation using BirA*G3-ER or BirA*G3. *MHC>BirA*G3-ER* heads (blue text): muscle endoplasmic reticulum (ER)-derived head proteins. *MHC>BirA*G3* heads: muscle cytosol/nucleus-derived head proteins. *LPP>BirA*G3-ER* heads: head fat body (FB) ER-resident proteins. *LPP>BirA*G3* heads: head fat body (FB) cytosol/nucleus-resident proteins. *LPP>BirA*G3-ER* legs (red text): FB ER-derived leg proteins. *LPP>BirA*G3* legs: FB cytosol/nucleus-derived leg proteins. *MHC>BirA*G3-ER* legs: leg muscle ER-resident proteins. *MHC>BirA*G3* legs: leg muscle cytosol/nucleus-resident proteins. Representative result of 3 western blots (including **Fig. 1e**). All lanes are from the same blot (see **Source Data** for uncropped blots).

b-e, Organ-of origin biotinylation using BirA*R118G and detection of biotinylated proteins in distal organs or body parts.

b, Streptavidin bead pull-down followed by streptavidin-HRP detection in head and leg lysates after muscle (*MHC-Gal4*) and fat body (FB, *LPP-Gal4*) biotinylation using BirA*R118G-ER. Genotypes (males and females): *w[1118] wt*, *UAS-BirA*R118G-ER(attP40)/UAS-BirA*R118G-ER(attP40);MHC-Gal4/MHC-Gal4* and *UAS-BirA*R118G-ER(attP40)/UAS-BirA*R118G-ER(attP40);LPP-Gal4/UAS-BirA*R118G-ER(attP2)*. All lanes are from the same blot (see **Source Data** for uncropped blots). The colors of the text are for emphasis: *MHC>BirA*R118G-ER* heads (blue text) and *LPP>BirA*R118G-ER* legs (red text). This western blot was run once, however, flies of these genotypes were used in the proteomics experiment in **Supplementary Figure 7**.

c, BirA*R118G-HA or BirA*R118G-ER-HA is detected in body but not hemolymph (blood). For HA and Tubulin (Tub), lower (top panel) and higher (bottom panel) exposures are shown. Note that the body BirA*R118G-ER-HA is detected at lower levels than BirA*R118G (cytoplasmic/nuclear); however, this signal is consistently detected in multiple experiments. Genotypes (females): *w[1118] wt*, *UAS-BirA*R118G(attp40)/UAS-BirA*R118G(attP40);MHC-Gal4/MHC-Gal4*, *UAS-BirA*R118G-ER(attP40)/UAS-BirA*R118G-ER(attP40);MHC-Gal4/MHC-Gal4, UAS-BirA*R118G(attP40)/UAS-BirA*R118G(attP40);LPP-Gal4/LPP-Gal4*, *UAS-BirA*R118G-ER(attP40)/UAS-BirA*R118G-ER(attP40);LPP-Gal4/LPP-Gal4*, *+/+;MHC-Gal4*. IB means immunoblot. The body HA detection was performed at least twice, while hemolymph HA detection was performed once. For each slice, all lanes are from the same blot (see **Source Data** for uncropped blots).

d, Detection of biotinylated proteins in the hemolymph after muscle and FB biotinylation using BirA*R118G-ER and BirA*R118G (cytoplasmic/nuclear biotinylation for unconventional protein secretion). Genotypes (females): *w[1118] wt*, *UAS-BirA*R118G(attP40)/UAS-BirA*R118G(attP40);MHC-Gal4/MHC-Gal4*, *UAS-BirA*R118G-ER(attP40)/UAS-BirA*R118G-ER(attP40);MHC-Gal4/MHC-Gal4, UAS-BirA*R118G(attP40)/UAS-BirA*R118G(attP40);LPP-Gal4/LPP-Gal4*, *UAS-BirA*R118G-ER(attP40)/UAS-BirA*R118G-ER(attP40);LPP-Gal4/LPP-Gal4*, *+/+;MHC-Gal4*. In **(d')** (different experiment than **(d)**), additional controls for *LPP-Gal4* are shown. Genotypes: *LPP-Gal4>+* and *LPP-Gal4>UAS-BirA*R118G.* For the *MHC>* hemolymph sub-figure (**d**, left), all lanes are from the same blot (see **Source Data** for uncropped blots). For the *LPP>* hemolymph sub-figure (**d**, right), all lanes are from the same blot (see **Source Data** for uncropped blots). For the *LPP>* hemolymph figure (**d'**), all lanes are from the same blot (see **Source Data** for uncropped blots). The *UAS-BirA*R118G-ER(attP40)/UAS-BirA*R118G-ER(attP40);LPP-Gal4/LPP-Gal4* blood experiment was repeated another time. The experiment using hemolymph from *LPP-Gal4>UAS-BirA*R118G-ER* and *MHC-Gal4>UAS-BirA*R118G-ER* was performed at least an additional time. The experiment similar to **(d')** was performed additional two times.

Supplementary Figure 3 (Continued).

e, Abdomens and FB-free brains were dissected from *w[1118] wt* and *UAS-BirA*R118G-ER(attP40)/UAS-BirA*R118G-ER(attP40);LPP-Gal4/UAS-BirA*R118G-ER(attP2)* male adult flies. Blots for streptavidin-HRP (left) and HA (right) were performed. Extra biotinylated proteins were detected in brains of *LPP>BirA*R118G-ER* flies, while HA is detected in abdomens of *LPP>BirA*R118G-ER* flies only. For the brain streptavidin pulldown, streptavidin blot sub-figure (left), all lanes are from the same blot (see **Source Data** for uncropped blots). The streptavidin western blot was repeated once using *UAS-BirA*R118G-ER-HA(attP40)/+;LPP-Gal4/+*. For the streptavidin pulldown, HA IB sub-figure (right), all lanes are from the same blot (see **Source Data** for uncropped blots).

Source data are provided as a **Source Data** file.

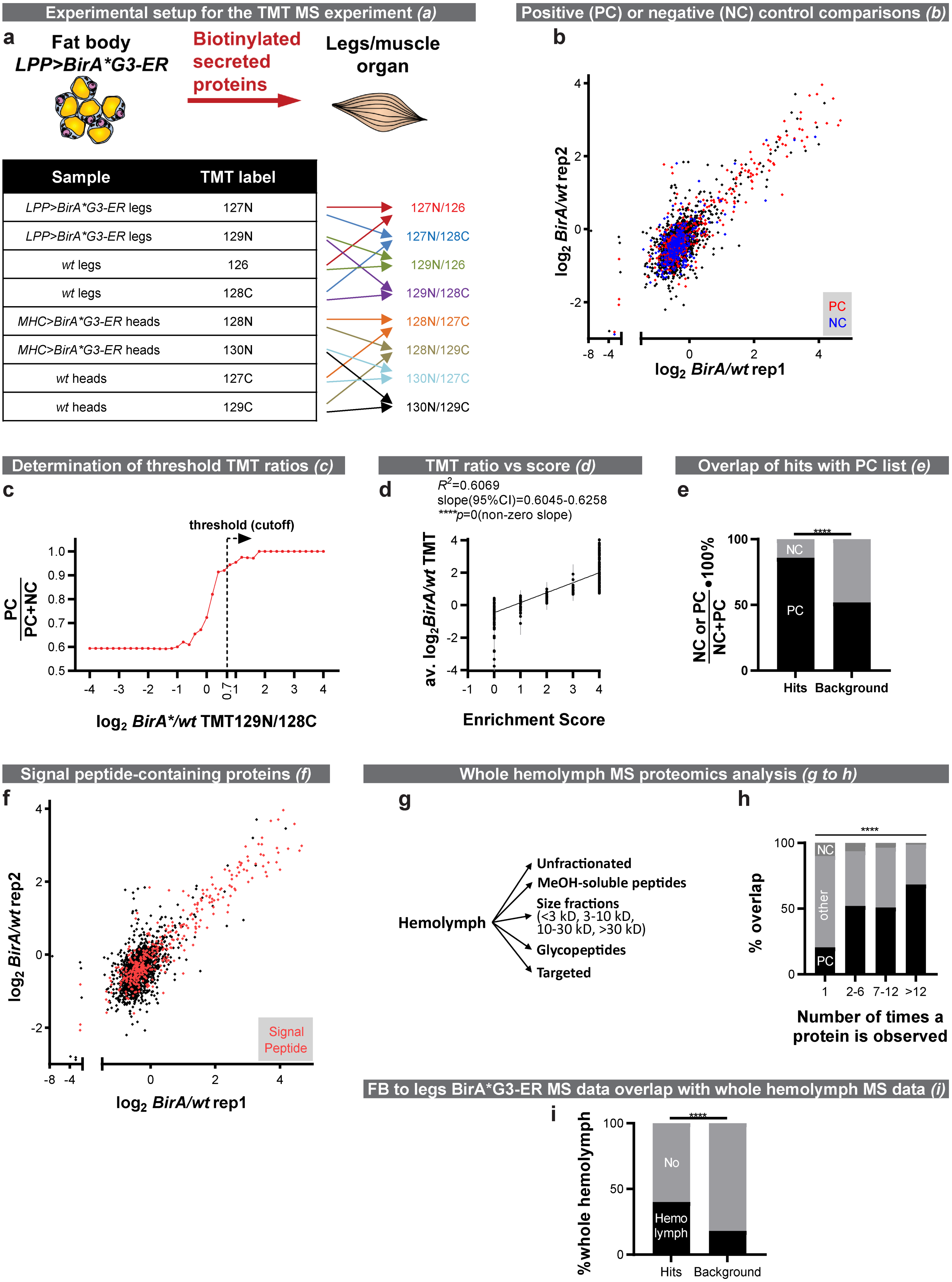

Supplementary Figure 4: Thresholding and signal peptide analyses of biotinylated leg/muscle organ proteins originating from fat body (FB) tandem mass-tag (TMT) mass spectrometry (MS) using BirA*G3-ER labeling (Fig. 2 supplement).

Supplementary Figure 4 (Continued).

a, Experimental setup for the identification of FB-derived proteins in legs and muscle-derived proteins in heads in a single quantitative TMT MS experiment. The different TMT state signals were compared to generate TMT ratios (right of the arrows). There were 4 TMT ratio comparisons for legs and 4 for heads. Genotypes: *w[1118] wt (wild-type)*, *LPP-Gal4>UAS-BirA*G3-ER*, *MHC-Gal4>UAS-BirA*G3-ER*. Flies were maintained with 50 μM biotin in food during adulthood. See **Supplementary Figure 2bb** for the parts of legs used for MS experiments.

b, Leg $\log_2$(*BirA*G3-ER / wt*) TMT-ratios in two replicates, mean $\log_2$TMT ratio (each point is *n*=2 comparisons). Proteins identified with MS were compared to positive control (PC) secreted protein/receptor (red points) and negative control (NC, intracellular) (blue points).

c, Representative #PC/(#PC+#NC) versus *BirA*G3-ER/wt* TMT ratio graph (out of four). The threshold TMT ratio was chosen at which #PC/(#PC+#NC)>0.9.

d, Increased TMT-ratios are associated with higher enrichment scores (E-S). Each point is a mean±SEM $\log_2$TMT ratio for each identified protein. *n*=107 (E-S=4), *n*=16 (E-S=3), *n*=51 (E-S=2), *n*=95 (E-S=1), and *n*=1804 (E-S=0) proteins. Linear regression results of a two-tailed F-test are presented.

e, Hits (score≥1) have higher #PC/(#PC+#NC). Statistics: Two-sided chi-squared test. ****$p=9.26\cdot10^{-15}$.

f, Proteins with an identified signal peptide[25] (red) were mapped onto the leg $\log_2$(*BirA*G3-ER/wt*) TMT-ratios in two replicates graph. Each point is *n*=2 comparisons, mean $\log_2$TMT ratio.

g-i, Whole hemolymph MS proteomics analysis of legs/muscle organ proteins derived from fat body (FB) identified in the BirA*G3-ER MS dataset. **(g)** Summary of the hemolymph processing in total hemolymph MS proteomics experiments (see the **Methods** section for more details). **(h)** Each identified protein in total fly hemolymph MS was assigned to a positive control (PC) secreted/receptor, negative control (NC) intracellular, or other (unknown) categories (see **Methods**). The x-axis is the number of times a protein is observed (or sum of unique peptides across all experiments). We identified a total of 1561 proteins, including 688 proteins of poorly-characterized functions ("Computed Genes"/CGs; FlyBase[26] r551). Statistics: Chi-squared test. ****$p=3.48\cdot10^{-9}$. The contingency table had three outcomes (PC, other, or NC) as well as 4 groups, and one- or two-sided nature of the test was not applicable. **(i)** Hits (score≥1) from the legs/muscles proteins derived from FB (identified in the BirA*G3-ER MS dataset) are enriched for proteins identified in whole fly hemolymph. Statistics: Two-sided chi-squared test. ****$p=9.79\cdot10^{-17}$.

Source data are provided as a **Source Data** file.

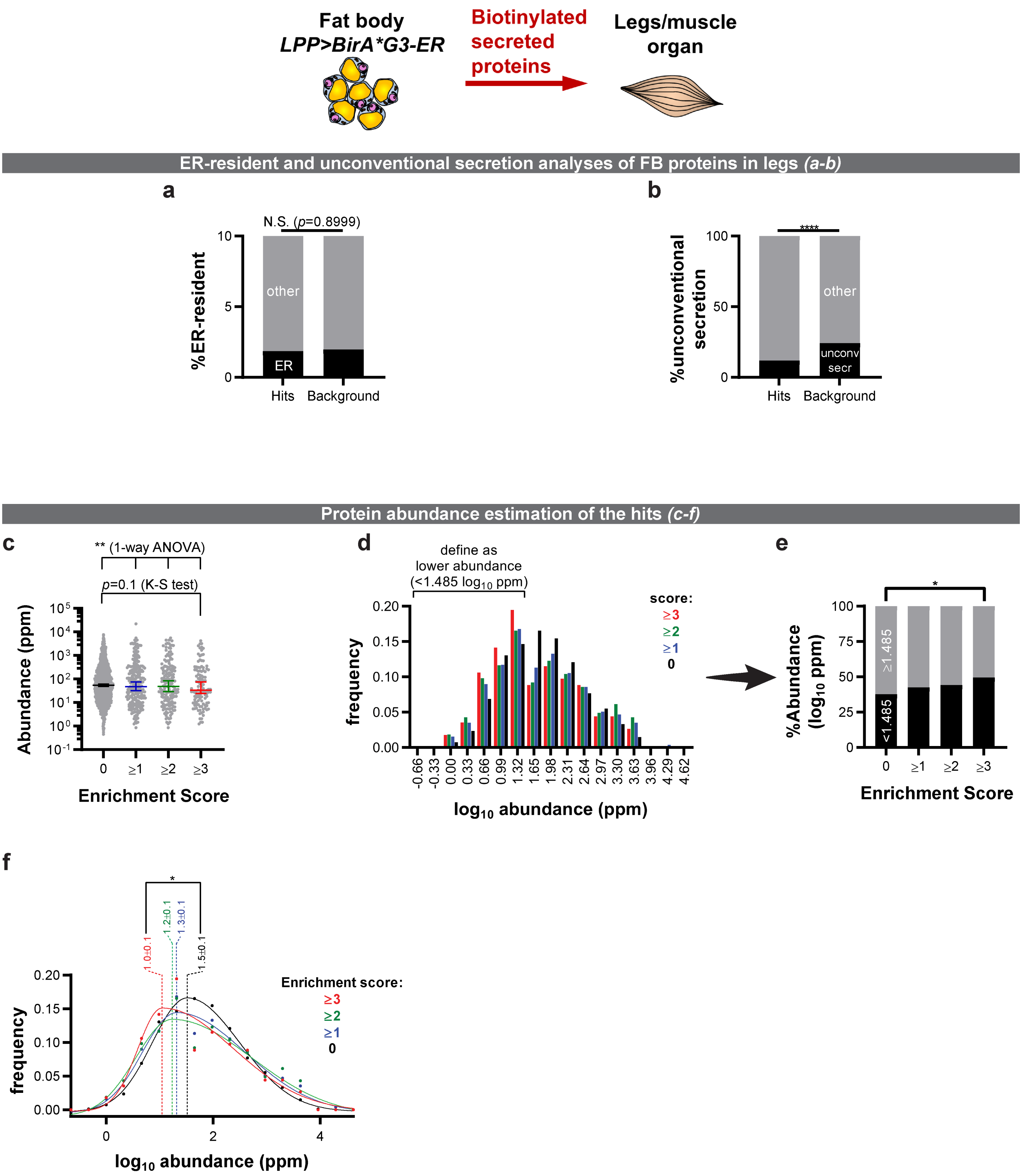

Supplementary Figure 5: ER-resident, unconventional secretion, and protein abundance estimation analyses of fat body (FB)-derived proteins present in legs/muscle organ, as identified using tandem mass-tag (TMT) mass spectrometry (MS) using BirA*G3-ER labeling (Fig. 2 supplement).

a, Hits (enrichment score (E-S) ≥1) are not enriched for ER-resident proteins. Statistics: Two-sided chi-squared test. N.S. means not significant.

b, Hits (E-S≥1) are de-enriched for proteins predicted to be secreted unconventionally[27]. Statistics: Two-sided chi-squared test. ****p=0.000006.

Supplementary Figure 5 (Continued).

c-f, BirA*G3-ER legs/muscle organ FB-derived hits were enriched for lower-abundance proteins. Protein abundance information was from integrated entire organism PAX database for *Drosophila melanogaster*[28]. Each E-S is colored in the figure.

c, Increased E-S show a trend for decreased protein abundance. Median with 95% confidence interval. **p=0.0033 (one-way ANOVA between all groups); p=0.10 (Kolmogorov-Smirnov (K-S) test between E-S=0 and E-S≥3; two-tailed p value). Each dot in the plot represents an abundance of a protein within each E-S category: n(E-S=0)=1754, n(E-S≥1)=256, n(E-S≥2)=163, n(E-S≥3)=113 individual protein abundance values.

d, Frequency vs $\log_{10}$ protein abundance plot.

e, Histogram of abundance versus score, when lower abundance is <1.485 $\log_{10}$ ppm, reveals a general trend towards lower protein abundances at higher MS scores. Two-sided chi-squared test: *p=0.013 between E-S=0 and E-S≥3.

f, Frequency vs $\log_{10}$ protein abundance plot with Bigaussian fits. Score=0: x_c (peak center±SEM)=1.5±0.1 $\log_{10}$ppm; R^2=0.9925. E-S≥1 x_c=1.3±0.1 $\log_{10}$ppm; R^2=0.96039. E-S≥2 x_c=1.2±0.1 $\log_{10}$ppm; R^2=0.91765. E-S≥3 x_c=1.0±0.1 $\log_{10}$ppm; R^2=0.90396. x_c(E-S=0) versus x_c(E-S≥3): F-test *p=0.032 (two-tailed); AIC (Akaike's Information Criterion Test) weights suggest that x_c are different (same=0.24884, different=0.75116).

Source data are provided as a **Source Data** file.

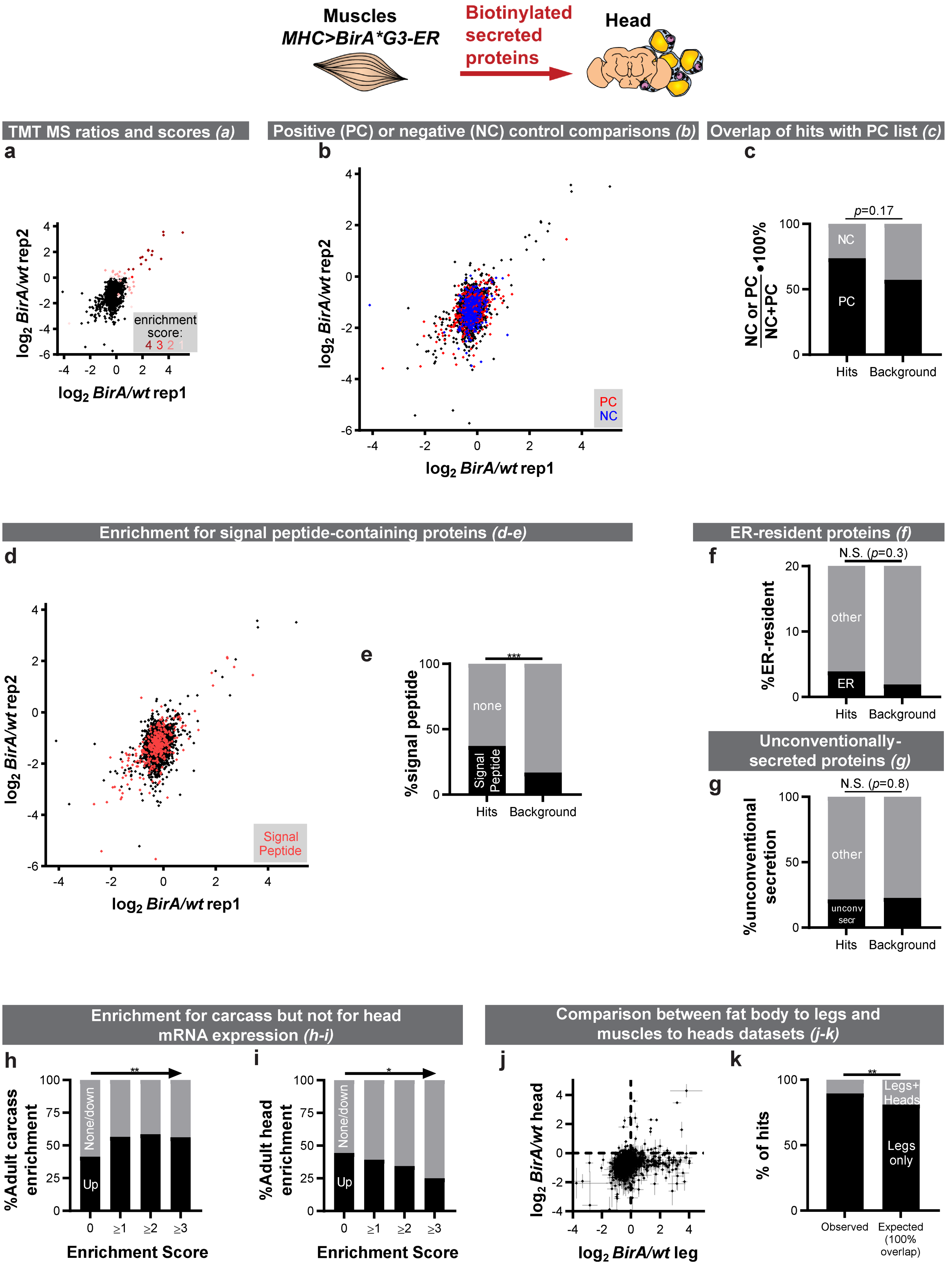

Supplementary Figure 6: Identification of muscle proteins present in heads using BirA*G3-ER (Fig. 2 supplement).

Supplementary Figure 6 (Continued).

Muscles were labeled using *MHC-Gal4>BirA*G3-ER* (endoplasmic reticulum) and biotinylated proteins from heads were analyzed using tandem mass-tag (TMT) mass spectrometry (MS). *w[1118] wt (wild-type)* heads were used as controls. Flies were maintained with 50 μM biotin in food during adulthood. The *Drosophila* head contains fat body (see **Supplementary Figure 2bb**).

a, Head log_2(*BirA*G3-ER/wt*) TMT ratios in two replicates. Each point is *n*=2 comparisons, mean log_2TMT ratio. Enrichment score (E-S): number of comparisons (from 4) in which TMT ratio>threshold (score 4 is for most confident hits and 0 is background). For each of the four *BirA*G3-ER/wt* TMT-ratio comparisons, we determined threshold TMT ratios for hit-calling as described in the **Methods** section. A total of 51 muscle endoplasmic reticulum (ER)-derived proteins targeting heads were identified.

b, Head proteins identified with MS were compared to positive control (PC) secreted protein/receptor (red points) and intracellular negative control (NC) (blue points). Each point is *n*=2 comparisons, mean log_2TMT ratio.

c, Hits (E-S≥1) trend towards higher #PC/(#PC+#NC).

d, Proteins with an identified signal peptide[25] (red) were mapped onto the head log_2(*BirA*G3-ER/wt*) TMT-ratios in two replicates graph. Each point is *n*=2 comparisons, mean log_2TMT ratio.

e, Hits (E-S≥1) have a higher fraction of proteins with putative signal peptides (see **Methods**). ****p*=0.0001.

f, Hits (E-S≥1) are not statistically-significantly enriched for ER-resident proteins.

g, Hits (E-S≥1) are not enriched for proteins predicted to be secreted unconventionally[27].

Statistics **(c, e-g)**: Two-sided chi-squared test.

h-i, Higher E-S correlate with a higher fraction of proteins enriched for adult carcass (which includes muscles) mRNA microarray[29] **(h)**, and with a lower fraction of proteins enriched for adult head mRNA microarray[29] **(i)**. Chi-squared test for trend: ***p*=0.0063, **p*=0.043. Note that this was a chi-squared test for trend, and its one-sided or two-sided nature is not applicable.

j-k, Comparison between BirA*G3-ER legs and heads datasets reveals limited overlap, suggestive of organ-specificity in secretome trafficking. **(j)** Head log_2(*BirA*G3-ER/wt*) TMT ratios vs leg log_2(*BirA*G3-ER/wt*) TMT ratios. Each point is *n*=4 comparisons; mean±SEM log_2TMT ratio. **(k)** Comparison between observed and expected (based on 100% overlap) overlap between legs and heads datasets. The observed overlap is less than expected. Two-sided chi-squared test: ***p*=0.0051.

Source data are provided as a **Source Data** file.

Experimental design *(a)*

a

Sample	TMT label
*LPP>BirA*R118G-ER* legs	127N
*LPP>BirA*R118G-ER* legs	129C
wt legs	127C
wt legs	129N
*MHC>BirA*R118G-ER* heads	128C
*MHC>BirA*R118G-ER* heads	130C
wt heads	128N
wt heads	130N

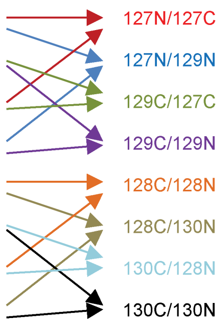

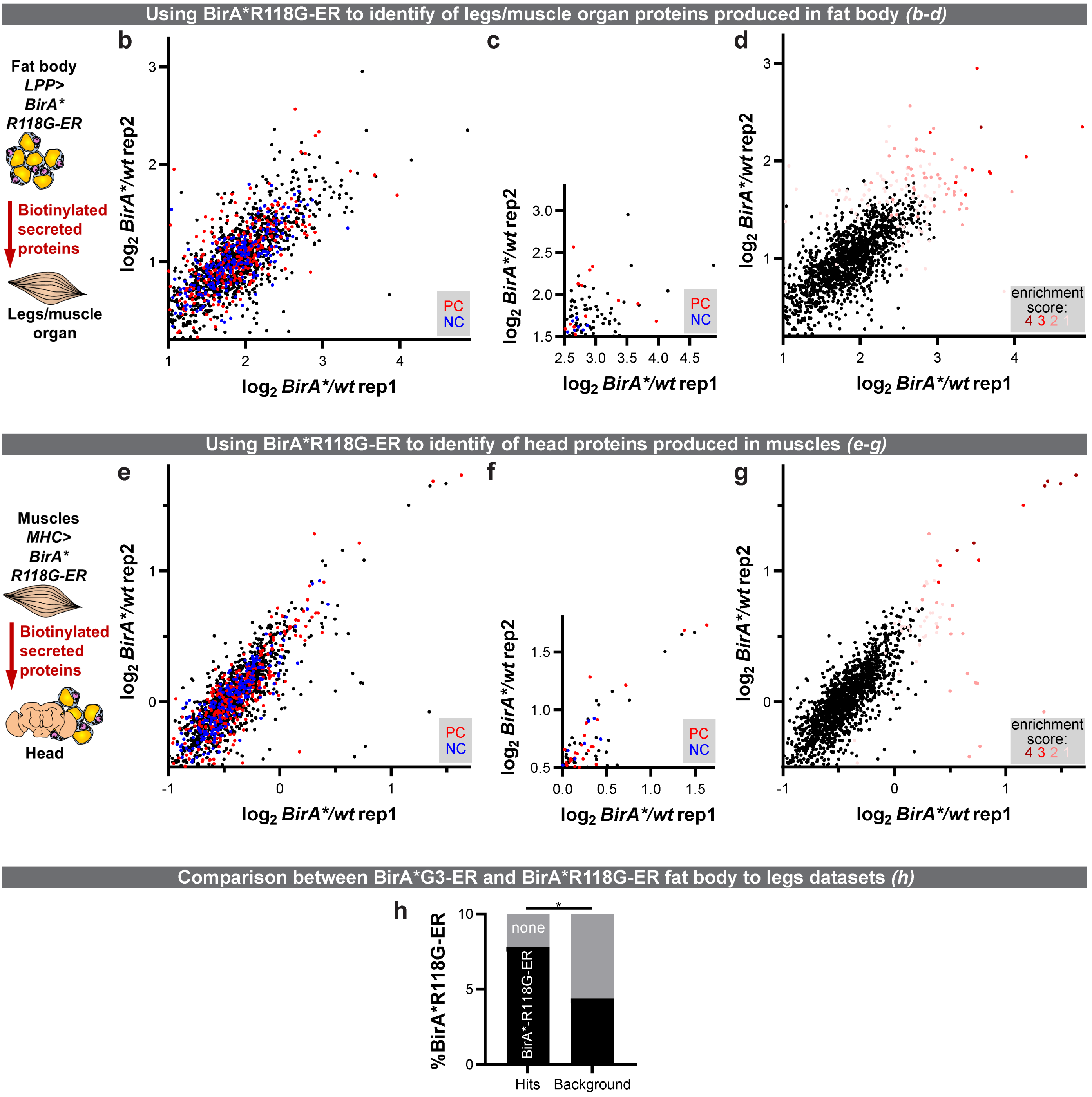

Supplementary Figure 7: Using BirA*R118G-ER to identify legs/muscle organ proteins produced from fat body (FB) and head proteins produced from muscles (Fig. 2 supplement).

Supplementary Figure 7 (Continued).

a, Experimental design. The different TMT state signals were compared to generate TMT ratios (right of the arrows). There were 4 TMT ratio comparisons for legs and 4 for heads. Genotypes: *w[1118] wt (wild-type)*, *UAS-BirA*R118G-ER(attP40)/UAS-BirA*R118G-ER(attP40);MHC-Gal4/MHC-Gal4* and *UAS-BirA*R118G-ER(attP40)/UAS-BirA*R118G-ER(attP40);LPP-Gal4/UAS-BirA*R118G-ER(attP2)*. Flies were maintained with 50 μM biotin in food during adulthood. See **Supplementary Figure 2bb** for the parts of legs used for MS experiments.

b-d, Identification of legs/muscle organ proteins produced in FB. **(b)** Leg $\log_2$(*BirA*R118G-ER/wt*) TMT ratios in two replicates. Each point is *n*=2 comparisons, mean $\log_2$TMT ratio. Leg proteins identified with MS were compared to positive control (PC) secreted protein/receptor (red points) and intracellular negative control (NC) (blue points). **(c)** Zoom of graph in **(b)**. **(d)** Leg $\log_2$(*BirA*R118G-ER/wt*) TMT ratios in two replicates. Each point is *n*=2 comparisons, mean $\log_2$TMT ratio. Enrichment score (E-S): number of comparisons (from 4) in which TMT ratio>threshold (E-S=4 is for most confident hits and 0 is background). For each of the four *BirA*R118G-ER/wt* TMT-ratio comparisons, we determined threshold TMT ratios for hit-calling, as described in the **Methods** section.

e-g, Identification of head proteins produced in muscles. **(e)** Head $\log_2$(*BirA*R118G-ER/wt*) TMT ratios in two replicates. Each point is *n*=2 comparisons, mean $\log_2$TMT ratio. Head proteins identified with MS were compared to PC and NC lists. **(f)** Zoom of graph in **(e)**. **(g)** Head $\log_2$(*BirA*R118G-ER/wt*) TMT ratios in two replicates. Each point is *n*=2 comparisons, mean $\log_2$TMT ratio. E-S: number of comparisons (from 4) in which TMT ratio>threshold (E-S=4 is for most confident hits and 0 is background). For each of the four *BirA*R118G-ER/wt* TMT-ratio comparisons, we determined threshold TMT ratios for hit-calling, as described in the **Methods** section.

h, Legs *LPP-Gal4>BirA*G3-ER* dataset hits (E-S ≥ 1) show statistically-significant overlap with *LPP-Gal4>BirA*R118G-ER* dataset hits, compared to background. Two-sided chi-squared test: *p=0.0144.

Source data are provided as a **Source Data** file.

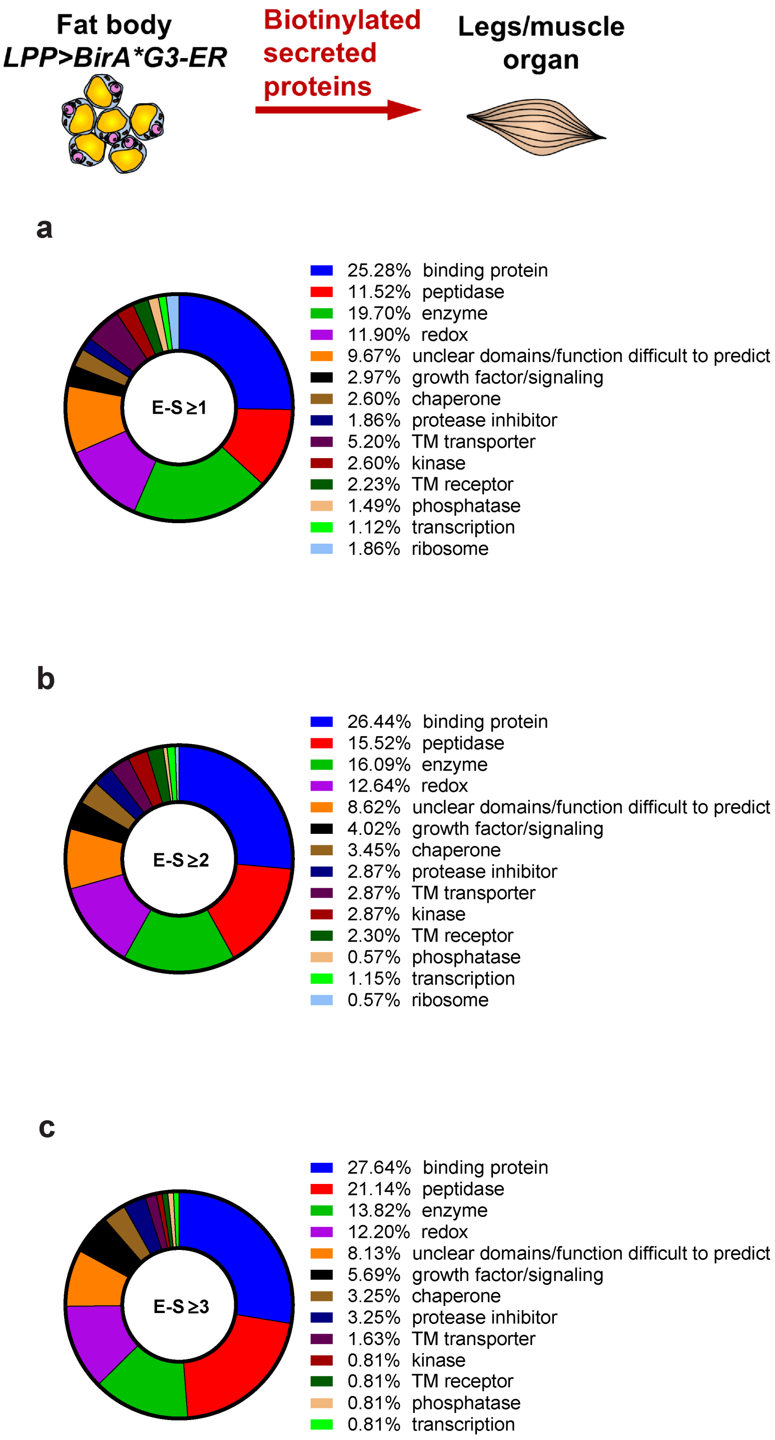

Supplementary Figure 8: Manual categorization of hits (enrichment score (E-S) ≥ 1) according to data from FlyBase[26] and ortholog data from NCBI Gene[30] (Fig. 2 supplement).

Source data are provided as a **Source Data** file.

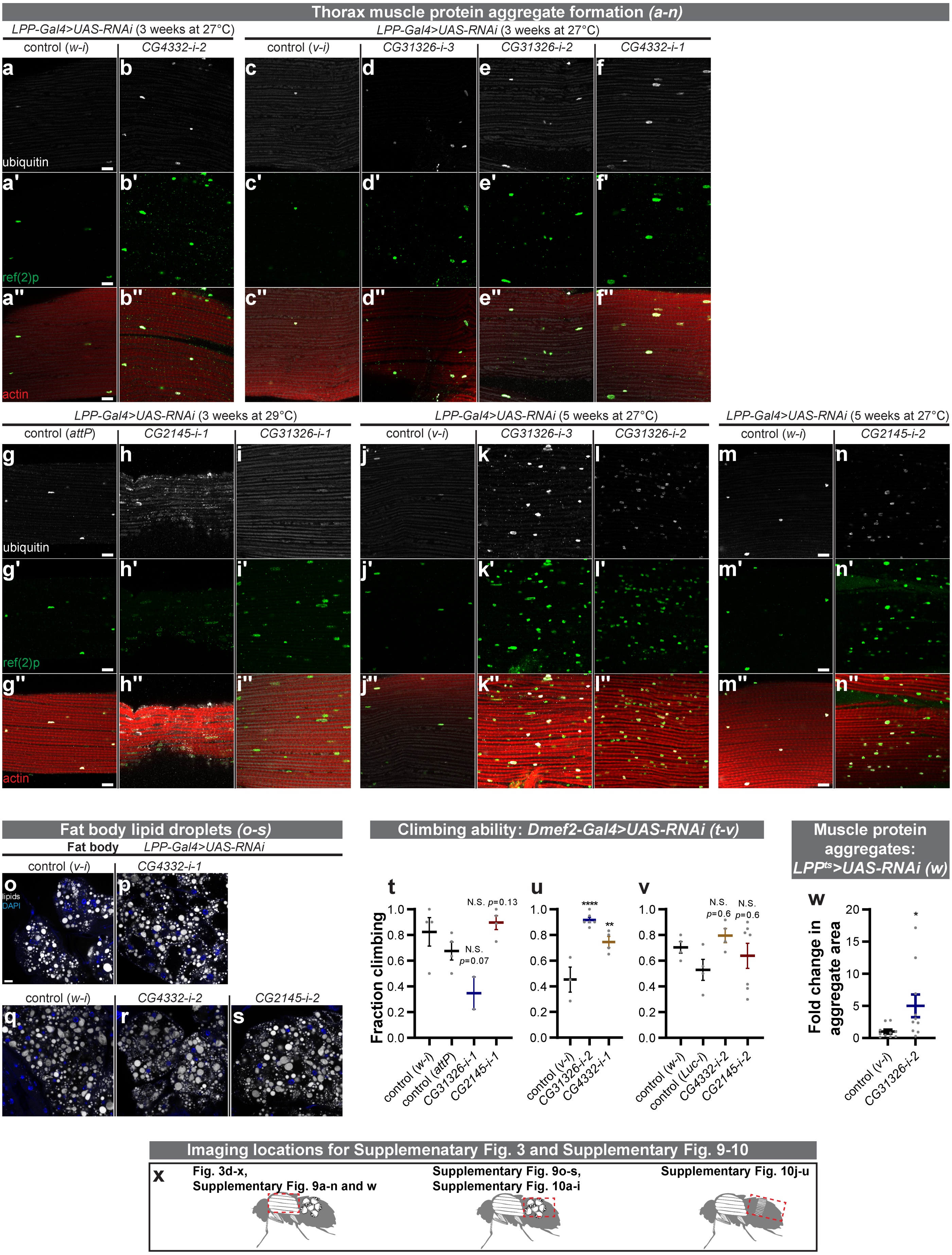

Supplementary Figure 9: Adult flies with fat body (FB) RNAi (using *LPP-Gal4*) against *CG4332*, *CG2145*, and *CG31326* have increased muscle protein aggregate formation and do not have significant defects in FB lipid droplets (Fig. 3 supplement).

Supplementary Figure 9 (Continued).

a-n, Shown are representative single confocal slices (cropped to equal extent for ease of visualization). Muscles were stained for ubiquitin (white), p62/ref(2)p (green), and actin (phalloidin, red). Quantification shown in **Fig. 3d-h** was performed from these samples (using uncropped images). Scale bar: 10 µm. **(a-b)** *LPP-Gal4>RNAi* (TRiP, Harvard Transgenic RNAi Project), 3 weeks old at 27°C. **(c-f)** *LPP-Gal4>RNAi* (NIG, Japan National Institute of Genetics), 3 weeks old at 27°C. **(g-i)** *LPP-Gal4>RNAi* (VDRC, Vienna *Drosophila* Resource Center), 3 weeks old at 29°C. **(j-l)** *LPP-Gal4>RNAi* (NIG), 5 weeks old at 27°C. **(m-n)** *LPP-Gal4>RNAi* (TRiP), 5 weeks old at 27°C. For these experiments, around 5-6 animals were dissected, and thorax was cut into 2 or 4 fragments. One (occasionally two) confocal image per fragment piece was taken, representing at least 3 animals (see **Methods – Immunostaining and confocal microscopy** for details). Samples were stained and imaged across multiple experimental days. Representative images shown from *n* total images analyzed: **(a-b)** *n*=6 (*w-i*), *n*=6 (*CG4332-i-2*). **(c-f)** *n*=6 (*v-i*), *n*=9 (*CG31326-i-3*), *n*=7 (*CG31326-i-2*), *n*=10 (*CG4332-i-1*). **(g-i)** *n*=9 (*attP*), *n*=5 (*CG2145-i-1*), *n*=7 (*CG31326-i-1*). **(j-l)** *n*=7 (*v-i*), *n*=5 (*CG31326-i-3*), *n*=6 (*CG31326-i-2*). **(m-n)** *n*=4 (*w-i*), *n*=6 (*CG2145-i-2*).

o-s, FBs were stained for lipids (BODIPY, white) and DAPI (blue). Samples were processed across multiple experimental days. Scale bar: 10 µm. **(o-p)** *LPP-Gal4>RNAi* (NIG), 3 weeks old at 27°C. **(q-s)** *LPP-Gal4>RNAi* (TRiP), 3 weeks old at 27°C. Representative single confocal slices are shown from 9 (o-r) or 10 (s) images.

t-v, Adult flies with muscle RNAi (using *Dmef2-Gal4*) against *CG4332*, *CG2145*, or *CG31326* do not have significant defects in climbing ability. Statistics: mean±SEM; one-way ANOVA and Benjamini, Krieger, Yekutieli Linear Two-Stage Step-Up FDR. **(t)** *Dmef2-Gal4>RNAi* from VDRC (Vienna *Drosophila* Resource Center) at 3 weeks old and 29°C. Biological replicates: *n*=4 (*w-i*), *n*=4 (*attP*), *n*=2 (*CG31326-i-1*), *n*=4 (*CG2145-i-1*). *p*-values are relative to control (*attP*). N.S. means not significant. **(u)** *Dmef2-Gal4>RNAi* from NIG (Japan National Institute of Genetics) at 5 weeks old and 27°C. Biological replicates: *n*=4 (*v-i*), *n*=6 (*CG31326-i-2*), *n*=4 (*CG4332-i-1*). *p*-values are relative to control (*v-i*). ****p=0.000006, **p=0.0010. **(v)** *Dmef2-Gal4>RNAi* from TRiP (Harvard Transgenic RNAi Project) at 5 weeks old and 27°C. Biological replicates: *n*=4 (*w-i*), *n*=4 (*Luc-i*), *n*=4 (*CG4332-i-2*), *n*=7 (*CG2145-i-2*). *p*-values are relative to control (*w-i*).

w, RNAi activation specifically in adults. Flies were grown at 18°C (RNAi off), and shifted to 29°C (RNAi activated) in adulthood for 3 weeks. Area of p62/ref(2)p-positive protein aggregates was quantified, and normalized and compared to controls. Genotypes: *Tub-gal80*^{ts}*;LPP-Gal4>UAS-RNAi* (NIG). Biological replicates: *n*=10. Statistics: mean±SEM; two-tailed t-test. *p=0.0374.

x, Fly cartoon schematic of the imaging locations (red boxed areas) for the indicated figures.

Source data are provided as a **Source Data** file.

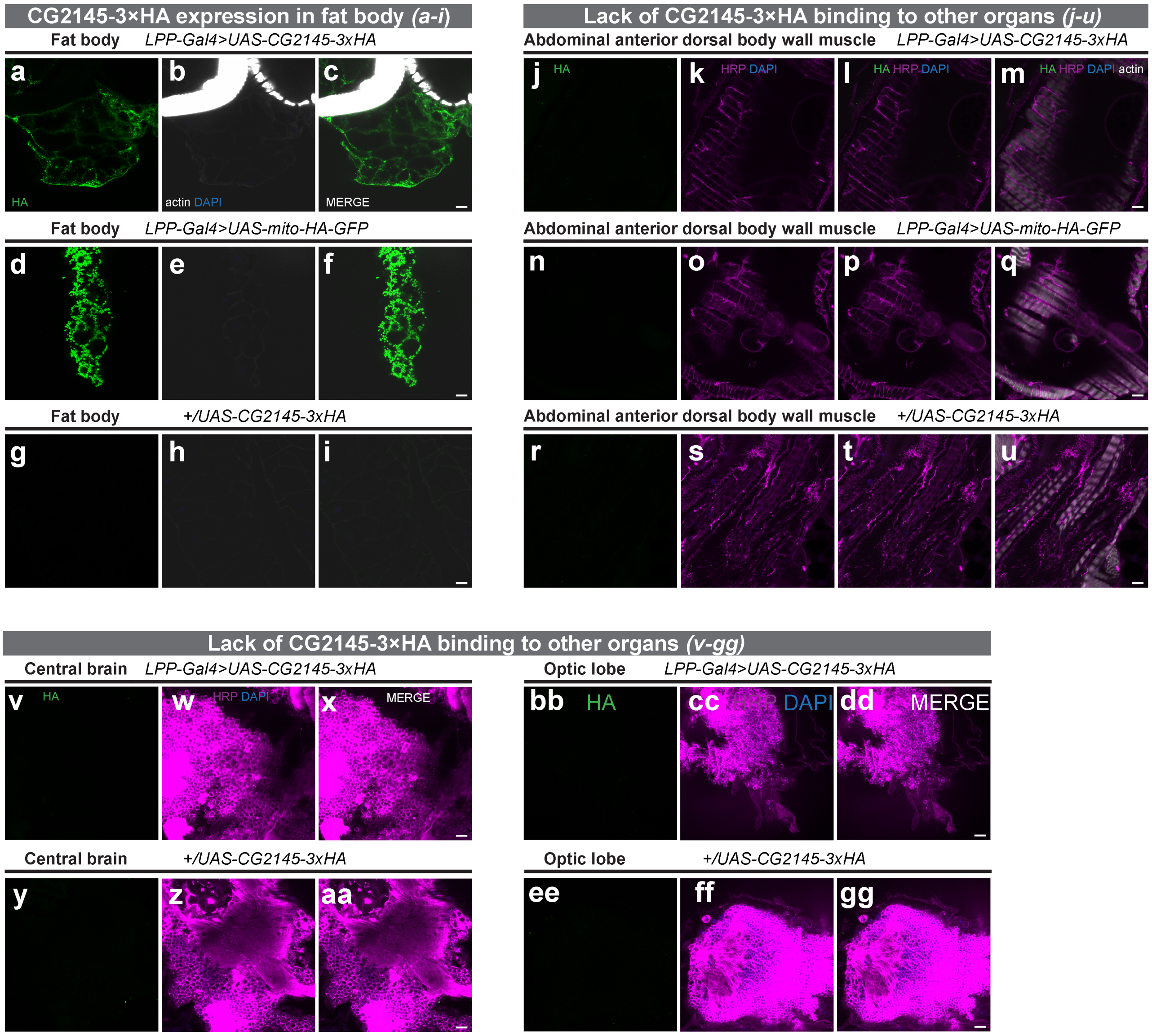

Supplementary Figure 10: CG2145 does not bind to abdominal body wall muscles, central brain, or optic lobe (Fig. 3 supplement).

Organs from indicated genotypes were stained for HA (green), neurons (HRP; magenta), nuclei (DAPI; blue), and actin (phalloidin; white). Representative maximum intensity projections are shown (same number of slices as **Fig. 4e-l**): one image each was acquired for *LPP-Gal4>UAS-CG2145-3xHA* from 4 and 3 observed abdomens and brains, respectively; for the other genotypes, one image of each organ was acquired. For fat body (FB) samples in **(b, c, e, f, h, and i)**, the actin channel was adjusted for brightness and contrast equally across images and within each image to visualize FB cell borders. Strong expression of FB-expressed HA is observed in FB (since *UAS-CG2145* is expressed in FB) of *LPP-Gal4>UAS-CG2145-3xHA* and *LPP-Gal4>UAS-mito-HA-GFP* samples. Scale bar: 10 µm.

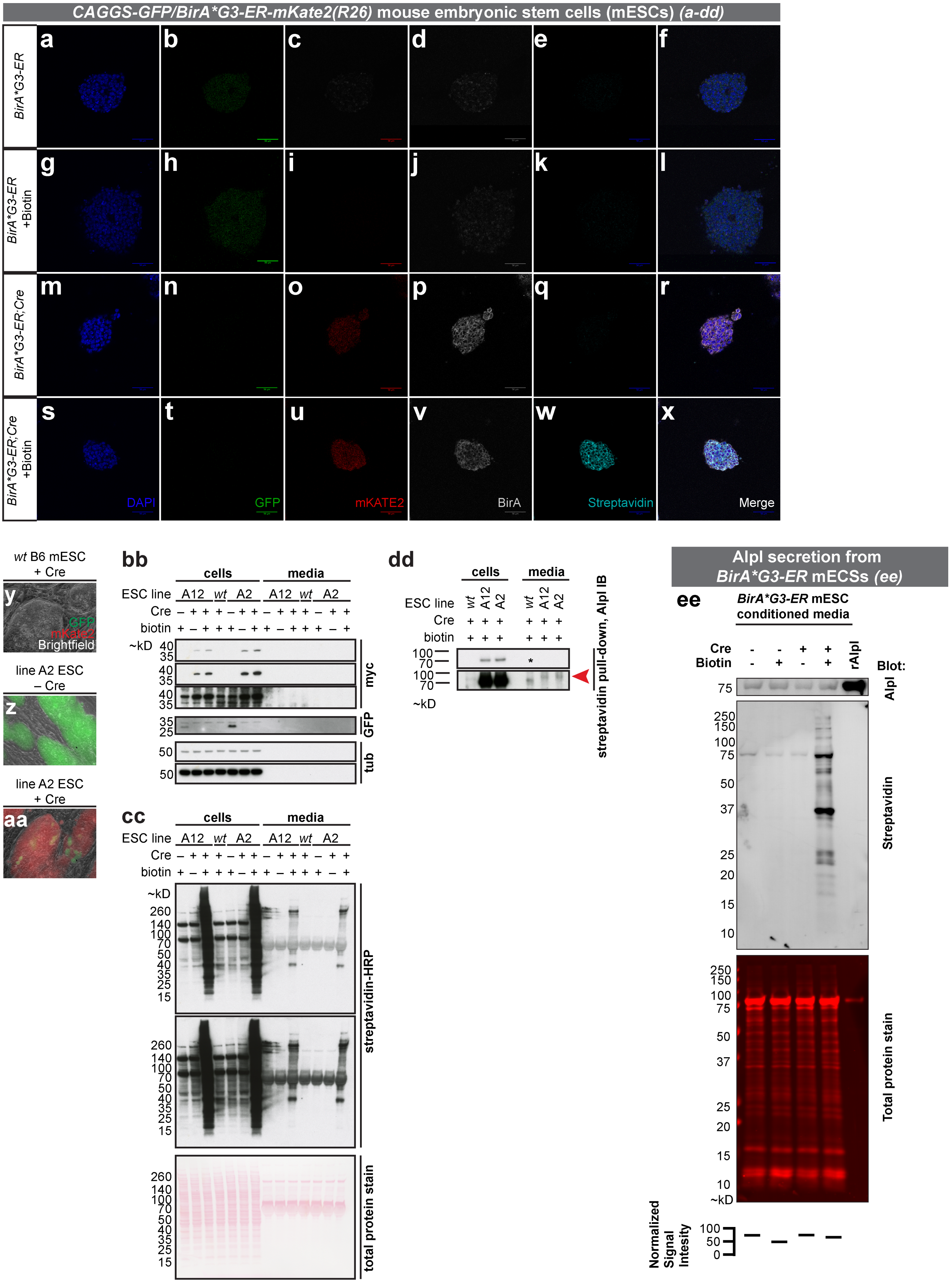

Supplementary Figure 11: mESC streptavidin and BirA expression characterization, and positive control Alpl secretion analysis (Fig. 4 supplement).

Supplementary Figure 11 (Continued).

a-x, Immunofluorescent staining of mESC colonies from *BirA*G3-ER* (B1 parent line) or *BirA*G3-ER;Cre* (Cre recombined 3A line) allele reporter expression of mKate2 (red) or GFP (green) with BirA (white) and streptavidin (cyan) immunostaining. Representative results from four repeats. Scale bar: 50 µm.

y-dd, Additional mESC clones show efficient Cre-dependent biotinylated protein secretion *in vitro*, including a positive control, Alpl. In **(dd)** asterisk (*) points to a non-specific band, whereas the arrow is the secreted Alpl band. **(bb-dd)** For each slice, all lanes are from the same western blot (see **Source Data** for uncropped blots). IB = immunoblot. **(y)** is a representative image (one was taken), and **(z-aa)** are representative images from two acquired. The western blots in **(bb-dd)** were performed once, but similar results were obtained with another mESC clone in **Fig. 4j-k**.

ee, Alpl secretion is unaffected upon *BirA*G3-ER* expression in mouse embryonic stem cells (mESCs). Alkaline phosphatase (Alpl) secretion into media was compared by fluorescent western blot analysis[31], varying *BirA*G3-ER* and biotin labeling (50 µM supplementation to media for 12 hours) as indicated. This experiment was performed in serum-free OPTI-MEM medium. Note: Streptavidin (680 channel) and ALPL (800 channel) were probed on the same western blot in separate channels. Slice views are from the same blot, but separated for individual channels. Secreted proteins in media were concentrated by centrifugal filters with a 30 kDa cutoff. Quantification of Alpl western blot normalized to total protein using Li-Cor Empiria Studios software and normalization protocols is shown on the bottom of the figure. rAlpl is recombinant Alpl. This result is a representative of three biologically independent repeats (each repeat was run on the western blot twice).

Source data are provided as a **Source Data** file.

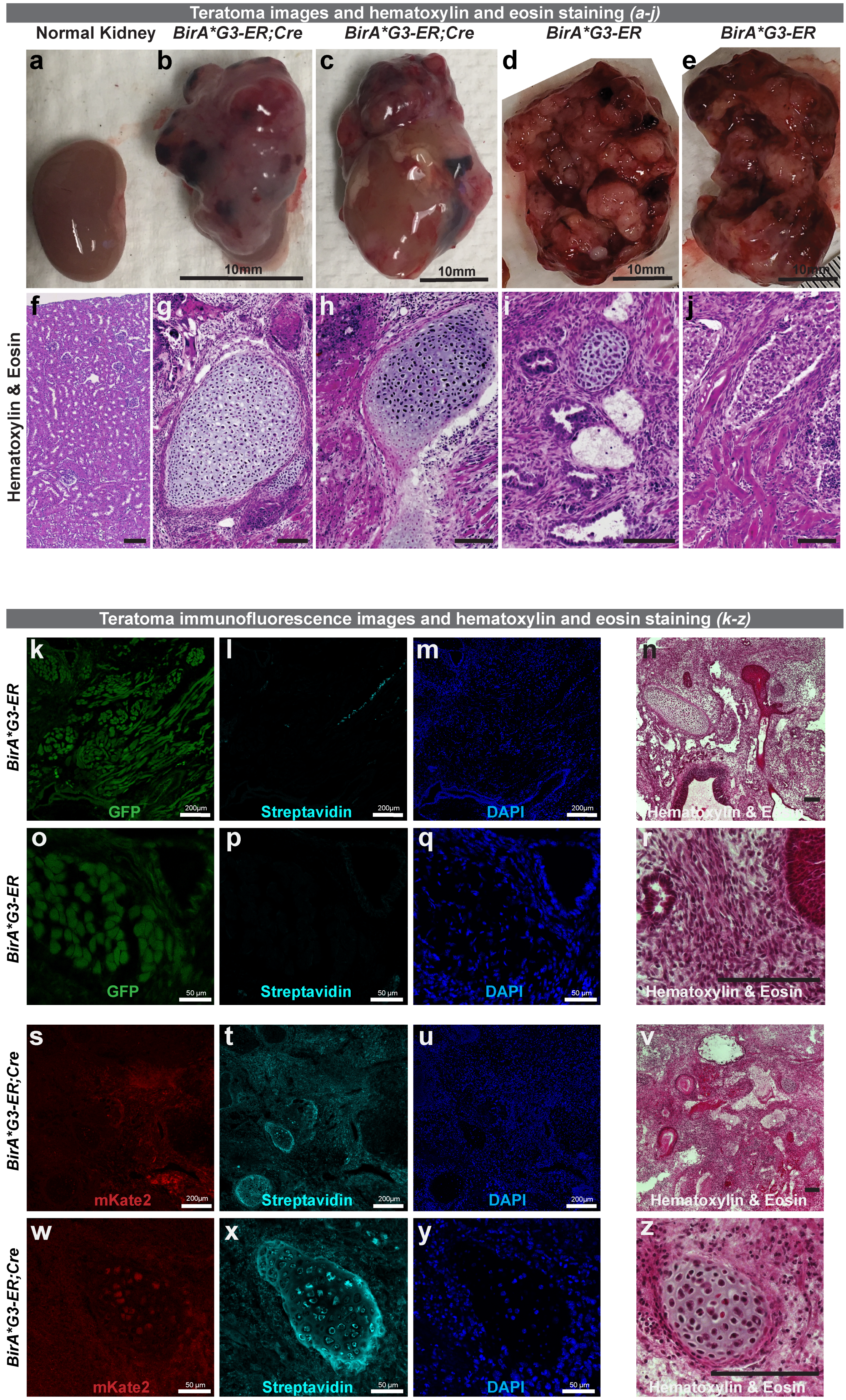

Supplementary Figure 12: Teratoma characterization (Fig. 6 supplement).

Supplementary Figure 12 (Continued).

(a-e) Images of teratomas harvested from mice after 4 weeks of growth under the kidney capsule compared to normal kidney. **(f-j)** Hematoxylin and eosin staining of cryosectioned teratomas generated from *BirA*G3-ER* or *BirA*G3-ER;Cre* mESCs. **(k-z)** Immunofluorescence of cryosectioned teratomas generated from *BirA*G3-ER* or *BirA*G3-ER;Cre* mESCs showing native fluorescence of reporters (GFP in green and mKate2 in red), streptavidin (cyan), and DAPI (blue). Teratoma experiments are representative results from *n*=4-5 per *BirA*G3-ER* or *BirA*G3-ER;Cre* teratoma generated. The immunofluorescence results are representative from two repeats. **(f-j, n, r, v, z)** Scale: 100 µm.

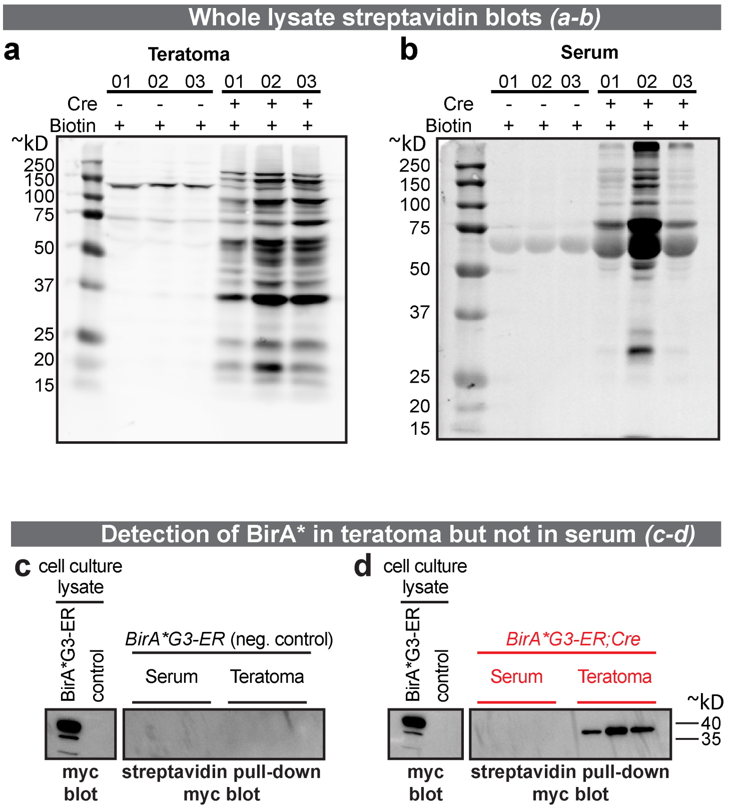

Supplementary Figure 13: Teratoma and serum streptavidin and BirA*G3-ER-myc western blots (Fig. 6 supplement).

a-b, Streptavidin western blot of teratoma protein lysate (25 µg, **a**) and total serum (5 µg, **b**) from mice with *BirA*G3-ER* or *BirA*G3-ER;Cre* teratomas. **(a)** All lanes are from the same western blot (see **Source Data** for uncropped blots). **(b)** All lanes are from the same western blot (see **Source Data** for uncropped blots). **(a-b)** are representative western blots from five repeats each.

c-d, BirA*G3-ER-myc was detected in teratomas but not in serum. The *BirA*G3-ER* (**c**) and *BirA*G3-ER;Cre* (**d**, red text) samples were run on different gels at the same time, and were exposed for the same length of time. *BirA*G3-ER* expressing cells in culture were used as controls. **(c)** All lanes are from the same western blot (see **Source Data** for uncropped blots). **(d)** All lanes are from the same western blot (see **Source Data** for uncropped blots). This western blot was done once, however, BirA*G3 peptides were also searched for in the mass spectrometry data.

Source data are provided as a **Source Data** file.

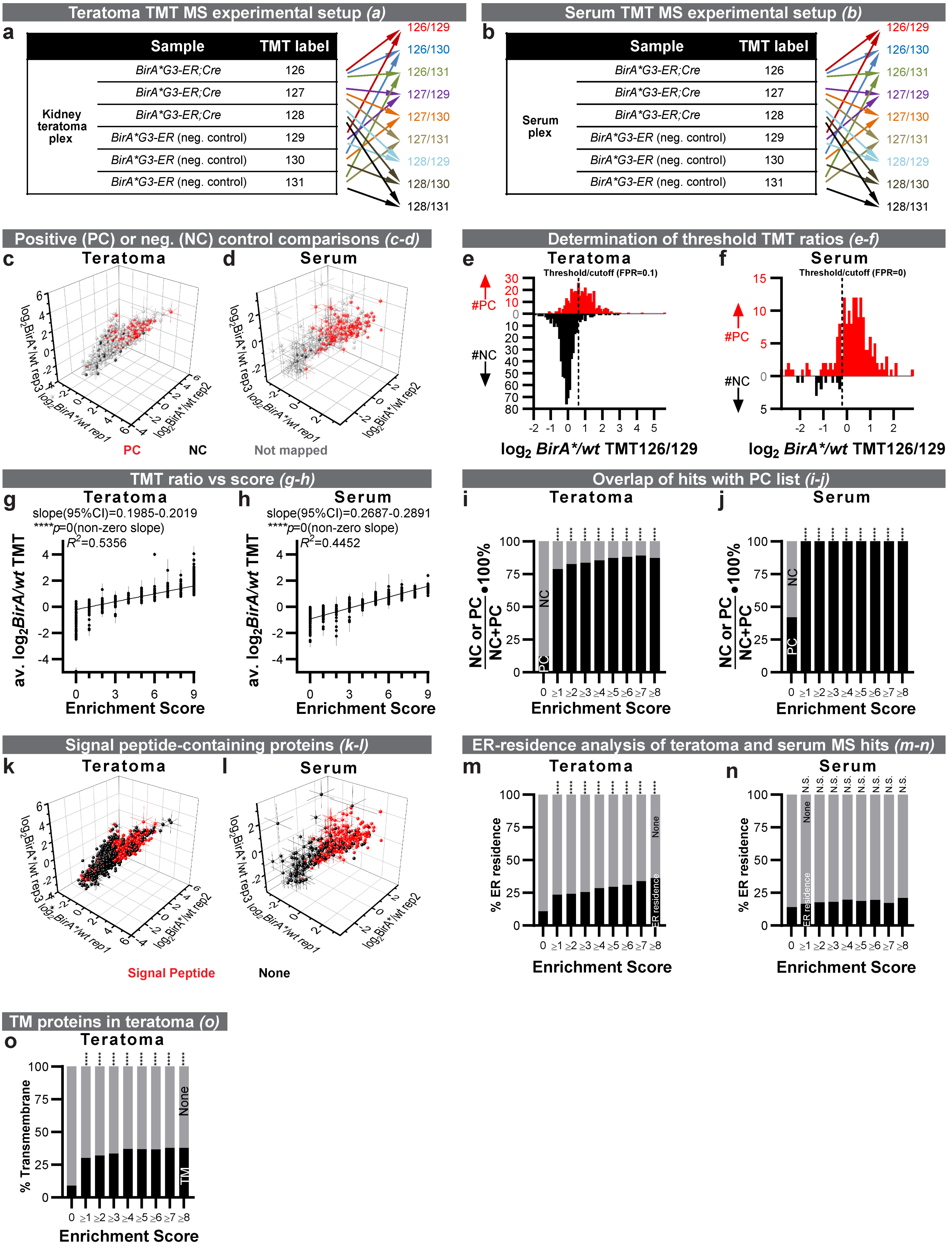

Supplementary Figure 14: Thresholding and signal peptide analyses of biotinylated serum and teratoma proteins identified using tandem mass-tag (TMT) mass spectrometry (MS) using BirA*G3-ER labeling (Fig. 7 supplement).

Supplementary Figure 14 (Continued).

a-b, Experimental setups for the identification of serum and teratoma biotinylated proteins. The different TMT state signals were compared to generate TMT ratios (right of the arrows). There were 9 TMT ratio comparisons for teratomas and 9 for serum.

c-d, Teratoma and serum $\log_2$(*BirA*G3-ER / wt*) TMT-ratios in three replicates, with each point is n=3 comparisons, mean±SEM $\log_2$TMT ratio. Proteins identified with MS were compared to positive control (PC) secreted protein/receptor (red points) and negative control (NC, intracellular; black points).

e-f, Representative #NC or #PC versus *BirA*G3-ER/wt* TMT ratio graph (out of nine). A threshold TMT ratio was chosen for which %NC/%PC ≤ FPR (false positive rate; FPR=0.1 for teratomas and FPR=0 for serum).

g-h, Increased TMT-ratios are associated with higher Enrichment Scores (E-S). Each point is a mean±SEM $\log_2$TMT ratio for each identified protein. Linear regression results of a two-tailed F-test are presented.

i-j, Hits (Enrichment Score ≥ 1) have higher #PC/(#PC+#NC). Statistics: Two-sided Fisher's exact test. Teratoma *p*-values (****) from left to right **(i)**: $5.92 \cdot 10^{-99}$, $3.80 \cdot 10^{-103}$, $1.49 \cdot 10^{-99}$, $3.22 \cdot 10^{-91}$, $8.01 \cdot 10^{-88}$, $3.66 \cdot 10^{-80}$, $1.50 \cdot 10^{-69}$, $8.40 \cdot 10^{-56}$. Serum *p*-values (****) from left to right **(j)**: $4.35 \cdot 10^{-17}$, $4.72 \cdot 10^{-16}$, $1.77 \cdot 10^{-15}$, $1.28 \cdot 10^{-14}$, $8.60 \cdot 10^{-14}$, $5.53 \cdot 10^{-12}$, $4.14 \cdot 10^{-8}$, 0.000002.

k-l, Proteins with an identified signal peptide[25] (red) were mapped onto the $\log_2$(*BirA*G3-ER/wt*) TMT-ratios in three replicates graph. Each point is n=3 comparisons, mean±SEM.

m-n, Teratoma hits are enriched for ER-resident proteins, while serum hits are not enriched for ER-resident proteins. Statistics: Two-sided Fisher's exact test. Teratoma *p*-values (****) from left to right **(m)**: $1.75 \cdot 10^{-30}$, $8.23 \cdot 10^{-30}$, $2.55 \cdot 10^{-31}$, $8.57 \cdot 10^{-36}$, $7.66 \cdot 10^{-34}$, $9.17 \cdot 10^{-34}$, $1.20 \cdot 10^{-34}$, $1.78 \cdot 10^{-33}$. *p*-values from left to right in **(n)** (N.S. means not significant): 0.5692, 0.3852, 0.3749, 0.2306, 0.3422, 0.2416, 0.6609, 0.3153.

o, Teratoma hits are enriched for transmembrane-domain (TM) containing proteins. Two-sided Fisher's exact test. Teratoma *p*-values (****) from left to right: $5.69 \cdot 10^{-77}$, $6.93 \cdot 10^{-78}$, $1.19 \cdot 10^{-77}$, $7.11 \cdot 10^{-82}$, $4.94 \cdot 10^{-70}$, $3.01 \cdot 10^{-61}$, $1.06 \cdot 10^{-53}$, $5.94 \cdot 10^{-45}$.

Source data are provided as a **Source Data** file.

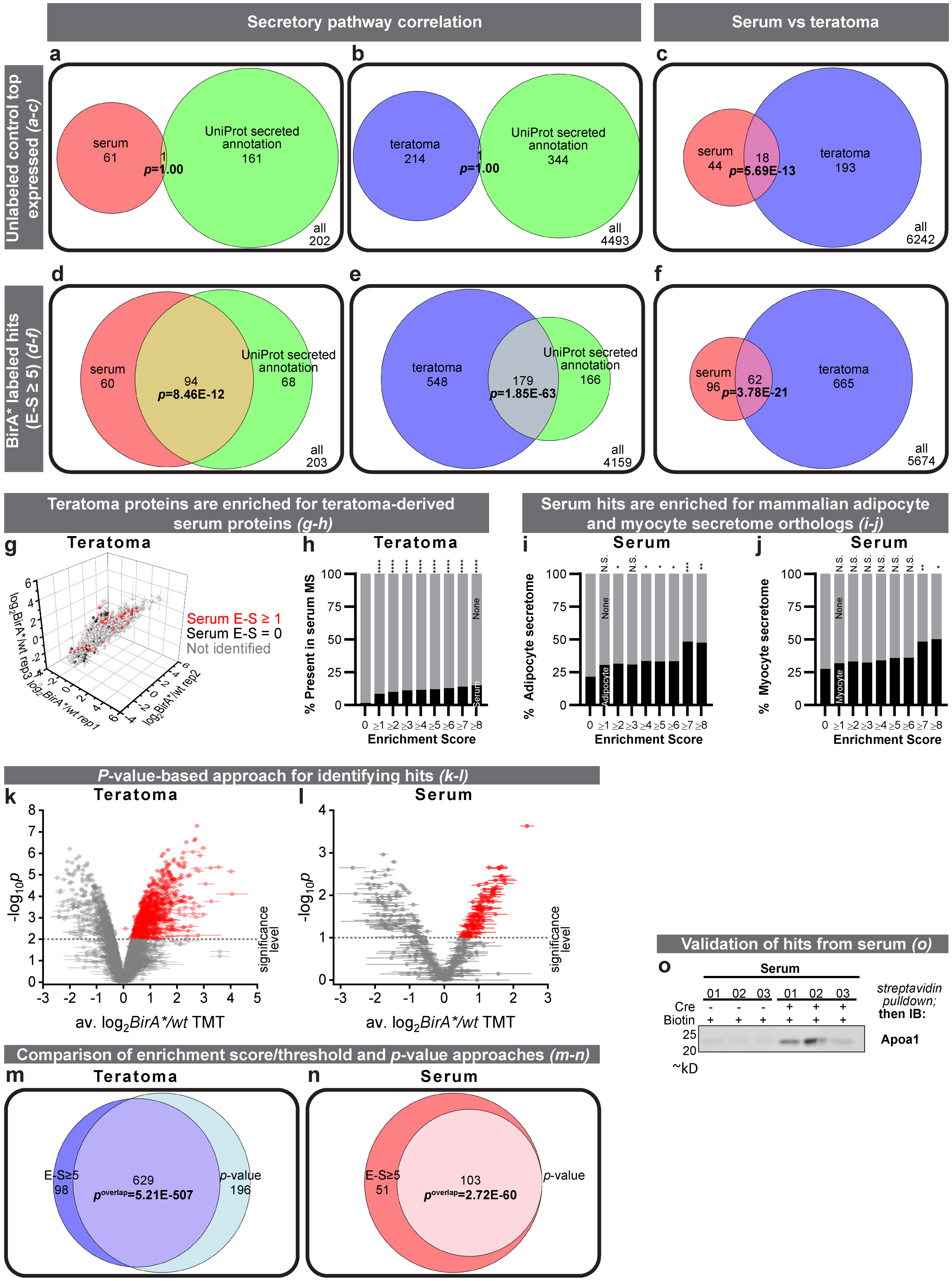

Supplementary Figure 15: Additional and complementary analyses of teratoma and serum datasets (Fig. 7-8 supplement).

Supplementary Figure 15 (Continued).

a-f, Complementary secretory pathway analysis: the top expressed (average reading cutoff = 0.5) control unlabeled background proteins from teratoma and serum show no significant enrichment for UniProt secretion annotation (**a-b**). The overlap between control serum and teratoma does not include annotated secreted proteins, and may instead include most abundant background proteins in both samples that non-specifically bind to streptavidin (**c**). By contrast, *BirA*G3-ER;Cre*-biotinylated serum and teratoma hits (Enrichment Score (E-S) ≥ 5) are enriched for secreted proteins and show highly statistically-significant overlap with each other (p=3.78×10^{-21}; **d-f**). Statistics: hypergeometric test (one-sided p-values).

g-h, As E-S increases, the fraction of teratoma hits that were identified in the serum increases; however, not all teratoma hits were identified in the serum, suggesting that only a fraction of proteins is secreted. Teratoma p-values (****) from left to right: 4.63•10^{-32}, 6.06•10^{-38}, 3.86•10^{-40}, 2.34•10^{-36}, 1.51•10^{-34}, 6.85•10^{-34}, 4.00•10^{-32}, 1.39•10^{-30}.

i-j, Serum hits are enriched for mammalian adipocyte **(i)** and myocyte **(j)** secretome orthologs (see **Methods**). Two-tailed Fisher's exact test. p-values (**i**; left to right): 0.0624, 0.0416, 0.0636, 0.0234, 0.0348, 0.0460, 0.0003, 0.0033. p-values (**j**; left to right): 0.4278, 0.2941, 0.4016, 0.2237, 0.1637, 0.1737, 0.0075, 0.0110.

k-l, A p-value-based alternative approach for identifying hits. Volcano plots of average $\log_2$(*BirA*G3-ER / wt*) TMT-ratios (mean ± standard error of the mean; n=9 comparisons). Hits (red) were defined as having p<0.1 (serum) and p<0.01 (teratoma) and fold-change>1. p-values were calculated by comparing *BirA*G3-ER/*mean of all samples and *wt/*mean of all samples TMT signal ratios (n=3 each) using two-sample t-tests.

m-n, The p-value-based approach for identifying hits (**k-l**) showed strong correlation with the score/thresholding-based method.

o, Western blot analysis of target hits in streptavidin pulldown of serum samples. This is an analysis of extended set of samples from **Fig. 8c** for which sample 01 from *BirA*G3-ER;Cre* was available. This is a representative result from two western blots (the other one is in **Fig. 8c**). All lanes are from the same blot (see **Source Data** for uncropped blots).

Source data are provided as a **Source Data** file.

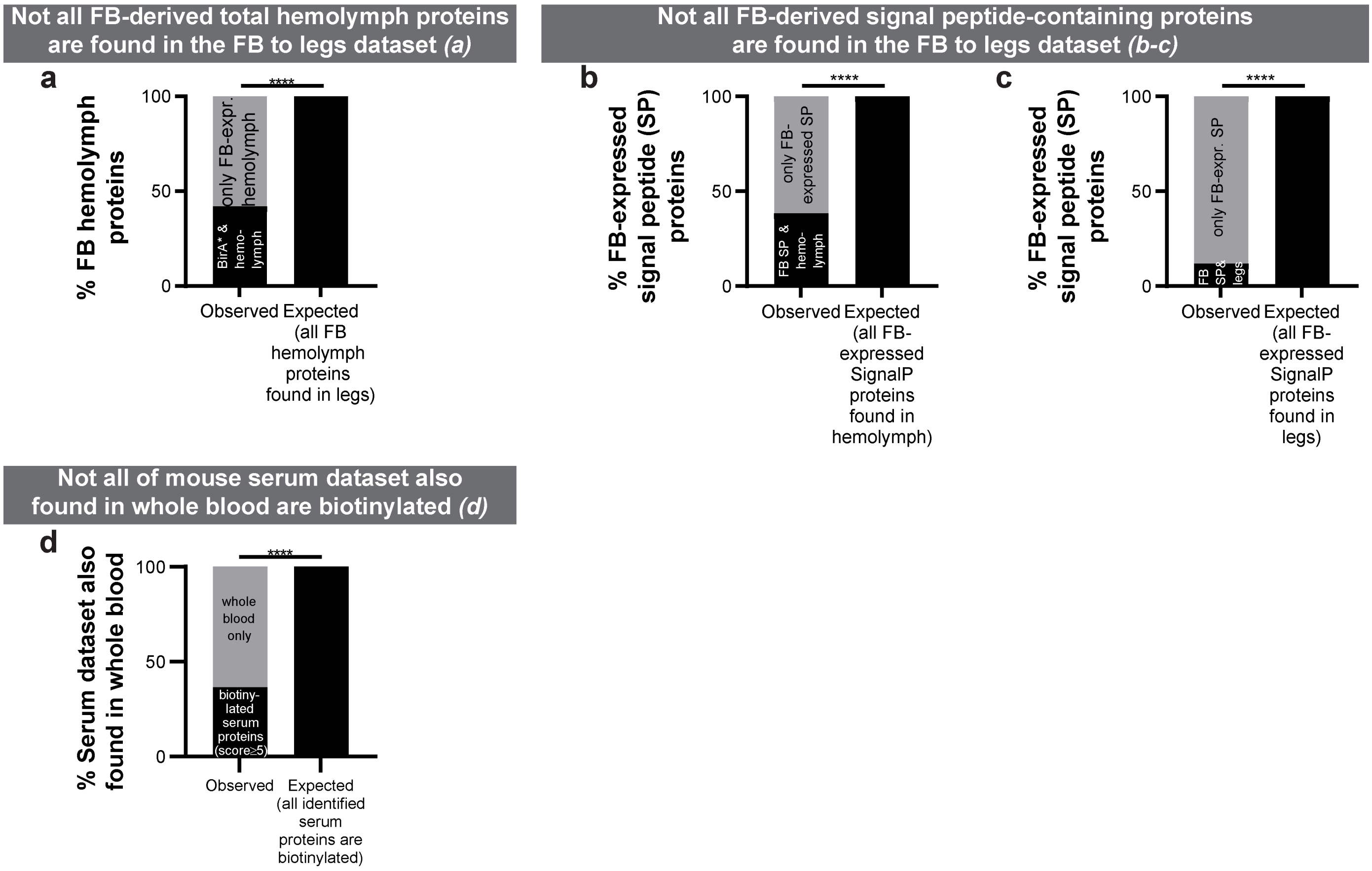

Supplementary Figure 16: Not all fat body (FB)-derived total hemolymph proteins or signal peptide-containing proteins are identified in the FB-to-legs proteomics dataset, and only some serum proteins are biotinylated (Discussion supplement).

a-d, Statistics: two tailed chi-squared test.

a, Not all FB-derived total hemolymph proteins are identified in the FB-to-legs dataset. We compared highest-confidence (observed more than 12 times and present in the positive control [PC] list) FB-expressed (based on FlyAtlas microarray[29], "present cutoff"≥4) hemolymph-resident proteins (n=101), with enrichment score (E-S)≥1 FB-expressed PC FB-to-legs dataset (N=101). ****$p=6.90 \cdot 10^{-20}$.

b-c, Not all FB-derived signal peptide-containing proteins are identified in the FB-to-legs dataset. **(b)** Hemolymph-resident proteins (observed at least once) that are FB-expressed (based on FlyAtlas microarray[29], "present cutoff"≥4) and have a signal peptide (SP or SignalP)[32] (n=334) were compared to all FB-expressed SignalP-containing proteins (N=869). The presence of a signal peptide does not indicate that a protein will be secreted locally or systemically. The presence of a signal peptide containing FB protein within the hemolymph suggests that this protein was secreted systemically. ****$p=4.15 \cdot 10^{-170}$. **(c)** E-S≥1 FB-expressed PC FB-to-legs SignalP-containing proteins (n=103) were compared to all FB-expressed SignalP-containing proteins (N=869). ****$p=8.23 \cdot 10^{-300}$.

d, We compared mouse and human blood proteome data (cells removed; see **Methods**) that were identified in our serum MS dataset (N=187) to E-S ≥ 5 serum hits (n=68). Only some serum proteins were biotinylated, suggesting of specificity to teratoma-labeling. ****$p=7.57 \cdot 10^{-40}$.

Source data are provided as a **Source Data** file.

Supplementary Table 1: Drosophila stocks and genotypes used in this study

Genotype	Source	Notes
Oregon^R (referred to as *Ore^R*)	N.P. lab	*wt* flies used in **Supplementary Figure 4g-i and 12**
w^{1118}	N.P. lab	*wt* control that was used where indicated
*w;10xUAS-IgK-HA-BirA*R118G-KDEL,w$^+$(attP40,y$^+$)*	This study	
*w;;10xUAS-IgK-HA-BirA*R118G-KDEL,w$^+$(attP2,y$^+$)*	This study	
*w;10xUAS-HA-BirA*R118G,w$^+$(attP40,y$^+$)*	This study	
*w;;10xUAS-HA-BirA*R118G,w$^+$(attP2,y$^+$)*	This study	
*w;10xUAS-BiP-myc-BirA*G3-KDEL,w$^+$(attP40,y$^+$)*	This study	
*w;10xUAS-myc-BirA*G3,w$^+$(attP40,y$^+$)*	This study	
w;10xUAS-IVS-mCD8::GFP (attp40)	Bloomington #32186 [33]	Overexpression control in **Supplementary Figure 1.**
TUB-Gal4	N.P. lab, ref. [34]	**Supplementary Figure 1**
*UAS-BirA*G3-ER-myc/(If or CyO);Dilp215H-Gal4,dilp2^1,Dilp2-HA-Flag(attP2)*	Generated from *w[1118];UAS-Dcr-2;Dilp215H,dilp2^1,Dilp2-HA-Flag(attP2)* (gift of Seung Kim [35])	Used in **Fig. 1b-c** and **Supplementary Figure 2a-h**. These *Dilp2-HA-Flag* flies were previously used to measure changes in concentrations of systemic Dilp2 [35].
LPP-Gal4 (lipophorin-Gal4)	Gift of Akhila Rajan. Generated in Ref. [36,37]	Fat-body (FB)-specific driver (ref. [36,37] and indicated by our preliminary experiments). Used where indicated.
MHC-Gal4 (on chromosome III)	N.P. lab, ref. [38]	Driver used for muscle expression [39]
Dmef2-Gal4	N.P. lab, ref. [40]	Driver used for muscle expression [39,40]
w;tub-gal80^{ts};LPP-Gal4	Gift from Akhila Rajan	**Supplementary Figure 9w.**
w[1118];UAS-mito-HA-GFP	Bloomington #8443	**Fig. 3q-t, Supplementary Figure 10d-f and n-q.**
UAS-CG2145-3xHA(attP86Fb)	FlyORF F003749 [41]	**Fig. 3i-p and u-x, Supplementary Figure 10a-c, g-i, j-m, and r-gg**
Control (Luc-i) (Luceferase RNAi; JF01355)	Harvard Transgenic RNAi Project (TRiP) [42]	
Control (w-i) (white RNAi; HMS00017)	TRiP	
Control (attP) (attP[VIE-260B]; 60100)	Vienna *Drosophila* Resource Center (VDRC) [43]	
Control (v-i) (vermillion RNAi; 2155R-1)	Japan National Institute of Genetics (NIG)	
Control (GFP-i) (GFP RNAi; HMS00773)	TRiP	
CG31326-i-1 (51890GD)	VDRC	
CG31326-i-2 (31326R-2)	NIG	
CG31326-i-3 (31326R-1)	NIG	
CG2145-i-1 (14874GD)	VDRC	
CG2145-i-2 (HMJ23623)	TRiP	
CG4332-i-1 (4332R-3)	NIG	
CG4332-i-2 (HMS01341)	TRiP	

Supplementary Table 2: Oligonucleotides used in this study

Oligo Name	Sequence
pDISPLAY R118G 2 sense	GCCGTGGTGGCCGGGGTCGGAAATGG
pDISPLAY R118G 2 antisense	CCATTTCCGACCCCGGCCACCACGGC
BirA-IgK-gtwy-F	CACCATGGAGACAGACACACTC
BirA-KDEL-gtwy-R	TCACAGCTCGTCCTTTGAACC
pDspl-HA-BirA-gtwy-F	CACCATGGCATATCCATATGATGTTCCAGATTATGCTGG
pDspl-BirA-gtwy-R	TCATTTTTCTGCACTACGCAGGGATATTTC
BIP_myc_dtopo_1F	CACCATGAAGTTATGCATATTACTGGCCGTCGTGGCCTTTGTT GGCCTCTCGCTCGGGGAACAAAAGCTTATTTCTGAAGAGG
KDEL_BrA*2_dtopo_1R	CTATTACAGCTCGTCCTTCTTTTCGGCAGACCGCA
Myc_dtopo_1F	CACCATGGAACAAAAGCTTATTT
BirA*2_dtopo_1R	CTATTACTTTTCGGCAGACCGCA
uCRE_F	GCATTTCTGGGGATTGCTTA
uCRE_R	TTGCCCCTGTTTCACTATCC

Supplementary Table 3: Positive control secreted factors involved in intercellular signaling identified in total hemolymph MS

Factor(s)	Fly hemolymph experiments	Human ortholog (DIOPT[44] and other references)	References
IDGFs (imaginal disc growth factor)	most	CHIT1	[29,45-50]
ADGF-A (adenosine deaminase growth factor A)	N-glycan	CECR1	[51,52]
Tsp (thrombospondin)	N-glycan	THBS	[53,54]
Ccn	N-glycan	CTGF	[55,56]
spz (spätzle)	N-glycan		[57,58]
Sog (short gastrulation)	N-glycan	CHRD	[59,60]
Nec (necrotic)	most	SERPINC1	[61]
Spn5 (serpin 88Ea)	most	SERPINI1	[62,63]
Ance (Angiotensin converting enzyme)	most	ACE	[64]
Pvf3 (PDGF and VEGF related factor 3)	N-glycan	PDGF/VEGF	[65]
Upd1 (unpaired 1)	Neat, targeted	IL6	[66,67]
Egr (Eiger)	N-glycan	TNF-α	[68]
Tk (Tachykinin)	3-10 kD	TAC1	[69]
CCHa1 (CCH amide 1)	<3 kD, non-tryptic search		[70]
SDR (secreted decoy of insulin receptor)	N-glycan	INSR	[71]
GBP2 (growth blocking peptide 2)	most	EGFs[72]	[72]
PGRPs (Peptidoglycan recognition proteins; multiple identified)	most	PGLYRPs	[73]
Mav (maverick)	neat (older flies)	TGF-β family	[74]

Note: Upd1 was identified from a targeted MS analysis of unfractionated fly blood using a known tryptic peptide from ref. [67].

Supplementary Table 4: Identified positive control fat body to legs and muscle trafficking proteins

Factor	Function	Ref.	Human ortholog	DIOPT score[44](v.)	Dataset
Lsp1β (larval serum protein 1β)	Lsp complex consisting of α, β, γ subunits: FB-enriched expression; storage; cuticle contribution in several insect species; in *Calliphora vicina* contributes to muscle in thorax; protein abundance associated with adult wing size; Lsp1γ is important for and traffics to muscle adhesions.	75-80			BirA*G3-ER, BirA*R118G-ER
Lpp (apolipophorin)	Produced in FB; transport hydrophobic molecules including lipids, sterols, membrane lipids, and signaling secreted factors (Hh and Wg) to and in tissues including imaginal discs; interacts with lipophorin receptors.	37,81-85	APOB (apolipoprotein B)	v.5: 4<br>v.6: 5	BirA*G3-ER, BirA*R118G-ER
LTP (apolipoprotein lipid transfer particle)	Produced in FB and traffics to distal tissues including imaginal discs in which lipids get transferred from Lpp to imaginal disc cells through LTP; interacts with lipophorin receptors.	86,87	APOB (apolipoprotein B)	v.5: 3<br>v.6: 4	BirA*G3-ER
Idgf1 (Imaginal disc growth factor 1)	Secreted from FB and traffics to imaginal discs to promote cell proliferation; may act synergistically with insulin; may be regulated by miR-8 in FB and miR-8 may regulate whole-body growth; cuticle formation	29,45,46,48,49	CHIT1 (chitinase 1)	v.5: 1<br>v.6: 2	BirA*G3-ER
Idgf2 (Imaginal disc growth factor 2)	Secreted from FB and traffics to imaginal discs to promote cell proliferation; may act synergistically with insulin; may be regulated by miR-8 in FB and miR-8 may regulate whole-body growth.	45,46,49,50	CHIT1 (chitinase 1)	v.5: 1<br>v.6: 2	BirA*G3-ER
Idgf6 (Imaginal disc growth factor 6)	Expressed in tissues including FB; may be regulated by miR-8 in FB and miR-8 may regulate whole-body growth; cuticle formation; interaction with chitin.	29,45-48	CHIT1 (chitinase 1)	v.5: 1<br>v.6: 2	BirA*G3-ER, BirA*R118G-ER
Adgf-D (adenosine deaminase-related growth factor D)	Secreted from FB and exerts proliferative effects on imaginal disc cells. Adgf-D mutant adults may be under-active. Adgf-D acts outside the cell and degrades extracellular adenosine. Adenosine outside the cell has anti-proliferative, catabolic and energy store wasting effects, acting via adenosine transmembrane transport or adenosine receptor (AdoR), expressed in many tissues. AdoR or extracellular adenosine overexpression cause thoracic and wing defects, and AdoR overexpression causes defects in movement.	51,52,88-92	ADA2/CECR1 (adenosine deaminase 2 or cat eye syndrome chromosome region candidate 1)	v.5: 7<br>v.6: 9	BirA*G3-ER
cv-d (crossveinless d)	Secreted primarily from FB into hemolymph and traffics to imaginal discs. Binds to TGFs Gbb and Dpp and regulates their extracellular transport in imaginal discs. Gbb, Dpp, and cv-d also regulate adult muscle morphology.	37,93,94			BirA*G3-ER, BirA*R118G-ER
Tig (Tiggrin)	ECM protein secreted by FB (also blood cells, and to a lower extent possibly in muscles); binds to muscle adhesion foci and on surface of adult muscle (specific staining pattern at Z-bands) through PS2 integrins and possibly other receptors; regulates muscle adhesion, thickness, and contraction velocity.	80,95-97	TRIP11	v.5: 1	BirA*G3-ER
fon (fondue)	Produced by FB, secreted into hemolymph, and binds to muscle adhesion foci; regulates muscle adhesions and movement speed.	80			BirA*G3-ER

Abbreviations: FB, fat body; Hh, hedgehog; Wg, wingless; TGF, transforming growth factor; Dpp, decapentaplegic; Gbb, glass bottom boat; ECM, extracellular matrix; Ref., references; v., version (DIOPT).

Supplementary Table 5: Identified additional known FB-produced proteins in *LPP-Gal4>BirA** legs

Factor	Function	References	Human ortholog	DIOPT score[44] (version)	Dataset
Acer (Angiotensin-converting enzyme-related)	Peptidase produced by the FB and secreted to hemolymph; regulates sleep and heart functions. Mammalian ACE and ACE2 are expressed in adipose tissue and are involved in obesity.	[98-103]	ACE (angiotensin-converting enzyme), ACE2 (angiotensin-converting enzyme 2)	v.5: 6 (ACE)<br>v.6: 10 (ACE)<br>v.6: 6 (ACE2)	BirA*G3-ER
psh (Persephone)	Protease expressed in FB, upstream of SPE; within Toll pathway.	[29,104]	TMPRSS12	v.5: 1	BirA*G3-ER
GNBP3 (Gram-negative bacteria binding protein 3)	Glucan recognition protein expressed in FB, upstream of SPE, within Toll pathway.	[29,104,105]			BirA*G3-ER
GNBP-like3	Immune glucan-binding protein; may be produced by the FB.	[29,106]			BirA*G3-ER
SPE (Spatzle Processing Enzyme)	Protease expressed in FB, cleaves pro-Spatzle to cause its activation.	[29,107,108]			BirA*G3-ER
TotA (Turandot A)	FB-produced peptide; expression changes due to stress.	[29,109-112]			BirA*G3-ER
MP1 (Melanization Protease 1)	Protease expressed in FB; involved in melanization.	[29,113]			BirA*G3-ER
Sp7 (Serine Protease 7)	Protease expressed in hemocytes and possibly in FB; involved in melanization.	[29,113,114]	F12, HGFAC	v.5: 1	BirA*G3-ER
prc (pericardin)	secreted by FB and binds to cardiac muscle ECM via lonely heart (loh) extracellular receptor	[115]	COL1A1	v. 5.3: 1<br>v. 6: 2	BirA*R118G-ER

Abbreviations: FB, fat body; ECM, extracellular matrix; v., version (DIOPT).

Supplementary Table 6: Additional secreted proteins identified in *LPP-Gal4>BirA*G3-ER* legs

Factor	Function	References	Human ortholog	DIOPT score[44] (version)
Fer1HCH (Ferritin 1 heavy chain homologue)	Ferritin iron carrier, may be secreted systemically.	[116,117]	FTH1, FTHL17, FTMT	v.5: 1<br>v.6: 2
Hmu (hemomucin)	A mucin with a putative signal peptide.	[25,118,119]	APMAP	v.5: 8<br>v.6: 8
Ance-4, Ance-5 (Angiotensin-converting enzyme-4 and 5)	Expressed in FB, has a signal peptide related to Acer.	[25,29,120]	ACE	v.6: 3
teq (tequila)	Protease expressed in FB and head; has a signal peptide; regulates lifespan, memory, and insulin signaling; mammalian PRSS12/neurotrypsin regulates social behavior and cognitive ability, and may be secreted.	[25,29,121-126]	PRSS12 (neurotrypsin)	v.5: 3<br>v.6: 2
Nplp2 (Neuropeptide-like precursor 2)	Neuropeptide.	[127-129]		
Tep2 (thioester containing protein 2)	Immune protein with a signal peptide; expressed in FB; *Anopheles gambiae* Tep1 is in the hemolymph. Mammalian CD109 may be secreted into the blood and may regulate TGF-β signaling.	[25,29,130-136]	CD109	v.5: 7<br>v.6: 7
tok (tolkin) or tlr (tolloid-related)	Secreted protease that processes pro-TGF secreted factors (e.g., dawdle) and inhibitors (e.g., short gastrulation); has functions in axon formation of motor neurons, and wing disc formation; expressed in FB. In *Drosophila* and mammals, TGF-β pathway regulates metabolism, including muscles. In mammals, BMP1 and TLL1 proteases cleave and activate myostatin and GDF-11.	[29,59,137-142]	BMP1 (bone morphogenetic protein 1), TLL1 (tolloid like 1)	v.5: 7<br>v.6: 9
modSP (modular serine protease)	Secreted protease in the Toll pathway.	[143]	several proteases including TMPRSS6, TMPRSS15, PAMR1, MASP2, MASP1, C1S, CSMD2	v.5: 1<br>v.6: 1
cathD (cathepsin D)	Protease with a signal peptide; loss of function causes neuronal defects.<br>Mammalian secreted pro-CTSD has growth-factor activity, and CTSD proteolytically regulates growth factors and cytokines (e.g., insulin, glucagon, osteocalcin, IGFBP, FGF, interleukin-1, plasminogen); CTSD mutations result in neurodegeneration and psychomotor defects.	[25,144-151]	CTSD (cathepsin D)	v.5: 9<br>v.6: 10
Ndg (nidogen)	ECM protein. Mammalian protein is part of basement membrane.	[152,153]	NID1 (nidogen 1)	v.5: 8<br>v.6: 10

Abbreviations: TGF, transforming growth factor; GDF, growth differentiation factor; IGFBP, insulin-like growth factor binding protein; FGF, fibroblast growth factor; ECM, extracellular matrix; v., version (DIOPT).

Supplementary Table 7: *Drosophila* orthologs of human secreted proteins involved in systemic pathways identified in BirA*G3-ER fat body to legs mass spectrometry.

Mammalian factor(s)	Identified *Drosophila* ortholog with fly to human DIOPT score (v.6 or, if indicated, v.5)[44]	Mammalian function	References
ACE (angiotensin-converting enzyme)	Acer (score=10), Ance-4 (score=3), Ance-5 (score=5)	Involved in obesity and expressed in adipose tissue.	98-103
APOB (apolipoprotein B)	Lpp (score=5), LTP (score=4)	Lipid transport between tissues; can bind to sonic hedgehog (SHH). Highly expressed in the liver.	37,81-87,154,155
TLL1 (tolloid like 1), BMP1 (bone morphogenetic protein 1)	tok (score=9)	These factors cleave and activate myostatin and GDF-11, which are systemic TGF-β factors regulating metabolism, including muscles.	59,137-142,156,157
LNPEP (leucyl and cystinyl aminopeptidase)	CG4467 (score=4)	Circulates in blood during pregnancy. Cuts hormone peptides including oxytocin, met-enkephalin, vasopressin. May be involved in adipose tissue biology, thermogenesis, and glucose translocation.	158-164
TRHDE (thyrotropin-releasing hormone degrading enzyme), ENPEP (glutamyl aminopeptidase)	CG11951 (score=5 for LVRN, ANPEP; score=4 for TRHDE, ENPEP)	TRHDE: cleaves thyrotropin-releasing hormone. ENPEP: secreted during pregnancy; cuts cholecystokinin 8 and angiotensin 2.	165-168
ECE1 (Endothelin converting enzyme 1)	CG14526 (score=3), CG14528 (score=2), CG9507 (score=2)	Converts big endothelin to active endothelin via proteolysis. Endothelin in turn regulates organismal developmental progression, growth, disease progression, vascular constriction, and pressure of blood. ECE1 may be regulated by blood flow, metabolic dysfunction. ECE1 can be secreted through cleavage of the transmembrane form.	169-172
MME (membrane metalloendopeptidase) (also known as NEP/neprilysin)	CG14528 (score=2)	MME/NEP can be secreted through cleavage of the transmembrane form and detected in blood. Cuts hormone peptides including glucagon, somatostatin, insulin, substance P, cholecystokinin, enkephalins, angiotensins, bradykinin, natriuretic peptides, and others, thus playing roles in development, homeostasis, and disease. Expressed in the fat tissue along with other tissues.	155,173,174
KLKB1 (kallikrein B1) (also known as plasma kallikrein)	CG8586 (score=2)	KLKB1 or blood plasma-secreted kallikrein is part of the hormonal kallikrein-kinin (e.g., bradykinin) network. Kinins play roles in metabolism, diabetes, blood pressure control, inflammation. KLKB1 levels are related to metabolic disorders and diabetes. KLKB1 can also cleave plasminogen, and this regulates adipogenesis. Expression enriched in the liver.	155,175-177
CD109	Tep2 (score=7), Tep4 (score=5)	May be secreted into the blood and possibly regulate TGF-β signaling.	134-136
CTSD (Cathepsin D)	CathD (score=10)	CTSD regulates through cleavage hormones, cytokines and growth factors (such as IGFBP, FGF, glucagon, insulin, osteocalcin, interleukin-1, and plasminogen). Secreted pro-CTSD may be a growth factor. Also, phsychomotor and neurodegeneration defects may results from CTSD mutations.	146-151

Abbreviations: TGF, transforming growth factor; GDF, growth differentiation factor; IGFBP, insulin-like growth factor binding protein; FGF, fibroblast growth factor; v., version (DIOPT).

Supplementary Table 8: Identified fat body secreted factors affecting muscle function

Factor	Putative human orthologs (with references)	DIOPT score (v.)[44]	SP [25]	*LPP-Gal4>RNAi* muscle phenotype (2 lines)	*LPP-Gal4>RNAi* FB phenotype	*Dmef2-Gal4>RNAi* muscle phenotype and microarray expression[29]	Dataset	E-S
CG2145	ENDOU (poly-U-specific placental endonuclease) – signal-peptide-containing RNA-binding protein of uncharacterized function in muscles or adipose tissue; has a role in B lymphocytes[178-180].	v.5: 7<br>v.6: 9	Y	climbing ability↓<br>protein aggregates↑	No significant effects on FB lipid-droplets	No consistent, statistically-significant climbing-ability defects.<br><br>*CG2145-mRNA* is enriched in adult-FB over muscles (larval-carcass)[29]	BirA*G3-ER<br><br>FB-expressed CG2145-3xHA binds specifically to muscle organ (near muscles/neurons within muscle organ)	4
CG4332	CLPTM1L (cisplatin resistance related protein or cleft-lip and palate-associated transmembrane protein 1-like) – no known functions in adipose or muscle tissues. Variations in DNA sequence and expression levels are associated with tumor division and apoptosis[181-184].	v.6: 10	Y	climbing ability↓<br>protein aggregates↑	No significant effects on FB lipid-droplets	No consistent, statistically-significant climbing-ability defects	BirA*R118G-ER	2
CG31326	1. PAMR1 (peptidase domain-containing associated with muscle regeneration-1) – expression associated with muscle regeneration [185] (but no functional data in muscles or adipose tissue), and overexpression in breast cancer cells may reduce growth *in vitro*[186]<br>2. Coagulation factor FVII – may be secreted by adipose tissue and liver and is associated with obesity, insulin resistance, type-2 diabetes, and high fat diet feeding[187,188].	1. v.5: 1 (PAMR1)<br>2. v.6: 1 (FVII)	Y	climbing ability↓<br>protein aggregates↑		No consistent, statistically-significant climbing-ability defects.<br><br>*CG31326-mRNA* is enriched in adult-FB over muscles (larval-carcass)[29]	BirA*G3-ER	4

Abbreviations: SP, signal peptide; FB, fat body; MS, mass spectrometry; v., version (DIOPT); E-S, Enrichment Score.

Supplementary Table 9: BirA*G3-ER teratoma streptavidin enriched (Enrichment score≥1) and non-enriched (Enrichment score=0) proteome lineage markers

Gene symbol	GeneID	Enrichment score	Lineage marker for:
Sgcd	24052	9	Mesoderm (Heart)
Sst	20604	9	Endoderm (Pancreas)
Scg2	20254	8	Ectoderm (CNS/PNS)
Gria2	14800	6	Ectoderm (CNS/PNS)
Cp	12870	6	Endoderm (Liver)
Cfb	14962	6	Endoderm (Liver)
Gria1	14799	5	Ectoderm (CNS/PNS)
Erp27	69187	5	Endoderm (Pancreas)
Gpd1	14555	3	Mesoderm (Adipose tissue)
Lsp1	16985	3	Mesoderm (Spleen and blood)
Tenm2	23964	3	Mesoderm (Heart)
Cps1	227231	2	Endoderm (Liver)
Atp2b2	11941	2	Ectoderm (CNS/PNS)
Slc1a2	20511	2	Ectoderm (CNS/PNS)
Gap43	14432	2	Ectoderm (CNS/PNS)
Snap91	20616	1	Ectoderm (CNS/PNS)
Rtn1	104001	1	Ectoderm (CNS/PNS)
Nefl	18039	0	Ectoderm (CNS/PNS)
Gfap	14580	0	Ectoderm (CNS/PNS)
Myo1a	432516	0	Endoderm (Gut)
Krt15	16665	0	Ectoderm (Skin)
Krt17	16667	0	Ectoderm (Skin)
Myl4	17896	0	Mesoderm (heart)
Actl6b	83766	0	Ectoderm (CNS/PNS)
Hmgcs2	15360	0	Endoderm (Liver)
Rtn1	104001	0	Ectoderm (CNS/PNS)
Trim55	381485	0	Mesoderm (Heart)
Smtnl2	276829	0	Mesoderm (Muscle)
Lin28a	83557	0	Primitive or non-differentiated

Abbreviations: CNS (central nervous system), PNS (peripheral nervous system)
Marker reference: [189]

Supplementary References

1 Eichelbaum, K., Winter, M., Diaz, M. B., Herzig, S. & Krijgsveld, J. Selective enrichment of newly synthesized proteins for quantitative secretome analysis. *Nat Biotechnol* **30**, 984-990 (2012).

2 Rechavi, O. *et al.* Trans-SILAC: sorting out the non-cell-autonomous proteome. *Nat Methods* **7**, 923-927 (2010).

3 Gauthier, N. P. *et al.* Cell-selective labeling using amino acid precursors for proteomic studies of multicellular environments. *Nat Methods* **10**, 768-773 (2013).

4 Li, Z. *et al.* Nitrilase-Activatable Noncanonical Amino Acid Precursors for Cell-Selective Metabolic Labeling of Proteomes. *ACS Chem Biol* **11**, 3273-3277 (2016).

5 Yuet, K. P. *et al.* Cell-specific proteomic analysis in Caenorhabditis elegans. *Proc Natl Acad Sci* **112**, 2705-2710 (2015).

6 Erdmann, I. *et al.* Cell-selective labelling of proteomes in Drosophila melanogaster. *Nat Commun* **6** (2015).

7 Elliott, T. S. *et al.* Proteome labeling and protein identification in specific tissues and at specific developmental stages in an animal. *Nat Biotechnol* **32**, 465-472 (2014).

8 Barrett, R. M., Liu, H.-w., Jin, H., Goodman, R. H. & Cohen, M. S. Cell-specific Profiling of Nascent Proteomes Using Orthogonal Enzyme-mediated Puromycin Incorporation. *ACS Chem Biol* **11**, 1532-1536 (2016).

9 Géminard, C., Rulifson, E. J. & Léopold, P. Remote control of insulin secretion by fat cells in Drosophila. *Cell Metab* **10**, 199-207 (2009).

10 Sandoval, P. C. *et al.* Proteome-wide measurement of protein half-lives and translation rates in vasopressin-sensitive collecting duct cells. *J Am Soc Nephrol* **24**, 1793-1805 (2013).

11 Harashima, S.-i., Clark, A., Christie, M. R. & Notkins, A. L. The dense core transmembrane vesicle protein IA-2 is a regulator of vesicle number and insulin secretion. *Proc Natl Acad Sci* **102**, 8704-8709 (2005).

12 Toyama, B. H. *et al.* Identification of long-lived proteins reveals exceptional stability of essential cellular structures. *Cell* **154**, 971-982 (2013).

13 Tape, C. J. *et al.* Cell-specific labeling enzymes for analysis of cell–cell communication in continuous co-culture. *Mol Cell Proteomics* **13**, 1866-1876 (2014).

14 Elliott, T. S., Bianco, A., Townsley, F. M., Fried, S. D. & Chin, J. W. Tagging and Enriching Proteins Enables Cell-Specific Proteomics. *Cell Chem Biol* **23**, 805-815 (2016).

15 Snyder, A. L. & Brustad, E. M. Tissue-Specific Proteome Tagging: An Orthogonal Approach. *Chembiochem* **15**, 1731-1733 (2014).

16 Liu, J., Xu, Y., Stoleru, D. & Salic, A. Imaging protein synthesis in cells and tissues with an alkyne analog of puromycin. *Proc Natl Acad Sci* **109**, 413-418 (2012).

17 Rhee, H.-W. *et al.* Proteomic mapping of mitochondria in living cells via spatially restricted enzymatic tagging. *Science* **339**, 1328-1331 (2013).

18 Chen, C.-L. *et al.* Proteomic mapping in live Drosophila tissues using an engineered ascorbate peroxidase. *Proc Natl Acad Sci* **112**, 12093-12098 (2015).

19 Roux, K. J., Kim, D. I., Raida, M. & Burke, B. A promiscuous biotin ligase fusion protein identifies proximal and interacting proteins in mammalian cells. *J Cell Biol* **196**, 801-810 (2012).

20 Liu, X. *et al.* In Situ Capture of Chromatin Interactions by Biotinylated dCas9. *Cell* **170**, 1028-1043. e1019 (2017).

21 Chakravarti, B., Fathy, P., Sindicich, M., Mallik, B. & Chakravarti, D. N. Comparison of SYPRO Ruby and Flamingo fluorescent stains for application in proteomic research. *Anal Biochem* **398**, 1-6 (2010).

22 Riedel, F., Gillingham, A. K., Rosa-Ferreira, C., Galindo, A. & Munro, S. An antibody toolkit for the study of membrane traffic in Drosophila melanogaster. *Bio Open*, bio. 018937 (2016).

23 Soler, C., Daczewska, M., Da Ponte, J. P., Dastugue, B. & Jagla, K. Coordinated development of muscles and tendons of the Drosophila leg. *Development* **131**, 6041-6051 (2004).

24 Hartenstein, V. *Atlas of Drosophila development*. Vol. 328 (Cold Spring Harbor Laboratory Press, 1993).

25 Petersen, T. N., Brunak, S., von Heijne, G. & Nielsen, H. SignalP 4.0: discriminating signal peptides from transmembrane regions. *Nat Methods* **8**, 785-786 (2011).

26 Gramates, L. S. *et al.* FlyBase at 25: looking to the future. *Nucleic Acids Res* **45**, D663-D671 (2017).

27 Bendtsen, J. D., Jensen, L. J., Blom, N., Von Heijne, G. & Brunak, S. Feature-based prediction of non-classical and leaderless protein secretion. *Protein Eng Des Sel* **17**, 349-356 (2004).

28 Wang, M. *et al.* PaxDb, a database of protein abundance averages across all three domains of life. *Mol Cell Proteomics* **11**, 492-500 (2012).

29 Chintapalli, V. R., Wang, J. & Dow, J. A. Using FlyAtlas to identify better Drosophila melanogaster models of human disease. *Nat Genet* **39**, 715-720 (2007).

30 Brown, G. R. *et al.* Gene: a gene-centered information resource at NCBI. *Nucleic Acids Res* **43**, D36-D42 (2014).

31 Eaton, S. L. *et al.* Total protein analysis as a reliable loading control for quantitative fluorescent Western blotting. *PLoS ONE* **8**, e72457 (2013).

32 Armenteros, J. J. A. *et al.* SignalP 5.0 improves signal peptide predictions using deep neural networks. *Nat Biotechnol* **37**, 420-423 (2019).

33 Pfeiffer, B. D. *et al.* Refinement of tools for targeted gene expression in Drosophila. *Genetics* **186**, 735-755 (2010).

34 Lee, T. & Luo, L. Mosaic analysis with a repressible cell marker for studies of gene function in neuronal morphogenesis. *Neuron* **22**, 451-461 (1999).

35 Park, S. *et al.* A genetic strategy to measure circulating Drosophila insulin reveals genes regulating insulin production and secretion. *PLoS Genet* **10**, e1004555 (2014).

36 Brankatschk, M. & Eaton, S. Lipoprotein particles cross the blood–brain barrier in Drosophila. *J Neurosci* **30**, 10441-10447 (2010).

37 Palm, W. *et al.* Lipoproteins in Drosophila melanogaster—assembly, function, and influence on tissue lipid composition. *PLoS Genet* **8**, e1002828 (2012).

38 Schuster, C. M., Davis, G. W., Fetter, R. D. & Goodman, C. S. Genetic dissection of structural and functional components of synaptic plasticity. I. Fasciclin II controls synaptic stabilization and growth. *Neuron* **17**, 641-654 (1996).

39 Demontis, F. & Perrimon, N. FOXO/4E-BP signaling in Drosophila muscles regulates organism-wide proteostasis during aging. *Cell* **143**, 813-825 (2010).

40 Ranganayakulu, G., Schulz, R. A. & Olson, E. N. Wingless Signaling InducesnautilusExpression in the Ventral Mesoderm of theDrosophilaEmbryo. *Dev Biol* **176**, 143-148 (1996).

41 Bischof, J. *et al.* A versatile platform for creating a comprehensive UAS-ORFeome library in Drosophila. *Development* **140**, 2434-2442 (2013).

42 Perkins, L. A. *et al.* The transgenic RNAi project at Harvard Medical School: resources and validation. *Genetics* **201**, 843-852 (2015).

43 Dietzl, G. *et al.* A genome-wide transgenic RNAi library for conditional gene inactivation in Drosophila. *Nature* **448**, 151 (2007).

44 Hu, Y. *et al.* An integrative approach to ortholog prediction for disease-focused and other functional studies. *BMC Bioinformatics* **12**, 357 (2011).

45 Hyun, S. *et al.* Conserved MicroRNA miR-8/miR-200 and its target USH/FOG2 control growth by regulating PI3K. *Cell* **139**, 1096-1108 (2009).

46 Lee, G., Jun, J. & Hyun, S. MicroRNA miR-8 regulates multiple growth factor hormones produced from Drosophila fat cells. *Insect Mol Biol* **24**, 311-318 (2015).

47 Zhu, Q. *et al.* Domain organization and phylogenetic analysis of the chitinase-like family of proteins in three species of insects. *Insect Biochem Mol Biol* **38**, 452-466 (2008).

48 Pesch, Y.-Y., Riedel, D., Patil, K. R., Loch, G. & Behr, M. Chitinases and Imaginal disc growth factors organize the extracellular matrix formation at barrier tissues in insects. *Sci Rep* **6** (2016).

49 Kawamura, K., Shibata, T., Saget, O., Peel, D. & Bryant, P. J. A new family of growth factors produced by the fat body and active on Drosophila imaginal disc cells. *Development* **126**, 211-219 (1999).

50 Varela, P. F., Llera, A. S., Mariuzza, R. A. & Tormo, J. Crystal structure of imaginal disc growth factor-2: a member of a new family of growth-promoting glycoproteins from Drosophila melanogaster. *J Biol Chem* (2002).

51 Zurovec, M., Dolezal, T., Gazi, M., Pavlova, E. & Bryant, P. J. Adenosine deaminase-related growth factors stimulate cell proliferation in Drosophila by depleting extracellular adenosine. *Proc Natl Acad Sci* **99**, 4403-4408 (2002).

52 Zuberova, M., Fenckova, M., Simek, P., Janeckova, L. & Dolezal, T. Increased extracellular adenosine in Drosophila that are deficient in adenosine deaminase activates a release of energy stores leading to wasting and death. *Dis Model Mech* **3**, 773-784 (2010).

53 Chanana, B., Graf, R., Koledachkina, T., Pflanz, R. & Vorbrüggen, G. α PS2 integrin-mediated muscle attachment in Drosophila requires the ECM protein Thrombospondin. *Mech Dev* **124**, 463-475 (2007).

54 Subramanian, A., Wayburn, B., Bunch, T. & Volk, T. Thrombospondin-mediated adhesion is essential for the formation of the

myotendinous junction in Drosophila. *Development* **134**, 1269-1278 (2007).

55 Pundir, S., Martin, M. J. & O'Donovan, C. Uniprot protein knowledgebase. *Protein Bioinformatics: From Protein Modifications and Networks to Proteomics*, 41-55 (2017).

56 Ashburner, M. *et al.* Gene Ontology: tool for the unification of biology. *Nat Genet* **25**, 25 (2000).

57 Zhu, B. *et al.* Drosophila neurotrophins reveal a common mechanism for nervous system formation. *PLoS Biol* **6**, e284 (2008).

58 Schneider, D. S., Jin, Y., Morisato, D. & Anderson, K. V. A processed form of the Spatzle protein defines dorsal-ventral polarity in the Drosophila embryo. *Development* **120**, 1243-1250 (1994).

59 Serpe, M., Ralston, A., Blair, S. S. & O'Connor, M. B. Matching catalytic activity to developmental function: tolloid-related processes Sog in order to help specify the posterior crossvein in the Drosophila wing. *Development* **132**, 2645-2656 (2005).

60 Ralston, A. & Blair, S. S. Long-range Dpp signaling is regulated to restrict BMP signaling to a crossvein competent zone. *Dev Biol* **280**, 187-200 (2005).

61 Levashina, E. A. *et al.* Constitutive activation of toll-mediated antifungal defense in serpin-deficient Drosophila. *Science* **285**, 1917-1919 (1999).

62 Charron, Y. *et al.* The serpin Spn5 is essential for wing expansion in Drosophila melanogaster. *Int J Dev Biol* **52**, 933-942 (2004).

63 Ahmad, S. T., Sweeney, S. T., Lee, J.-A., Sweeney, N. T. & Gao, F.-B. Genetic screen identifies serpin5 as a regulator of the toll pathway and CHMP2B toxicity associated with frontotemporal dementia. *Proc Natl Acad Sci* **106**, 12168-12173 (2009).

64 Rylett, C. M., Walker, M. J., Howell, G. J., Shirras, A. D. & Isaac, R. E. Male accessory glands of Drosophila melanogaster make a secreted angiotensin I-converting enzyme (ANCE), suggesting a role for the peptide-processing enzyme in seminal fluid. *J Exp Biol* **210**, 3601-3606 (2007).

65 Parsons, B. & Foley, E. The Drosophila platelet-derived growth factor and vascular endothelial growth factor-receptor related (Pvr) protein ligands Pvf2 and Pvf3 control hemocyte viability and invasive migration. *J Biol Chem* **288**, 20173-20183 (2013).

66 Wright, V. M., Vogt, K. L., Smythe, E. & Zeidler, M. P. Differential activities of the Drosophila JAK/STAT pathway ligands Upd, Upd2 and Upd3. *Cell Signal* **23**, 920-927 (2011).

67 Brunner, E. *et al.* A high-quality catalog of the Drosophila melanogaster proteome. *Nat Biotechnol* **25**, 576 (2007).

68 Agrawal, N. *et al.* The Drosophila TNF Eiger is an adipokine that acts on insulin-producing cells to mediate nutrient response. *Cell Metab* **23**, 675-684 (2016).

69 Song, W., Veenstra, J. A. & Perrimon, N. Control of lipid metabolism by tachykinin in Drosophila. *Cell Rep* **9**, 40-47 (2014).

70 Hansen, K. K., Hauser, F., Williamson, M., Weber, S. B. & Grimmelikhuijzen, C. J. The Drosophila genes CG14593 and CG30106 code for G-protein-coupled receptors specifically activated by the neuropeptides CCHamide-1 and CCHamide-2. *Biochem Biophys Res Commun* **404**, 184-189 (2011).

71 Okamoto, N. *et al.* A secreted decoy of InR antagonizes insulin/IGF signaling to restrict body growth in Drosophila. *Genes Dev* **27**, 87-97 (2013).

72 Koyama, T. & Mirth, C. K. Growth-blocking peptides as nutrition-sensitive signals for insulin secretion and body size regulation. *PLoS Biol* **14**, e1002392 (2016).

73 Takehana, A. *et al.* Peptidoglycan recognition protein (PGRP)-LE and PGRP-LC act synergistically in Drosophila immunity. *EMBO J* **23**, 4690-4700 (2004).

74 Nguyen, M., Parker, L. & Arora, K. Identification of maverick, a novel member of the TGF-β superfamily in Drosophila. *Mech Dev* **95**, 201-206 (2000).

75 Deutsch, J. *et al.* Larval fat body-specific gene expression in D. melanogaster. *Genesis* **10**, 220-231 (1989).

76 Roberts, D. B., Turing, J. D. & Loughlin, S. A. The advantages that accrue to Drosophila melanogaster possessing larval serum protein 1. *J Insect Physiol* **37**, 391399-397400 (1991).

77 Handke, B. *et al.* The hemolymph proteome of fed and starved Drosophila larvae. *PLoS ONE* **8**, e67208 (2013).

78 Telfer, W. H. & Kunkel, J. G. The function and evolution of insect storage hexamers. *Annu Rev Entomol* **36**, 205-228 (1991).

79 Okada, H., Ebhardt, H. A., Vonesch, S. C., Aebersold, R. & Hafen, E. Proteome-wide association studies identify biochemical modules associated with a wing-size phenotype in Drosophila melanogaster. *Nat Commun* **7** (2016).

80 Green, N. *et al.* A Common Suite of Coagulation Proteins Function in Drosophila

Muscle Attachment. *Genetics* **204**, 1075-1087 (2016).
81 Kutty, R. K. *et al.* Molecular Characterization and Developmental Expression of a Retinoid- and Fatty Acid-binding Glycoprotein from Drosophila A PUTATIVE LIPOPHORIN. *J Biol Chem* **271**, 20641-20649 (1996).
82 Panáková, D., Sprong, H., Marois, E., Thiele, C. & Eaton, S. Lipoprotein particles are required for Hedgehog and Wingless signalling. *Nature* **435**, 58-65 (2005).
83 Callejo, A., Culi, J. & Guerrero, I. Patched, the receptor of Hedgehog, is a lipoprotein receptor. *Proc Natl Acad Sci* **105**, 912-917 (2008).
84 Palm, W. *et al.* Secretion and signaling activities of lipoprotein-associated hedgehog and non-sterol-modified hedgehog in flies and mammals. *PLoS Biol* **11**, e1001505 (2013).
85 Rodenfels, J. *et al.* Production of systemically circulating Hedgehog by the intestine couples nutrition to growth and development. *Genes Dev* **28**, 2636-2651 (2014).
86 Parra-Peralbo, E. & Culi, J. Drosophila lipophorin receptors mediate the uptake of neutral lipids in oocytes and imaginal disc cells by an endocytosis-independent mechanism. *PLoS Genet* **7**, e1001297 (2011).
87 Rodríguez-Vázquez, M., Vaquero, D., Parra-Peralbo, E., Mejía-Morales, J. E. & Culi, J. Drosophila lipophorin receptors recruit the lipoprotein LTP to the plasma membrane to mediate lipid uptake. *PLoS Genet* **11**, e1005356 (2015).
88 Fleischmannova, J. *et al.* Differential response of Drosophila cell lines to extracellular adenosine. *Insect Biochem Mol Biol* **42**, 321-331 (2012).
89 Dolezelova, E., Nothacker, H.-P., Civelli, O., Bryant, P. J. & Zurovec, M. A Drosophila adenosine receptor activates cAMP and calcium signaling. *Insect Biochem Mol Biol* **37**, 318-329 (2007).
90 Dolezal, T., Gazi, M., Zurovec, M. & Bryant, P. J. Genetic analysis of the ADGF multigene family by homologous recombination and gene conversion in Drosophila. *Genetics* **165**, 653-666 (2003).
91 Zavialov, A. V. & Engström, Å. Human ADA2 belongs to a new family of growth factors with adenosine deaminase activity. *Biochem J* **391**, 51-57 (2005).
92 Strassburger, K. *et al.* Oxygenation and adenosine deaminase support growth and proliferation of ex vivo cultured Drosophila wing imaginal discs. *Development*, dev. 147538 (2017).
93 Chen, J. *et al.* Crossveinless d is a vitellogenin-like lipoprotein that binds BMPs and HSPGs, and is required for normal BMP signaling in the Drosophila wing. *Development* **139**, 2170-2176 (2012).
94 Jaramillo, M. S., Lovato, C. V., Baca, E. M. & Cripps, R. M. Crossveinless and the TGFβ pathway regulate fiber number in the Drosophila adult jump muscle. *Development* **136**, 1105-1113 (2009).
95 Fogerty, F. J. *et al.* Tiggrin, a novel Drosophila extracellular matrix protein that functions as a ligand for Drosophila alpha PS2 beta PS integrins. *Development* **120**, 1747-1758 (1994).
96 Gotwals, P. J., Fessler, L. I., Wehrli, M. & Hynes, R. O. Drosophila PS1 integrin is a laminin receptor and differs in ligand specificity from PS2. *Proc Natl Acad Sci* **91**, 11447-11451 (1994).
97 Bunch, T. A. *et al.* The PS2 integrin ligand tiggrin is required for proper muscle function in Drosophila. *Development* **125**, 1679-1689 (1998).
98 Carhan, A., Tang, K., Shirras, C. A., Shirras, A. D. & Isaac, R. E. Loss of Angiotensin-converting enzyme-related (ACER) peptidase disrupts night-time sleep in adult Drosophila melanogaster. *J Exp Biol* **214**, 680-686 (2011).
99 Liao, F.-T., Chang, C.-Y., Su, M.-T. & Kuo, W.-C. Necessity of angiotensin-converting enzyme-related gene for cardiac functions and longevity of Drosophila melanogaster assessed by optical coherence tomography. *J Biomed Opt* **19**, 011014-011014 (2014).
100 Gorzelniak, K., Engeli, S., Janke, J., Luft, F. C. & Sharma, A. M. Hormonal regulation of the human adipose-tissue renin–angiotensin system: relationship to obesity and hypertension. *J Hypertens* **20**, 965-973 (2002).
101 Jayasooriya, A. P. *et al.* Mice lacking angiotensin-converting enzyme have increased energy expenditure, with reduced fat mass and improved glucose clearance. *Proc Natl Acad Sci* **105**, 6531-6536 (2008).
102 Gupte, M. *et al.* ACE2 is expressed in mouse adipocytes and regulated by a high-fat diet. *Am J Physiol Regul Integr Comp Physiol* **295**, R781-R788 (2008).
103 Engeli, S. *et al.* The adipose-tissue renin–angiotensin–aldosterone system: role in the metabolic syndrome? *Int J Biochem Cell Biol* **35**, 807-825 (2003).
104 Igboin, C. O., Griffen, A. L. & Leys, E. J. The Drosophila melanogaster host model. *J Oral Microbiol* **4**, 10368 (2012).

105 Gottar, M. *et al.* Dual detection of fungal infections in Drosophila via recognition of glucans and sensing of virulence factors. *Cell* **127**, 1425-1437 (2006).
106 Arefin, B. *et al.* Genome-wide transcriptional analysis of Drosophila larvae infected by entomopathogenic nematodes shows involvement of complement, recognition and extracellular matrix proteins. *J Innate Immun* **6**, 192-204 (2014).
107 Mulinari, S., Häcker, U. & Castillejo-López, C. Expression and regulation of Spätzle-processing enzyme in Drosophila. *FEBS Lett* **580**, 5406-5410 (2006).
108 Jang, I.-H. *et al.* A Spätzle-processing enzyme required for toll signaling activation in Drosophila innate immunity. *Dev Cell* **10**, 45-55 (2006).
109 Ekengren, S. *et al.* A humoral stress response in Drosophila. *Curr Biol* **11**, 714-718 (2001).
110 Ekengren, S. & Hultmark, D. A family of Turandot-related genes in the humoral stress response of Drosophila. *Biochem Biophys Res Commun* **284**, 998-1003 (2001).
111 Mahapatra, C. T. & Rand, M. D. Methylmercury tolerance is associated with the humoral stress factor gene Turandot A. *Neurotoxicol Teratol* **34**, 387-394 (2012).
112 Agaisse, H., Petersen, U.-M., Boutros, M., Mathey-Prevot, B. & Perrimon, N. Signaling role of hemocytes in Drosophila JAK/STAT-dependent response to septic injury. *Dev Cell* **5**, 441-450 (2003).
113 Tang, H., Kambris, Z., Lemaitre, B. & Hashimoto, C. Two proteases defining a melanization cascade in the immune system of Drosophila. *J Biol Chem* **281**, 28097-28104 (2006).
114 Castillejo-López, C. & Häcker, U. The serine protease Sp7 is expressed in blood cells and regulates the melanization reaction in Drosophila. *Biochem Biophys Res Commun* **338**, 1075-1082 (2005).
115 Drechsler, M., Schmidt, A. C., Meyer, H. & Paululat, A. The conserved ADAMTS-like protein lonely heart mediates matrix formation and cardiac tissue integrity. *PLoS Genet* **9**, e1003616 (2013).
116 González-Morales, N., Mendoza-Ortíz, M. Á., Blowes, L. M., Missirlis, F. & Riesgo-Escovar, J. R. Ferritin Is required in multiple tissues during Drosophila melanogaster development. *PLoS ONE* **10**, e0133499 (2015).
117 Tang, X. & Zhou, B. Ferritin is the key to dietary iron absorption and tissue iron detoxification in Drosophila melanogaster. *The FASEB Journal* **27**, 288-298 (2013).
118 Theopold, U. *et al.* Helix pomatia lectin, an inducer of Drosophila immune response, binds to hemomucin, a novel surface mucin. *J Biol Chem* **271**, 12708-12715 (1996).
119 Kolkhof, P. *et al.* A luciferase-fragment complementation assay to detect lipid droplet-associated protein-protein interactions. *Mol Cell Proteomics* **16**, 329-345 (2017).
120 Coates, D. *et al.* Functional conservation of the active sites of human and Drosophila angiotensin I-converting enzyme. *Biochemistry (Mosc)* **39**, 8963-8969 (2000).
121 Didelot, G. *et al.* Tequila, a neurotrypsin ortholog, regulates long-term memory formation in Drosophila. *Science* **313**, 851-853 (2006).
122 Chen, C.-C. *et al.* Visualizing long-term memory formation in two neurons of the Drosophila brain. *Science* **335**, 678-685 (2012).
123 Colomb, J., Kaiser, L., Chabaud, M. A. & Preat, T. Parametric and genetic analysis of Drosophila appetitive long-term memory and sugar motivation. *Genes, Brain and Behavior* **8**, 407-415 (2009).
124 Huang, C.-W. *et al.* Tequila regulates insulin-like signaling and extends life span in Drosophila melanogaster. *J Gerontol A Biol Sci Med Sci* **70**, 1461-1469 (2015).
125 Molinari, F. *et al.* Truncating neurotrypsin mutation in autosomal recessive nonsyndromic mental retardation. *Science* **298**, 1779-1781 (2002).
126 Mitsui, S. *et al.* A mental retardation gene, motopsin/neurotrypsin/prss12, modulates hippocampal function and social interaction. *Eur J Neurosci* **30**, 2368-2378 (2009).
127 Baggerman, G., Cerstiaens, A., De Loof, A. & Schoofs, L. Peptidomics of the larval Drosophila melanogastercentral nervous system. *J Biol Chem* **277**, 40368-40374 (2002).
128 Yew, J. Y. *et al.* Analysis of neuropeptide expression and localization in adult Drosophila melanogaster central nervous system by affinity cell-capture mass spectrometry. *J Proteome Res* **8**, 1271-1284 (2009).
129 Nässel, D. R. & Winther, Å. M. Drosophila neuropeptides in regulation of physiology and behavior. *Prog Neurobiol* **92**, 42-104 (2010).
130 Levashina, E. A. *et al.* Conserved role of a complement-like protein in phagocytosis revealed by dsRNA knockout in cultured cells of the mosquito, Anopheles gambiae. *Cell* **104**, 709-718 (2001).
131 Shokal, U., Kopydlowski, H. & Eleftherianos, I. The distinct function of Tep2 and Tep6 in the

immune defense of Drosophila melanogaster against the pathogen Photorhabdus. *Virulence*, 00-00 (2017).

132 Aoun, R. B. *et al.* Analysis of thioester-containing proteins during the innate immune response of Drosophila melanogaster. *J Innate Immun* **3**, 52-64 (2011).

133 Li, C. *et al.* Soluble CD109 binds TGF-β and antagonizes TGF-β signalling and responses. *Biochem J* **473**, 537-537 (2016).

134 Sakakura, H. *et al.* Detection of a soluble form of CD109 in serum of CD109 transgenic and tumor xenografted mice. *PLoS ONE* **9**, e83385 (2014).

135 Hagiwara, S. *et al.* Processing of CD109 by furin and its role in the regulation of TGF-[beta] signaling. *Oncogene* **29**, 2181 (2010).

136 Litvinov, I. V. *et al.* CD109 release from the cell surface in human keratinocytes regulates TGF-β receptor expression, TGF-β signalling and STAT3 activation: relevance to psoriasis. *Exp Dermatol* **20**, 627-632 (2011).

137 Serpe, M. & O'Connor, M. B. The metalloprotease tolloid-related and its TGF-β-like substrate Dawdle regulate Drosophila motoneuron axon guidance. *Development* **133**, 4969-4979 (2006).

138 Droujinine, I. A. & Perrimon, N. Interorgan Communication Pathways in Physiology: Focus on Drosophila. *Annu Rev Genet* **50**, 539-570 (2016).

139 Casalena, G., Daehn, I. & Bottinger, E. in *Semin Nephrol.* 295-303 (Elsevier).

140 Song, W. *et al.* Activin signaling mediates muscle-to-adipose communication in a mitochondria dysfunction-associated obesity model. *Proc Natl Acad Sci* **114**, 8596-8601 (2017).

141 Ge, G., Hopkins, D. R., Ho, W.-B. & Greenspan, D. S. GDF11 forms a bone morphogenetic protein 1-activated latent complex that can modulate nerve growth factor-induced differentiation of PC12 cells. *Mol Cell Biol* **25**, 5846-5858 (2005).

142 Wolfman, N. M. *et al.* Activation of latent myostatin by the BMP-1/tolloid family of metalloproteinases. *Proc Natl Acad Sci* **100**, 15842-15846 (2003).

143 Buchon, N. *et al.* A single modular serine protease integrates signals from pattern-recognition receptors upstream of the Drosophila Toll pathway. *Proc Natl Acad Sci* **106**, 12442-12447 (2009).

144 Khurana, V. *et al.* Lysosomal dysfunction promotes cleavage and neurotoxicity of tau in vivo. *PLoS Genet* **6**, e1001026 (2010).

145 Myllykangas, L. *et al.* Cathepsin D-deficient Drosophila recapitulate the key features of neuronal ceroid lipofuscinoses. *Neurobiol Dis* **19**, 194-199 (2005).

146 Benes, P., Vetvicka, V. & Fusek, M. Cathepsin D—many functions of one aspartic protease. *Crit Rev Oncol Hematol* **68**, 12-28 (2008).

147 Vetvicka, V., Vetvickova, J. & Fusek, M. Effect of procathepsin D and its activation peptide on prostate cancer cells. *Cancer Lett* **129**, 55-59 (1998).

148 Perchick, G. B. & Jabbour, H. N. Cyclooxygenase-2 overexpression inhibits cathepsin D-mediated cleavage of plasminogen to the potent antiangiogenic factor angiostatin. *Endocrinology* **144**, 5322-5328 (2003).

149 Conover, C. A., Perry, J. E. & Tindall, D. J. Endogenous cathepsin D-mediated hydrolysis of insulin-like growth factor-binding proteins in cultured human prostatic carcinoma cells. *J Clin Endocrinol Metab* **80**, 987-993 (1995).

150 Tyynelä, J. *et al.* A mutation in the ovine cathepsin D gene causes a congenital lysosomal storage disease with profound neurodegeneration. *EMBO J* **19**, 2786-2792 (2000).

151 Steinfeld, R. *et al.* Cathepsin D deficiency is associated with a human neurodegenerative disorder. *Am J Hum Genet* **78**, 988-998 (2006).

152 Tanentzapf, G., Devenport, D., Godt, D. & Brown, N. H. Integrin-dependent anchoring of a stem cell niche. *Nat Cell Biol* **9**, 1413 (2007).

153 Miosge, N., Holzhausen, S., Zelent, C., Sprysch, P. & Herken, R. Nidogen-1 and nidogen-2 are found in basement membranes during human embryonic development. *Histochem J* **33**, 523-530 (2001).

154 Davis, R. A. Cell and molecular biology of the assembly and secretion of apolipoprotein B-containing lipoproteins by the liver. *Biochimica et Biophysica Acta (BBA)-Molecular and Cell Biology of Lipids* **1440**, 1-31 (1999).

155 Fagerberg, L. *et al.* Analysis of the human tissue-specific expression by genome-wide integration of transcriptomics and antibody-based proteomics. *Mol Cell Proteomics* **13**, 397-406 (2014).

156 Sinha, M. *et al.* Restoring systemic GDF11 levels reverses age-related dysfunction in mouse skeletal muscle. *Science* **344**, 649-652 (2014).

157 Egerman, M. A. *et al.* GDF11 increases with age and inhibits skeletal muscle regeneration. *Cell Metab* **22**, 164-174 (2015).

158 Landau, R. *et al.* Alteration of circulating Placental Leucine Aminopeptidase (P-LAP) activity in preeclampsia. *Neuro Endocrinol Lett* **31**, 63-66 (2009).

159 Lew, R. A. *et al.* Angiotensin AT4 ligands are potent, competitive inhibitors of insulin regulated aminopeptidase (IRAP). *J Neurochem* **86**, 344-350 (2003).

160 Matsumoto, H. *et al.* Characterization of a recombinant soluble form of human placental leucine aminopeptidase/oxytocinase expressed in Chinese hamster ovary cells. *Eur J Biochem* **267**, 46-52 (2000).

161 Matsumoto, H. *et al.* Expression of placental leucine aminopeptidase/oxytocinase in neuronal cells and its action on neuronal peptides. *Eur J Biochem* **268**, 3259-3266 (2001).

162 Herbst, J. J. *et al.* Insulin stimulates cell surface aminopeptidase activity toward vasopressin in adipocytes. *Am J Physiol* **272**, E600-E606 (1997).

163 Hermans, S. J. *et al.* Crystal structure of human insulin-regulated aminopeptidase with specificity for cyclic peptides. *Protein Sci* **24**, 190-199 (2015).

164 Niwa, M. *et al.* IRAP deficiency attenuates diet-induced obesity in mice through increased energy expenditure. *Biochem Biophys Res Commun* **457**, 12-18 (2015).

165 Schomburg, L. *et al.* Human TRH-degrading ectoenzyme. *Eur J Biochem* **265**, 415-422 (1999).

166 Principe, S. *et al.* In-depth proteomic analyses of exosomes isolated from expressed prostatic secretions in urine. *Proteomics* **13**, 1667-1671 (2013).

167 Prunotto, M. *et al.* Proteomic analysis of podocyte exosome-enriched fraction from normal human urine. *J Proteomics* **82**, 193-229 (2013).

168 Tsujimoto, M., Goto, Y., Maruyama, M. & Hattori, A. Biochemical and enzymatic properties of the M1 family of aminopeptidases involved in the regulation of blood pressure. *Heart Fail Rev* **13**, 285-291 (2008).

169 Kuruppu, S., Rajapakse, N. W., Dunstan, R. A. & Smith, A. I. Nitric oxide inhibits the production of soluble endothelin converting enzyme-1. *Mol Cell Biochem* **396**, 49-54 (2014).

170 Kuruppu, S., Reeve, S. & Ian Smith, A. Characterisation of endothelin converting enzyme-1 shedding from endothelial cells. *FEBS Lett* **581**, 4501-4506 (2007).

171 Kuruppu, S. & Smith, A. I. Endothelin Converting Enzyme-1 phosphorylation and trafficking. *FEBS Lett* **586**, 2212-2217 (2012).

172 Ohnaka, K., Takayanagi, R., Nishikawa, M., Haji, M. & Nawata, H. Purification and characterization of a phosphoramidon-sensitive endothelin-converting enzyme in porcine aortic endothelium. OFF. *J Biol Chem* **268**, 26759-26766 (1993).

173 Bayés-Genís, A. *et al.* Soluble neprilysin is predictive of cardiovascular death and heart failure hospitalization in heart failure patients. *J Am Coll Cardiol* **65**, 657-665 (2015).

174 Bayes-Genis, A., Barallat, J. & Richards, A. M. A test in context: neprilysin: function, inhibition, and biomarker. *J Am Coll Cardiol* **68**, 639-653 (2016).

175 Bhoola, K., Figueroa, C. & Worthy, K. Bioregulation of kinins: kallikreins, kininogens, and kininases. *Pharmacol Rev* **44**, 1-80 (1992).

176 MacKenzie, J. A., Roosa, K. A., Gump, B. B., Dumas, A. K. & Bendinskas, K. G. Plasma prekallikrein levels are positively associated with circulating lipid levels and the metabolic syndrome in children. *Appl Physiol Nutr Metab* **35**, 518-525 (2010).

177 Selvarajan, S., Lund, L. R., Takeuchi, T., Craik, C. S. & Werb, Z. A plasma kallikrein-dependent plasminogen cascade required for adipocyte differentiation. *Nat Cell Biol* **3**, 267 (2001).

178 Grundmann, U., Römisch, J., Siebold, B., Bohn, H. & Amann, E. Cloning and expression of a cDNA encoding human placental protein 11, a putative serine protease with diagnostic significance as a tumor marker. *DNA Cell Biol* **9**, 243-250 (1990).

179 Laneve, P. *et al.* The tumor marker human placental protein 11 is an endoribonuclease. *J Biol Chem* **283**, 34712-34719 (2008).

180 Poe, J. C. *et al.* EndoU is a novel regulator of AICD during peripheral B cell selection. *J Exp Med* **211**, 57-69 (2014).

181 Rafnar, T. *et al.* Sequence variants at the TERT-CLPTM1L locus associate with many cancer types. *Nat Genet* **41**, 221-227 (2009).

182 James, M. A. *et al.* Functional characterization of CLPTM1L as a lung cancer risk candidate gene in the 5p15. 33 locus. *PLoS ONE* **7**, e36116 (2012).

183 Jia, J. *et al.* CLPTM1L promotes growth and enhances aneuploidy in pancreatic cancer cells. *Cancer Res* **74**, 2785-2795 (2014).

184 Yamamoto, K., Okamoto, A., Isonishi, S., Ochiai, K. & Ohtake, Y. A novel gene, CRR9, which was up-regulated in CDDP-resistant

ovarian tumor cell line, was associated with apoptosis. *Biochem Biophys Res Commun* **280**, 1148-1154 (2001).

185 Nakayama, Y. *et al.* Cloning of cDNA encoding a regeneration-associated muscle protease whose expression is attenuated in cell lines derived from Duchenne muscular dystrophy patients. *Am J Pathol* **164**, 1773-1782 (2004).

186 Lo, P. H. Y., Tanikawa, C., Katagiri, T., Nakamura, Y. & Matsuda, K. Identification of novel epigenetically inactivated gene PAMR1 in breast carcinoma. *Oncol Rep* **33**, 267-273 (2015).

187 Badeanlou, L., Furlan-Freguia, C., Yang, G., Ruf, W. & Samad, F. Tissue factor-protease-activated receptor 2 signaling promotes diet-induced obesity and adipose inflammation. *Nat Med* **17**, 1490-1497 (2011).

188 Takahashi, N. *et al.* The production of coagulation factor VII by adipocytes is enhanced by tumor necrosis factor-[alpha] or isoproterenol. *Int J Obes* **39**, 747 (2015).

189 Initiative, I. S. C. Assessment of established techniques to determine developmental and malignant potential of human pluripotent stem cells. *Nat Commun* **9** (2018).
