# Supplementary material for: Proteomics of protein trafficking by in vivo tissue-specific labeling
Source: Nat Commun. 2021 Apr 22;12:2382. doi: 10.1038/s41467-021-22599-x (PMC8062696; doi:10.1038/s41467-021-22599-x)
Supplement: Supplementary file 2 — Description of Additional Supplementary Files [file 41467_2021_22599_MOESM2_ESM.doc]

**Description of additional supplementary files**

**File Name: Supplementary Data 1**

**Description:** BirA*G3-ER mass spectrometry data (in separate Excel attachment)

References: Manual hit categorization into broad categories using data from FlyBase^1^ and NCBI Gene^2^.

Comparison of hits to fly orthologs (DIOPT version 5.3 ^3^) of mammalian adipocyte^4-13^ and myocyte^14-21^ secretome data.

Signal peptide: ^22^

TM (transmembrane) helices: ^23^

DIOPT orthologs: ^3^

Abbreviations: vs., version (DIOPT).

**File Name: Supplementary Data 2**

**Description:** Total hemolymph mass spectrometry data (in separate Excel attachment)

**File Name: Supplementary Data 3**

**Description:** BirA*R118G-ER mass spectrometry data (in separate Excel attachment)

References: Comparison of hits to fly orthologs (DIOPT version 5.3 ^3^) of mammalian adipocyte^4-13^ and myocyte^14-21^ secretome data.

DIOPT orthologs: ^3^

Abbreviations: vs., version (DIOPT).

**File Name: Supplementary Data 4**

**Description:** Teratoma BirA*G3-ER mass spectrometry data (in separate Excel attachment). Two-sided two-sample t-test unadjusted *p*-values shown.

References: Mammalian adipocyte^4-13^ and myocyte^14-21^ secretome data.

Signal peptide: ^24^

TM (transmembrane) helices: ^23^

Mammalian blood (cells removed): ^25,26^

**File Name: Supplementary Data 5**

**Description:** Serum BirA*G3-ER mass spectrometry data (in separate Excel attachment). Two-sided two-sample t-test unadjusted *p*-values shown.

References: Mammalian adipocyte^4-13^ and myocyte^14-21^ secretome data.

Signal peptide: ^24^

TM (transmembrane) helices: ^23^

Mammalian blood (cells removed): ^25,26^

**File Name: Supplementary Data 6**

**Description:** Examples of interesting proteins identified in the teratoma-derived serum proteomics dataset (in separate Excel attachment)

**File Name: Supplementary Movie 1**

**Description:** *LPP-Gal4>*control (*attp*) (vial 1; left) versus *LPP-Gal4>CG2145-i-1* (vial2; right) climbing-ability assay at 3 weeks old and 29°C. Flies were tapped to the bottom of the vial at the beginning of the assay and then allowed to climb up the vial.

**File Name: Supplementary Movie 2**

**Description:** *LPP-Gal4>*control (*w-i*) (vial 1; left) versus *LPP-Gal4>CG4332-i-1* (vial 3; right) climbing-ability assay at 5 weeks old and 27°C. Flies were tapped to the bottom of the vial at the beginning of the assay and then allowed to climb up the vial.

**File Name: Supplementary Movie 3**

**Description:** *LPP-Gal4>*control (*w-i*) (vial 1; left) versus *LPP-Gal4>CG31326-i-3* (vial 2; right) climbing-ability assay at 5 weeks old and 27°C. Flies were tapped to the bottom of the vial at the beginning of the assay and then allowed to climb up the vial.

**Supplementary Files Captions References**

1 Gramates, L. S. *et al.* FlyBase at 25: looking to the future. *Nucleic Acids Res* **45**, D663-D671 (2017).

2 Brown, G. R. *et al.* Gene: a gene-centered information resource at NCBI. *Nucleic Acids Res* **43**, D36-D42 (2014).

3 Hu, Y. *et al.* An integrative approach to ortholog prediction for disease-focused and other functional studies. *BMC Bioinformatics* **12**, 357 (2011).

4 Alvarez-Llamas, G. *et al.* Characterization of the human visceral adipose tissue secretome. *Mol Cell Proteomics* **6**, 589-600 (2007).

5 Chen, X., Cushman, S. W., Pannell, L. K. & Hess, S. Quantitative Proteomic Analysis of the Secretory Proteins from Rat Adipose Cells Using a 2D Liquid Chromatography− MS/MS Approach. *J Proteome Res* **4**, 570-577 (2005).

6 Lehr, S. *et al.* Identification and validation of novel adipokines released from primary human adipocytes. *Mol Cell Proteomics* **11**, M111. 010504 (2012).

7 Lim, J.-M. *et al.* Defining the regulated secreted proteome of rodent adipocytes upon the induction of insulin resistance. *J Proteome Res* **7**, 1251-1263 (2008).

8 Molina, H. *et al.* Temporal profiling of the adipocyte proteome during differentiation using a five-plex SILAC based strategy. *J Proteome Res* **8**, 48-58 (2008).

9 Rosenow, A. *et al.* Identification of novel human adipocyte secreted proteins by using SGBS cells. *J Proteome Res* **9**, 5389-5401 (2010).

10 Wu, L. E. *et al.* Identification of fatty acid binding protein 4 as an adipokine that regulates insulin secretion during obesity. *Mol Metab* **3**, 465-473 (2014).

11 Zhong, J. *et al.* Temporal profiling of the secretome during adipogenesis in humans. *J Proteome Res* **9**, 5228-5238 (2010).

12 Zhou, H. *et al.* Quantitative analysis of secretome from adipocytes regulated by insulin. *Acta Biochim Biophys Sin* **41**, 910-921 (2009).

13 Zvonic, S. *et al.* Secretome of Primary Cultures of Human Adipose-derived Stem Cells Modulation of Serpins by Adipogenesis. *Mol Cell Proteomics* **6**, 18-28 (2007).

14 Bortoluzzi, S., Scannapieco, P., Cestaro, A., Danieli, G. A. & Schiaffino, S. Computational reconstruction of the human skeletal muscle secretome. *Proteins* **62**, 776-792 (2006).

15 Chan, C. X. a. *et al.* Identification of differentially regulated secretome components during skeletal myogenesis. *Mol Cell Proteomics* **10**, M110. 004804 (2011).

16 Deshmukh, A. S., Cox, J., Jensen, L. J., Meissner, F. & Mann, M. Secretome analysis of lipid-induced insulin resistance in skeletal muscle cells by a combined experimental and bioinformatics workflow. *J Proteome Res* **14**, 4885-4895 (2015).

17 Hartwig, S. *et al.* Secretome profiling of primary human skeletal muscle cells. *Biochim Biophys Acta* **1844**, 1011-1017 (2014).

18 Hittel, D. S., Berggren, J. R., Shearer, J., Boyle, K. & Houmard, J. A. Increased secretion and expression of myostatin in skeletal muscle from extremely obese women. *Diabetes* **58**, 30-38 (2009).

19 Norheim, F. *et al.* Proteomic identification of secreted proteins from human skeletal muscle cells and expression in response to strength training. *Am J Physiol* **301**, E1013-E1021 (2011).

20 Le Bihan, M.-C. *et al.* In-depth analysis of the secretome identifies three major independent secretory pathways in differentiating human myoblasts. *J Proteomics* **77**, 344-356 (2012).

21 Yoon, J. H. *et al.* Proteomic analysis of the palmitate-induced myotube secretome reveals involvement of the annexin a1-formyl peptide receptor 2 (fpr2) pathway in insulin resistance. *Mol Cell Proteomics* **14**, 882-892 (2015).

22 Petersen, T. N., Brunak, S., von Heijne, G. & Nielsen, H. SignalP 4.0: discriminating signal peptides from transmembrane regions. *Nat Methods* **8**, 785-786 (2011).

23 Krogh, A., Larsson, B., Von Heijne, G. & Sonnhammer, E. L. Predicting transmembrane protein topology with a hidden Markov model: application to complete genomes. *J Mol Biol* **305**, 567-580 (2001).

24 Armenteros, J. J. A. *et al.* SignalP 5.0 improves signal peptide predictions using deep neural networks. *Nat Biotechnol* **37**, 420-423 (2019).

25 Farrah, T. *et al.* A high-confidence human plasma proteome reference set with estimated concentrations in PeptideAtlas. *Mol Cell Proteomics* **10**, M110. 006353 (2011).

26 Liu, T. *et al.* High dynamic range characterization of the trauma patient plasma proteome. *Mol Cell Proteomics* **5**, 1899-1913 (2006).
