# Supplementary material for: Proteomics of protein trafficking by in vivo tissue-specific labeling
Source: Nat Commun. 2021 Apr 22;12:2382. doi: 10.1038/s41467-021-22599-x (PMC8062696; doi:10.1038/s41467-021-22599-x)
Supplement: Supplementary file 12 — Reporting Summary [file 41467_2021_22599_MOESM12_ESM.pdf]

nature research

Corresponding author(s): Ilia A. Droujinine and Norbert Perrimon

Last updated by author(s): Mar 5, 2021

Reporting Summary

Nature Research wishes to improve the reproducibility of the work that we publish. This form provides structure for consistency and transparency in reporting. For further information on Nature Research policies, see our Editorial Policies and the Editorial Policy Checklist.

Statistics

For all statistical analyses, confirm that the following items are present in the figure legend, table legend, main text, or Methods section.

n/a	Confirmed	
☐	☒	The exact sample size (*n*) for each experimental group/condition, given as a discrete number and unit of measurement
☐	☒	A statement on whether measurements were taken from distinct samples or whether the same sample was measured repeatedly
☐	☒	The statistical test(s) used AND whether they are one- or two-sided<br>*Only common tests should be described solely by name; describe more complex techniques in the Methods section.*
☐	☒	A description of all covariates tested
☐	☒	A description of any assumptions or corrections, such as tests of normality and adjustment for multiple comparisons
☐	☒	A full description of the statistical parameters including central tendency (e.g. means) or other basic estimates (e.g. regression coefficient) AND variation (e.g. standard deviation) or associated estimates of uncertainty (e.g. confidence intervals)
☐	☒	For null hypothesis testing, the test statistic (e.g. *F*, *t*, *r*) with confidence intervals, effect sizes, degrees of freedom and *P* value noted<br>*Give P values as exact values whenever suitable.*
☒	☐	For Bayesian analysis, information on the choice of priors and Markov chain Monte Carlo settings
☒	☐	For hierarchical and complex designs, identification of the appropriate level for tests and full reporting of outcomes
☒	☐	Estimates of effect sizes (e.g. Cohen's *d*, Pearson's *r*), indicating how they were calculated

Our web collection on statistics for biologists contains articles on many of the points above.

Software and code

Policy information about availability of computer code

Data collection	See Materials and Methods for detailed descriptions. Typhoon Trio Imager (GE Healthcare Life Sciences) was used to acquire Flamingo-stained images. Biorad Chemidoc MP software (ImageLab Touch 2.4) was used to acquire chemiluminescent blot images in Supplementary Figure 13c-d. Mouse fluorescent blots were acquired using Li-Cor Odyssey Clx. For ELISAs, Nanodrop software (ND8000 version 2.2.0), or Spectramax Paradigm (Molecular Devices; Softmax Pro 6.2) software was used. For MS analysis, The Q Exactive HF-X or Q Exactive Plus were operated in the data-dependent mode acquiring HCD MS/MS scans (r = 15,000 for HF-X, 17,500 for QE+) after each MS1 scan (r = 60,000 for HF-X, 70,000 for QE+) on the top 12 (QE+) or top 20 (HF-X) most abundant ions using an MS1 target of 3 × 106 and an MS2 target of 5 × 104. The maximum ion time utilized for MS/MS scans was 120 ms; the HCD-normalized collision energy was set to 31 (HF-X) or 28 (QE+); the dynamic exclusion time was set to 20 s, and the peptide match and isotope exclusion functions were enabled. Charge exclusion was enabled for charge states that were unassigned, 1 and >7. For hemolymph MS, Peptides were analyzed by positive ion mode liquid chromatography tandem mass spectrometry (LC-MS/MS) using a high resolution hybrid Orbitrap Elite mass spectrometer (Thermo Fisher Scientific) via CID with data-dependent analysis (DDA) using a Top 5 approach (1 full FT-MS scan followed by 5 MS/MS CID scans). Maximum injection time was 50 msec for MS and 100 msec for MS/MS with 1 microscan for both modes. Isolation width was 2.3 Da and dynamic exclusion time was set to 90 sec. Peptides were delivered and separated using an EASY-nLC I nanoflow HPLC (Thermo Fisher Scientific) at 300 nL/min using self-packed 15 cm length × 75 µ m i.d. C18 fritted microcapillary Picofrit columns (New Objective). ZEN2012 software (Carl Zeiss) was used for fly confocal imaging. Mouse images were acquired on Leica SP8 confocal microscope. Histology samples were scanned at high resolution (10X) on a Zeiss Axio Scan.Z1 Slide Scanner to generate high-resolution tiled image files of the tissue section.
Data analysis	See Materials and Methods for detailed descriptions. The integrated density of bands in Fig. 1d' was measured using Adobe Photoshop CC2019 on inverted 8-bit grayscale images. Western blot analysis done on ImageStudio (Version 5.2.5) and Empiria (Version 1.3.0.83). Total protein stain images of each blot were used to normalize biotinylation (streptavidin) signal intensity in R. ggplot2 was used to visualize normalized biotinylated protein signal. All protein trafficking MS data were analyzed using Spectrum Mill software package v 7.00 (mouse data) or 6.1 (Drosophila data) pre-release (Agilent Technologies). Similar MS/MS spectra acquired on the same precursor m/z within +/- 60 s were merged. MS/MS spectra were excluded from searching if they were not within the precursor MH+ range of -600-6000 Da (mouse data) or 750-4000 Da (Drosophila data) or if they failed the quality filter by not having a sequence tag length >0. MS/MS spectra were searched

April 2020

against either all Drosophila melanogaster or mouse proteins (46,519 (mouse) or 21,979 (Drosophila) proteins, and 264 (mouse) or 259 (Drosophila) common contaminants). All spectra were allowed +/- 20 ppm mass tolerance for precursor and product ions, 30% (Drosophila) or 40% (mouse) minimum matched peak intensity, and “trypsin allow P” enzyme specificity with up to 2 missed cleavages. The fixed modifications were carbamidomethylation at cysteine, and TMT6 at N-termini and internal lysine residues. Variable modifications included oxidized methionine and N-terminal protein acetylation and deamination (mouse only). Individual spectra were automatically designated as confidently assigned using the Spectrum Mill autovalidation module. Specifically, a target-decoy based false-discovery rate (FDR) scoring threshold criteria via a two-step auto threshold strategy at the spectral and protein levels was used. First, peptide mode was set to allow automatic variable range precursor mass filtering with score thresholds optimized to yield a spectral level FDR of <1.2%. A protein polishing autovalidation was applied to further filter the peptide spectrum matches using a target protein-level FDR threshold of 0. Following autovalidation a protein-protein comparison table was generated, which contained experimental over control TMT ratios. For all experiments, non-Drosophila or non-mouse contaminants and reverse hits were removed. Furthermore, data was median normalized. For hemolymph proteomics, MS/MS spectra were analyzed using the Mascot 2.5 search engine by searching fly databases (see below and methods) with a parent ion tolerance of 18 ppm and fragment ion tolerance of 0.80 Da. Carbamidomethylation of Cys (+57.0293 Da) was specified in Sequest as a fixed modification and oxidation of Met (+ 15.9949 Da) and deamidation of Asn/Gln (+ 0.9840 Da) as variable modifications. Results were imported into Scaffold 4.0 software (Proteome Software) with a peptide threshold of around 75%, protein threshold of 95%, resulting in a peptide false discovery rate (FDR) of <1.5%. For confocal images, Where indicated, brightness and contrast were adjusted equally in the whole image and equally between experimental groups using ZEN2012 software (Carl Zeiss). Where indicated, maximum intensity projections were generated using ZEN2012 software (Carl Zeiss). The area of protein aggregates was quantified as previously described. Control and experimental groups of images were analyzed in the same way. Representative single confocal slice images (uncropped) of p62/ref(2)P staining were converted to single color tiff files using ZEN 2012 (Carl Zeiss). Next, using FIJI (FIJI is just image J), images were converted to 8-bit grayscale, and threshold was adjusted in order to use the analyze particles function. The maximum entropy method was used in Fig. 3d, e, g, and h and Supplementary Figure 9w, and the triangle method was used in Fig. 3f. Next, the analyze particles function was used, in which the minimum particle size was 10 pixels2 in Fig. 3d, e, g, and h and Supplementary Figure 9w, and 20 pixels2 in Fig. 3f. Data was analyzed using ZEN2012 software (Carl Zeiss), FIJI, Adobe Photoshop CC2019 (integrated density calculations in Fig. 1d'), Microsoft Excel, Graphpad Prism 7, OriginPro 2017 or 2020, and R (version 4.0.0 (2020-04-24), Platform: x86_64-apple-darwin17.0 (64-bit); RStudio Version 1.3.959). Additionally, SignalP4.1, SignalP5, TMHMM (v 2.0), SecretomeP2.0 were used.

For manuscripts utilizing custom algorithms or software that are central to the research but not yet described in published literature, software must be made available to editors and reviewers. We strongly encourage code deposition in a community repository (e.g. GitHub). See the Nature Research guidelines for submitting code & software for further information.

Data

Policy information about availability of data

All manuscripts must include a data availability statement. This statement should provide the following information, where applicable:

- Accession codes, unique identifiers, or web links for publicly available datasets
- A list of figures that have associated raw data
- A description of any restrictions on data availability

The original mass spectra for all experiments, and the protein sequence databases used for searches have been deposited in the public proteomics repository MassIVE (https://massive.ucsd.edu) and are accessible at https://doi.org/doi:10.25345/C5NB8W (ftp://massive.ucsd.edu/MSV000086664/) (mouse and fly BirA* datasets), and https://doi.org/doi:10.25345/C5XN4Z (ftp://massive.ucsd.edu/MSV000086291/) (fly hemolymph datasets).
The following publicly-available datasets/databases were used: UniProt database(https://www.uniprot.org/; Drosophila (DROME; https://www.uniprot.org/proteomes/UP000000803), mouse (https://www.uniprot.org/proteomes/UP000000589), and human (https://www.uniprot.org/proteomes/UP000005640)), DIOPT (https://www.flyrnai.org/cgi-bin/DRSC_orthologs.pl), GLAD (https://www.flyrnai.org/tools/glad/web/), Signaling Receptome (http://www.receptome.org/), human plasma proteome datasets (http://www.peptideatlas.org/repository/repository_public_Hs_Plasma2.php (PeptideAtlas) and Supplementary Table 1 in ref. 59), SignalP database (http://www.cbs.dtu.dk/services/SignalP/ and http://www.cbs.dtu.dk/services/SignalP-4.1/), Drosophila mitochondrial proteome (Supplementary Dataset 1 in ref. 50), TMHMM (http://www.cbs.dtu.dk/services/TMHMM/), SecretomeP (http://www.cbs.dtu.dk/services/SecretomeP/), FlyAtlas microarray (http://flyatlas.org/atlas.cgi), Drosophila RNAseq (http://www.modencode.org/celniker/), PAXdb (https://pax-db.org/), NCBI Gene (https://www.ncbi.nlm.nih.gov/gene/), FlyBase (https://flybase.org/), mammalian adipocyte secretomes (Tables S1-S5 in ref. 7, Supplemental Data Table C in ref. 68, Supplemental Data Table in ref. 69, Supplemental Table S1 in ref. 70, Supplementary Table – Secretome in ref. 71, Supplementary Table S1 in ref. 72, Data File S1 in ref. 73, Table 2 and Supplementary Table 1 in ref. 75, Supplement Table 2A in ref. 76), mammalian myocyte secretomes (Table 1 in ref. 77, Supplemental Table S1 in ref. 78, Supplemental Table 1 in ref. 79, Supplemental Table 1 in ref. 80, Table 2 in ref. 81, Table S1 in ref. 82, Table S5 in ref. 83, Supplemental Table S1 in ref. 84).

The main text and supplementary information shows all of the data collected as part of this work. The source data are provided with this paper. The corresponding authors will provide original data on reasonable request.

Field-specific reporting

Please select the one below that is the best fit for your research. If you are not sure, read the appropriate sections before making your selection.

☒ Life sciences ☐ Behavioural & social sciences ☐ Ecological, evolutionary & environmental sciences

For a reference copy of the document with all sections, see nature.com/documents/nr-reporting-summary-flat.pdf

Life sciences study design

All studies must disclose on these points even when the disclosure is negative.

Sample size	The sample size was chosen empirically, based on preliminary experiments on flies and mouse teratomas and published studies in the field. The sample sizes are shown in the figures and we in general kept them as large as it was feasible to do for each experiment.

Data exclusions	MS/MS spectra were excluded from searching if they were not within the precursor MH+ range of -600-6000 Da (mouse data) or 750-4000 Da (Drosophila data) or if they failed the quality filter by not having a sequence tag length >0. The rationale behind these exclusions is that these are standard exclusion criteria, and the precursors outside this mass range are typically not peptides. These exclusion criteria were pre-established.
Replication	Experiments were replicated several times with similar results over the course of the project (8 years). We used BirA*R118G and BirA*G3 to obtain similar results (see Supplementary Figures 3 and 7). Shown in top panel of Fig. 1c (Dilp2-HA-biotin ELISA) are representative results from two independent experiments. Fig. 1d is a representative result of 3 western blots. Fig. 1e is a representative result of 3 western blots, including the repeat shown in Supplementary Figure 3a. Multiple RNAi lines were used per gene in Fig. 3. The protein aggregate formation assay in Supplementary Figure 9w was performed by a different investigator and had similar results to Fig. 3. Experiments with different mESC clones also had similar results. Preliminary experiments using mouse teratomas gave similar streptavidin blotting results. Multiple analysis methods for MS data gave similar results (Supplementary Figure 15m-n). In addition, we state in the figure legends or the methods section how many times each experiment was repeated.
Randomization	For studies with and without biotin addition to medium, flies were randomly picked and assigned to experimental groups. For other experiments, this was not relevant, because every experimental group was a different genotype, and all genotypes were treated with the same conditions (eg biotin).
Blinding	Blinding was not performed, as many of the experiments involved computer-automated data acquisition and analysis using mass spectrometry. For Drosophila muscle phenotype experiments, investigators were not blinded to group allocation during data collection and analysis. Blinding was not relevant to this study because these assays were performed as part of a larger study (screen) that included other RNAi lines, and by a different investigator, who was impartial to the results. In addition, before doing the study, we did not know if the RNAi lines (if any) would give a phenotype. With mouse teratoma experiments, investigators were not blinded to group allocation during data collection and analysis. Blinding was not possible, because tumor formation needed to be monitored between BirA+ and BirA- groups, and no difference in tumor formation was observed between Cre+ and Cre- R26 BirA*G3 clones. However, in all cases we followed proper laboratory procedures, including proper controls, and the control and experimental groups were collected under similar conditions.

Reporting for specific materials, systems and methods

We require information from authors about some types of materials, experimental systems and methods used in many studies. Here, indicate whether each material, system or method listed is relevant to your study. If you are not sure if a list item applies to your research, read the appropriate section before selecting a response.

Materials & experimental systems

n/a	Involved in the study	
☐	☒	Antibodies
☒	☐	Eukaryotic cell lines
☒	☐	Palaeontology and archaeology
☐	☒	Animals and other organisms
☒	☐	Human research participants
☒	☐	Clinical data
☒	☐	Dual use research of concern

Methods

n/a	Involved in the study	
☒	☐	ChIP-seq
☒	☐	Flow cytometry
☒	☐	MRI-based neuroimaging

Antibodies

Antibodies used	See material and methods for details. Antibodies and reagents: streptavidin beads (Pierce), 1:200 mouse anti-myc (clone 9E10, Santa Cruz Biotechnology); 1:500 rabbit anti-myc (clone 71D10, Cell Signaling Technology); neat or 1:10 mouse anti-Cnx99A (ER marker; Developmental Studies Hybridoma Bank [DSHB]); 1:2000 rabbit anti-ref(2)P (gift of Andreas Brech); 1:100 mouse anti-ubiquitin (clone FK2, Enzo Life Sciences); 1:500 streptavidin-Alexa Fluor (AF) 647 (Invitrogen); 1:200 rat anti-HA (clone 3F10 [100 µg/mL stock], Roche); and 1:100 phalloidin-AF 660 (Invitrogen). The secondary antibodies and reagents were: 1:500 donkey anti-mouse AF488 (Invitrogen), 1:1,000 goat anti-mouse AF488, 1:500 goat anti-rat AF488 (Invitrogen), 1:500 donkey anti-rabbit AF488 (Invitrogen), 1:500 goat anti-mouse AF555 (Invitrogen), 1:1,000 goat anti-rabbit AF568, 1:500 donkey anti-rabbit AF594 (Invitrogen), 1:500 donkey anti-rabbit AF555 (Invitrogen), 1:500 donkey anti-rat AF594 (Invitrogen), 1:50 goat anti-HRP TRITC (rhodamine; Jackson ImmunoResearch Laboratories 123-025-021), 1:100 phalloidin AF660 (Invitrogen), 1:100 phalloidin AF350 (Invitrogen), 1:100 phalloidin AF546 (Invitrogen), 1:100 streptavidin AF647 (Invitrogen), and 1:2,000 DAPI, and 1:1,000 BODIPY493/503 (Invitrogen). For mouse experiments, Primary antibodies used in the study were as follows: GFP (Aves labs, GFP-1020, 1:500), RFP Tag (Invitrogen, MA5-15257, 1:500), BirA 6C4°C7 (Abcam, ab232732, 1:300-1:500), Alexa Fluor™ Plus 647 Phalloidin (ThermoFisher, A30107, 1:1000). The secondary antibody used were Goat anti-Mouse IgG1 Cross-Adsorbed Secondary Antibody, Alexa Fluor 555 (ThermoFisher, A-21127, 1:1000) Donkey anti-Mouse IgG, Alexa Fluor 594, and Donkey anti-Chicken Alexa Fluor 488), streptavidin DyLight649 conjugated (Vector labs, SA-5649-1, 1:1000). For Drosophila western blots: 1:40,000 streptavidin-HRP (Invitrogen), 1:1,000 mouse anti-myc (clone 9E10, Santa Cruz Biotechnology), 1:2,000 rat anti-HA (clone 3F10, Roche), 1:10,000 mouse anti-tubulin (clone B-5-1-2, Sigma-Aldrich). Secondary antibody: 1:5,000 to 1:10,000 dilution of HRP-conjugated secondary antibody: sheep anti-mouse (GE Healthcare), donkey anti-mouse (Jackson ImmunoResearch), or goat anti-rat (GE Healthcare). For tubulin western blot, a 1:10,000 secondary antibody dilution was used. For mouse experiments: Rb Adiponectin (Abcam, ab181699, 1:1000), Rb C7 (Abcam, ab126786, 1;1000), Rb Apoa1 (Abcam, ab20453, 1:1000), Rb Glucosidase sub-unit beta (Abcam, 134071, 1:1000), Rb Calr (Abcam, ab92516, 1:1000), Rb myc-tag (Abcam, ab9106, 1:1000), Gt Alpl (mouse alkaline phosphatase; R&D systems, AF2910, 1:1000) streptavidin conjugate (1:5,000; 680 or 800, Li-Cor, 926-68079, 926-32230).
Validation	We used antibodies that are commonly used in the field and validated by others:

Validation

Streptavidin beads: https://www.thermofisher.com/order/catalog/product/88816#/88816
rat anti-HA: https://www.sigmaaldrich.com/catalog/product/roche/roahaha?lang=en&region=US
rabbit anti-myc (clone 71D10): https://www.cellsignal.com/products/primary-antibodies/myc-tag-71d10-rabbit-mab/2278?Ntk=Products&Ntt=2278
mouse anti-Cnx99A: Riedel, F., Gillingham, A. K., Rosa-Ferreira, C., Galindo, A. & Munro, S. An antibody toolkit for the study of membrane traffic in Drosophila melanogaster. Biology open, bio. 018937 (2016).
rabbit anti-ref(2)P: Nezis, I. P. et al. Ref (2) P, the Drosophila melanogaster homologue of mammalian p62, is required for the formation of protein aggregates in adult brain. The Journal of cell biology 180, 1065-1071 (2008).
1:100 mouse anti-ubiquitin: Demontis, F. & Perrimon, N. FOXO/4E-BP signaling in Drosophila muscles regulates organism-wide proteostasis during aging. Cell 143, 813-825 (2010).
phalloidin AF660: https://www.thermofisher.com/order/catalog/product/A22285#/A22285
streptavidin-Alexa Fluor (AF) 647: https://www.thermofisher.com/order/catalog/product/S21374#/S21374
GFP (Aves labs, GFP-1020): https://www.aveslabs.com/products/green-fluorescent-protein-gfp-antibody
RFP Tag (Invitrogen, MA5-15257): https://www.thermofisher.com/antibody/product/RFP-Antibody-clone-RF5R-Monoclonal/MA5-15257
BirA 6C4°C7: We validated its ability to detect BirA*G3.
streptavidin-HRP: https://www.thermofisher.com/order/catalog/product/SA10001#/SA10001
mouse anti-myc (clone 9E10): https://www.sigmaaldrich.com/catalog/product/sigma/m4439?lang=en&region=US
mouse anti-tubulin (clone B-5-1-2): https://www.sigmaaldrich.com/catalog/product/sigma/t5168?lang=en&region=US
Rb Adiponectin (Abcam, ab181699): https://www.abcam.com/adiponectin-antibody-19f1-ab22554.html
Rb C7 (Abcam, ab126786, 1;1000): https://www.abcam.com/c7-antibody-epr7036-ab126786.html
Rb Apoa1 (Abcam, ab20453): https://www.abcam.com/apolipoprotein-a-i-antibody-ab20453.html
Rb Glucosidase sub-unit beta (Abcam, 134071): https://www.abcam.com/glucosidase-2-subunit-beta-antibody-epr8046-ab134071.html
Rb Calr (Abcam, ab92516, 1:1000): https://www.abcam.com/calreticulin-antibody-epr3924-er-marker-ab92516.html
Rb myc-tag (Abcam, ab9106): https://www.abcam.com/myc-tag-antibody-ab9106.html
Gt Alpl (mouse alkaline phosphatase; R&D systems, AF2910): https://www.rndsystems.com/products/mouse-alkaline-phosphatase-alpl-antibody_af2910
The secondary antibodies have been extensively verified by others in the field.

Animals and other organisms

Policy information about studies involving animals; ARRIVE guidelines recommended for reporting animal research

Laboratory animals

For the Drosophila part of the study, strain genotypes are shown in Supplementary Table 1, and full genotypes, sexes, ages, and numbers are listed in the Figure captions, main text, or materials and methods. For RNAi experiments, both males and females were used, with ages listed in the figure (3-5 weeks old). For CG2145 binding experiments, approximately 1 week old males were used. Both males and females were used for BirA*-labeling MS experiments; these were approximately 2 weeks old. Both males and females were used for Dilp2 ELISA experiments. For hemolymph mass spectrometry experiments, data included both males and females; the experiments used younger (approximately 1-3 weeks) and older (4 week old) flies. For aging experiments, both males and females were used. For climbing experiments during aging studies, 3 week old flies (males and females) were used. Approximately 2 week old males were used in fat body to brain communication experiments, and approximately 2 week old females were used in hemolymph myc and streptavidin blots in Supplementary Figure 3c-d; males and females (approximately 2 week old) were used in Supplementary Figure 3d'.

For the mouse part of the study, week C57Bl/6N male mice (Charles Rivers) were used in surgeries, and were 18 weeks old at collection. The mouse vivaria are on a 14-10 hour light-dark cycle. The lights turn on at 5 am and turn off at 7 pm. The ambient temperature is kept between 70-75 degrees F and the ambient humidity is 30-70%. Note that the B6(Cg)-Tyr<c-2J>/J were used as an embryonic stem cell line, and not as mice, in this study.

Wild animals

No wild animals were involved in this work.

Field-collected samples

No field-collected samples were involved in this work.

Ethics oversight

All surgeries and animal work was carried out according to federal and institutional guidelines, animal protocols covering the work in APM's laboratory were approved by the University of Southern California's IACUC committee. No ethical approval or guidance was required for the Drosophila part of the study.

Note that full information on the approval of the study protocol must also be provided in the manuscript.
